# Non-nuclear Pool of Splicing Factor SFPQ Regulates Axonal Transcripts Required for Normal Motor Development

**DOI:** 10.1016/j.neuron.2017.04.036

**Published:** 2017-05-17

**Authors:** Swapna Thomas-Jinu, Patricia M. Gordon, Triona Fielding, Richard Taylor, Bradley N. Smith, Victoria Snowden, Eric Blanc, Caroline Vance, Simon Topp, Chun-Hao Wong, Holger Bielen, Kelly L. Williams, Emily P. McCann, Garth A. Nicholson, Alejandro Pan-Vazquez, Archa H. Fox, Charles S. Bond, William S. Talbot, Ian P. Blair, Christopher E. Shaw, Corinne Houart

(Neuron *94*, 322–336; April 19, 2017)

As a result of an author oversight in the originally published version of this article, the first name of author Kelly L. Williams was written as “Katherine.” This error has now been corrected in the article online. The authors apologize for the error.

