# Supplementary figures and images for: Non-nuclear Pool of Splicing Factor SFPQ Regulates Axonal Transcripts Required for Normal Motor Development
Source: Neuron. 2017 Apr 19;94(2):322–336.e5. doi: 10.1016/j.neuron.2017.03.026 (PMC5405110; doi:10.1016/j.neuron.2017.03.026)

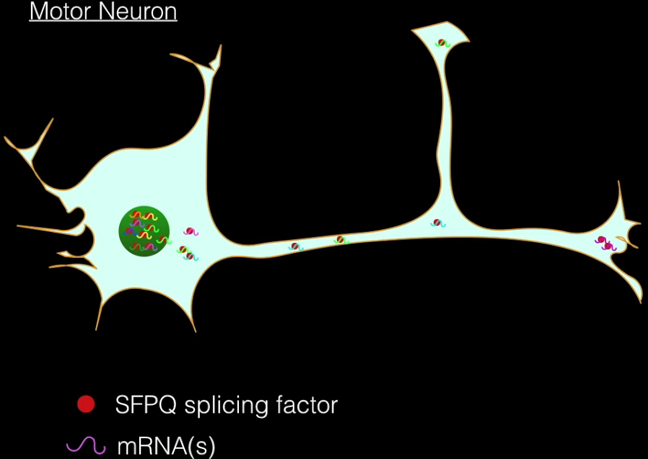

Supplement: Supplementary file 1 [file mmc6.jpg]

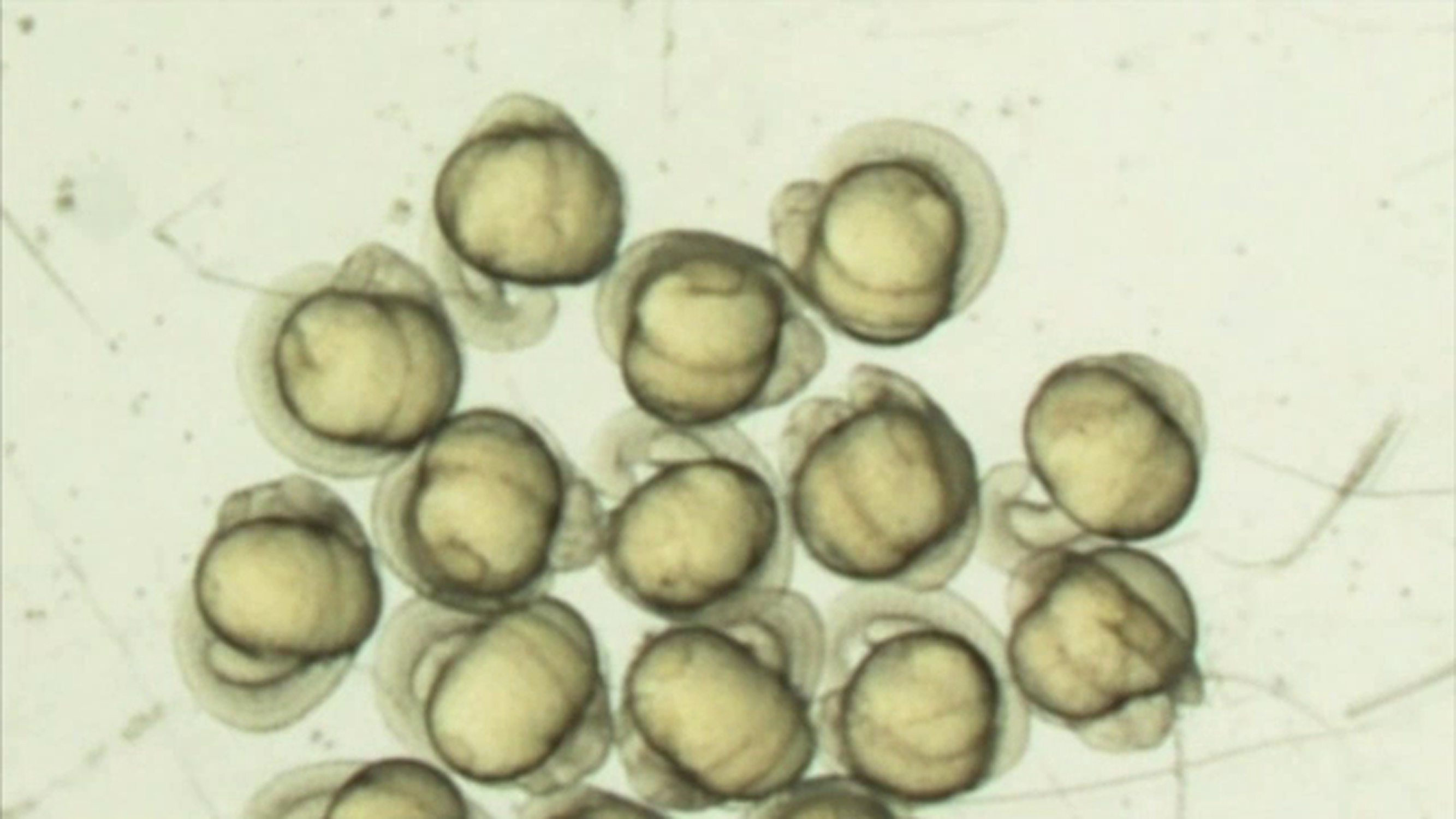

Supplement: Movie S1. Video of 18–20 Somite Stage Embryos from a sfpq+/− Heterozygous Cross, Showing Spontaneous Movement in Siblings and No Movement at All in Homozygous Embryos from the Same Clutch, Related to Figure 1 [file mmc2.jpg]

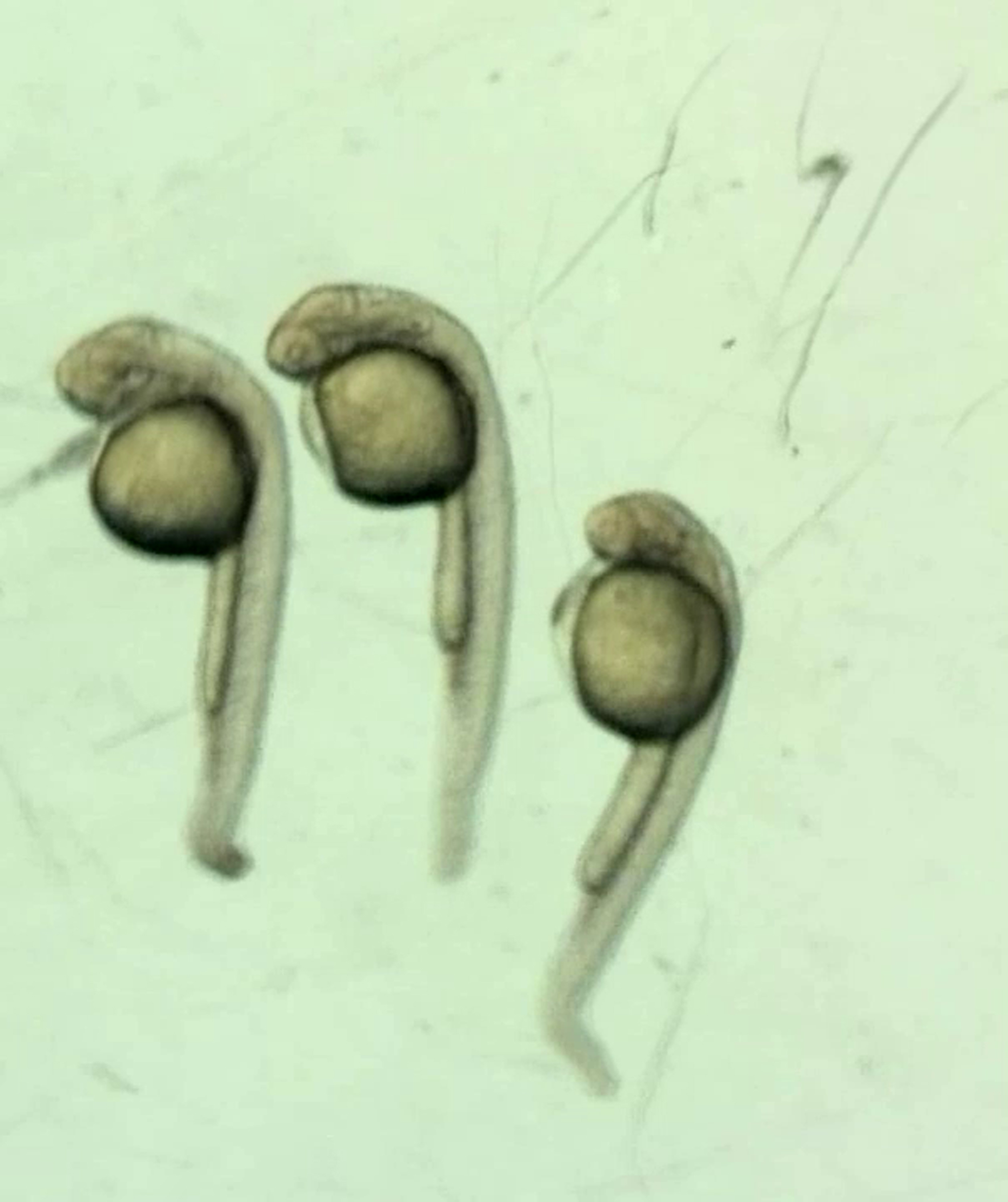

Supplement: Movie S2. Video Showing Movement in 30 hpf sfpq−/− Embryos in Tg(Xla.Tubb2b:Hsa.MAPT-GFP)zc1 Background, Related to Figure 2 [file mmc3.jpg]

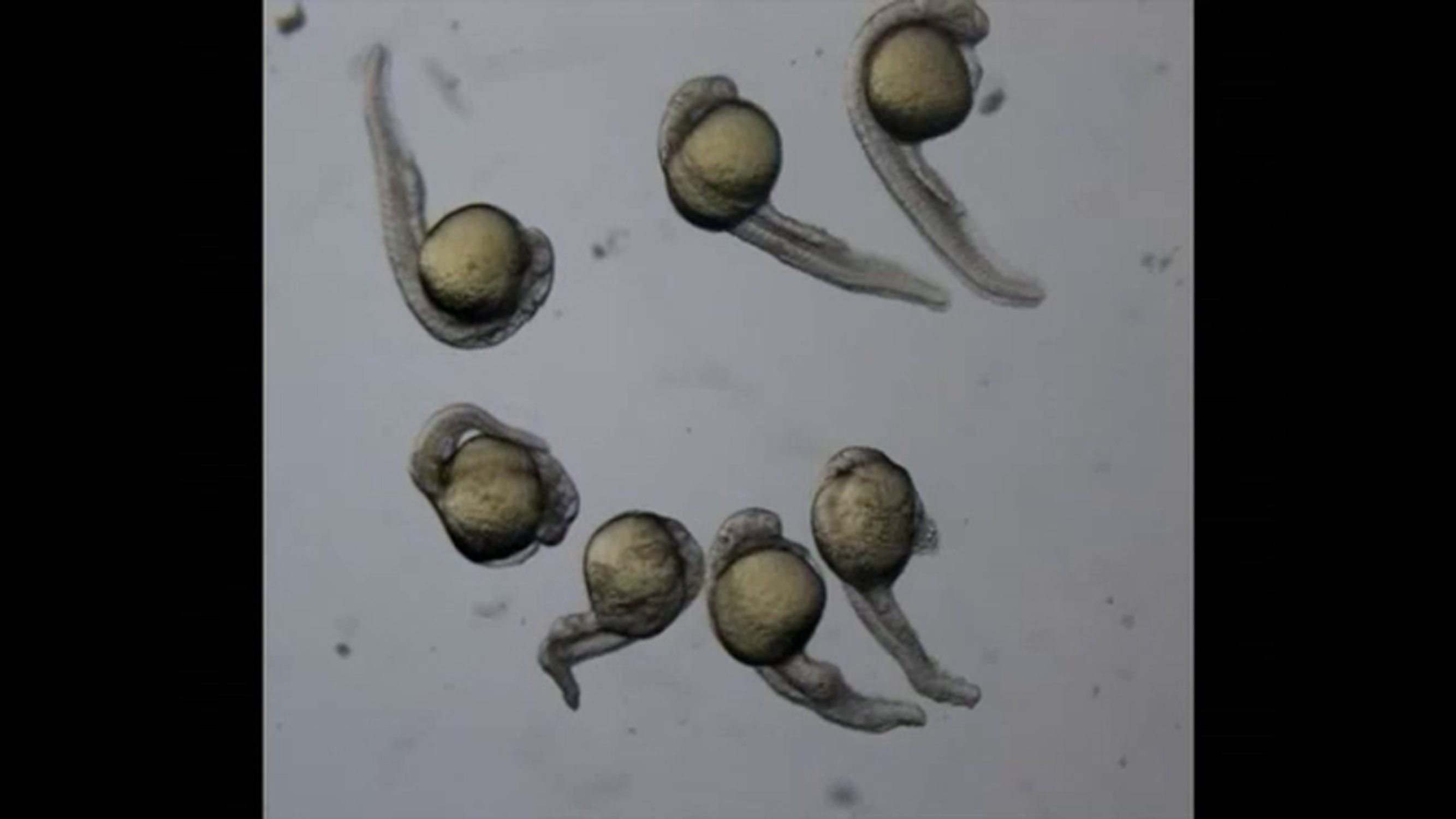

Supplement: Movie S3. Motility and Morphology of 30 hpf sfpq−/− Embryos Rescued by Injection of Full-Length or ΔNLS Human sfpq RNA, Related to Figure 6 [file mmc4.jpg]
