# Supplementary material for: Non-nuclear Pool of Splicing Factor SFPQ Regulates Axonal Transcripts Required for Normal Motor Development
Source: Neuron. 2017 Apr 19;94(2):322–336.e5. doi: 10.1016/j.neuron.2017.03.026 (PMC5405110; doi:10.1016/j.neuron.2017.03.026)
Supplement: Document S1. Figures S1–S3 and Tables S1–S4 [file mmc1.pdf]

Neuron, Volume *94*

Supplemental Information

Non-nuclear Pool of Splicing Factor SFPQ Regulates Axonal Transcripts Required for Normal Motor Development

Swapna Thomas-Jinu, Patricia M. Gordon, Triona Fielding, Richard Taylor, Bradley N. Smith, Victoria Snowden, Eric Blanc, Caroline Vance, Simon Topp, Chun-Hao Wong, Holger Bielen, Kelly L. Williams, Emily P. McCann, Garth A. Nicholson, Alejandro Pan-Vazquez, Archa H. Fox, Charles S. Bond, William S. Talbot, Ian P. Blair, Christopher E. Shaw, and Corinne Houart

Supplemental Table 1. SFPQ-dependent transcripts with direct homologs in mouse and human.

actn2	IGLON5	TUSC3	cacna1i	efna5b	gpc3	ncam1b	poc1b	sema5a	uvrag
arvcfb	igsf21a	vti1a	cadm1a	enox1	grid2	ncam2	ppm1e	sema5b	VAV2
astn1	iqsec3a	ank2b	cadm1b	epha6	grin3b	nck2b	prcp	SEMA6B	wwox
bcas3	kcnab1	abca2	cadm2a	EPHB1	Gulp1b	NDST2	prkca	slc25a21	xkr7
BRSK2	Lphn1	acap3a	camta1	erbb4a	HS2ST1	neto1l	prkd2	slit3	zgc:65851
cacna2d2	lrp1bb	ADAMTSL3	camta1a	erbb4b	htr2cl1	nfixb	prkg1b	slx4ip	znf385b
cacng2a	magi2	adarb1b	celf5	fam172a	inadl	nitr3c	ptbp3	smyd3	znf804b
cadm4	magi3	adck1	chrm3a	fam184a	Kazn	nitr3d	PTCHD2	snx29	
cask	nbeaa	adka	CLMP	fam189a1	kcnh3	nlgn4a	ptprea	spon1a	
cdh13	ncam1a	agbl4	cntfr	fancd2	kiaa0586	nphp4	ptprga	spon1b	
cdh4	ncanb	akt3a	cntn5	fars2	KIRREL3	ntrk3a	ptprk	stat5.1	
cpne5	necab2	AL935143.2	col23a1	FBXL17	KLHL29	nudt14	ptprua	strbp	
ctnna2	negr1	apba2b	col4a6	fgf13a	lhfpl3	odz2	ptprub	stx8	
ctnnd2a	nlgn3a	ARHGAP42	crfb4	FHOD3	loxl3b	odz3	ptprub	sulf2l	
dbn1	nrxn2a	arhgef12b	crim1	fign	lpp	oprl1	PXN	tbc1d22a	
dgkza	nrxn3b	ARID5B	CU633160	Frmd5	lrfn2	osbp2	rad51b	tbxas1	
dlg1	ntm	asic1b	cux2b	fstl1b	LRFN3	pacrg	ralgapa2	tcf12	
DLG4	olfm2a	asic1c	dab1a	fstl4	lrp8	pard3	ralgps1	thrab	
dlgap1b	opcml	asic2	dacha	fstl5	lrrn2	pard3b	rbfox1	thrb	
dpp6b	pcdh7b	asic4a	dachd	galnt1	macrod2	pcdh15a	rbms3	tmtc2	
**dync1l1**	plekha5	B3GALT1	dapk2	galnt2	magi1b	pcl	rgs6	tnr	
EPB41L1	ptprsa	b3gat1	def8	galt	map2	PDE1A	rims3	TOX2	
fut8	ptprt	bcl2,bcl2	dgat1b	GBF1	mb21d2b	piezo1	robo3	tp53i11b	
gabbr2	rab6ba	bco2b	dip2c	gdf11	megf11	pkib	ror1	tp53i11a	
gnao1a	raph1	becn1	dph1	gfra1a	mgat4c	pkig	rtn4rl1b	trpm3	
gng7	rtn4r	BX890577.1	draxin	gfra4	mid2	pknox2	rxraa	trps1	
gria3b	sfpq	C13H10orf11	ebf1b	ggt1a	mpp7	plch2a	samd10b	tspan9a	
gria4b	slc8a2b	C20orf112	edil3	gli2a	msi2a	PLXNA2	scfd2	unc5da	
grip2b	syt1a	CA10	efcab11	gli3	msi2b	plxnb1a	sdk1b	unc5db	
hpca	TLN2	CABZ01062452	efna2	glra4a	myrip	plxnb2b	sdk2b	ust	

Supplemental Table 2: Enriched GO terms using LAGO

GOID	Term	P-value	Uncorrected P-value	Number annotated	Annotated genes
GO:0007399	nervous system development	1.96E-11	1.73E-14	33	asic2, bcl2, CA10, cdh4, cpne5, dlg1, draxin, efna2, fstl4, gfra1, gli2, hpca, Lphn1, lrfn2, LRFN3, macrod2, ncan, nck2, negr1, nlgn3, nlgn4, ntm, odz2, odz3, opcml, PLXNA2, plxnb1,
GO:0048666	neuron development	7.56E-11	6.68E-14	23	bcl2, cdh4, cpne5, draxin, efna2, fstl4, gli2, lrfn2, LRFN3, nck2, negr1, nlgn3, ntm, odz2,
GO:0061564	axon development	1.45E-10	1.28E-13	17	bcl2, cdh4, draxin, efna2, fstl4, gli2, lrfn2, LRFN3, nlgn3, odz2, PLXNA2, plxnb1, rtn4r, rxra,
GO:0030182	neuron differentiation	1.45E-10	1.28E-13	25	bcl2, cdh4, cpne5, draxin, efna2, fstl4, gli2, lrfn2, LRFN3, nck2, negr1, nlgn3, nlgn4, ntm, odz2, odz3, opcml, PLXNA2, plxnb1, PTCHD2, rtn4r, rxra, sema5, slit3, ust
GO:0051960	regulation of nervous system development	2.51E-10	2.22E-13	20	asic2, bcl2, cdh4, cpne5, dlg1, draxin, fstl4, gli2, Lphn1, negr1, nlgn3, odz3, PLXNA2, plxnb1, PTCHD2, rtn4r, rxra, sema5, slit3, ust
GO:0031175	neuron projection development	2.65E-10	2.34E-13	21	bcl2, cdh4, cpne5, draxin, efna2, fstl4, gli2, lrfn2, LRFN3, nck2, negr1, nlgn3, odz2, odz3,
GO:0007409	axonogenesis	6.04E-10	5.34E-13	16	bcl2, cdh4, draxin, efna2, fstl4, gli2, lrfn2, LRFN3, nlgn3, odz2, PLXNA2, plxnb1, rtn4r,
GO:0048699	generation of neurons	9.48E-10	8.38E-13	25	bcl2, cdh4, cpne5, draxin, efna2, fstl4, gli2, lrfn2, LRFN3, nck2, negr1, nlgn3, nlgn4, ntm, odz2, odz3, opcml, PLXNA2, plxnb1, PTCHD2, rtn4r, rxra, sema5, slit3, ust
GO:0048812	neuron projection morphogenesis	3.42E-09	3.02E-12	17	bcl2, cdh4, cpne5, draxin, efna2, fstl4, gli2, lrfn2, LRFN3, nlgn3, odz2, PLXNA2, plxnb1, rtn4r, sema5, slit3, ust
GO:0022008	neurogenesis	3.91E-09	3.46E-12	25	bcl2, cdh4, cpne5, draxin, efna2, fstl4, gli2, lrfn2, LRFN3, nck2, negr1, nlgn3, nlgn4, ntm, odz2, odz3, opcml, PLXNA2, plxnb1, PTCHD2, rtn4r, rxra, sema5, slit3, ust
GO:0048468	cell development	4.19E-09	3.70E-12	28	bcl2, cacna2d2, cdh4, cpne5, draxin, efna2, fstl4, gdf1, gli2, lrfn2, LRFN3, nck2, negr1, nlgn3, ntm, odz2, odz3, opcml, PLXNA2, plxnb1, PTCHD2, rtn4r, rxra, sema5, slit3, strbp, ust,
GO:0048858	cell projection morphogenesis	5.54E-09	4.90E-12	17	bcl2, cdh4, cpne5, draxin, efna2, fstl4, gli2, lrfn2, LRFN3, nlgn3, odz2, PLXNA2, plxnb1, rtn4r,
GO:0032990	cell part morphogenesis	9.04E-09	7.99E-12	17	bcl2, cdh4, cpne5, draxin, efna2, fstl4, gli2, lrfn2, LRFN3, nlgn3, odz2, PLXNA2, plxnb1, rtn4r,
GO:0048667	cell morphogenesis involved in neuron differentiation	9.45E-09	8.35E-12	16	bcl2, cdh4, draxin, efna2, fstl4, gli2, lrfn2, LRFN3, nlgn3, odz2, PLXNA2, plxnb1, rtn4r, sema5, slit3, ust
GO:0030030	cell projection organization	1.81E-08	1.60E-11	23	bcl2, cdh4, cpne5, draxin, efna2, fstl4, gli2, lrfn2, LRFN3, nck2, negr1, nlgn3, odz2, odz3,
GO:0048589	developmental growth	1.82E-08	1.60E-11	16	bcl2, cacna2d2, cdh4, cpne5, draxin, fstl4, gli2, nlgn3, nlgn4, PLXNA2, plxnb1, rtn4r, rxra,
GO:0000904	cell morphogenesis involved in differentiation	1.83E-08	1.62E-11	17	bcl2, cdh4, draxin, efna2, fstl4, gli2, lrfn2, LRFN3, nlgn3, odz2, PLXNA2, plxnb1, rtn4r, sema5, slit3, ust, znf385
GO:0045664	regulation of neuron differentiation	2.11E-08	1.87E-11	16	bcl2, cdh4, cpne5, draxin, fstl4, gli2, negr1, nlgn3, odz3, PLXNA2, plxnb1, PTCHD2, rtn4r, sema5, slit3, ust
GO:0000902	cell morphogenesis	6.82E-08	6.03E-11	19	bcl2, cdh4, cpne5, dlg1, draxin, efna2, fstl4, gli2, lrfn2, LRFN3, nlgn3, odz2, PLXNA2, plxnb1,
GO:0048588	developmental cell growth	2.74E-07	2.42E-10	10	cdh4, cpne5, draxin, fstl4, nlgn3, PLXNA2, plxnb1, rtn4r, sema5, slit3
GO:0050767	regulation of neurogenesis	3.02E-07	2.67E-10	16	bcl2, cdh4, cpne5, draxin, fstl4, gli2, negr1, nlgn3, odz3, PLXNA2, plxnb1, PTCHD2, rtn4r,
GO:0032989	cellular component morphogenesis	3.17E-07	2.80E-10	19	bcl2, cdh4, cpne5, dlg1, draxin, efna2, fstl4, gli2, lrfn2, LRFN3, nlgn3, odz2, PLXNA2, plxnb1, rtn4r, sema5, slit3, ust, znf385
GO:0010975	regulation of neuron projection development	4.54E-07	4.01E-10	13	cdh4, cpne5, draxin, fstl4, negr1, nlgn3, odz3, PLXNA2, plxnb1, rtn4r, sema5, slit3, ust
GO:1990138	neuron projection extension	6.52E-07	5.76E-10	9	cdh4, cpne5, draxin, nlgn3, PLXNA2, plxnb1, rtn4r, sema5, slit3
GO:0060560	developmental growth involved in morphogenesis	8.38E-07	7.41E-10	10	cdh4, cpne5, draxin, fstl4, nlgn3, PLXNA2, plxnb1, rtn4r, sema5, slit3
GO:0031344	regulation of cell projection organization	1.32E-06	1.17E-09	14	cdh4, cpne5, draxin, fstl4, negr1, nlgn3, odz2, odz3, PLXNA2, plxnb1, rtn4r, sema5, slit3, ust
GO:0050770	regulation of axonogenesis	1.80E-06	1.59E-09	9	cdh4, draxin, fstl4, PLXNA2, plxnb1, rtn4r, sema5, slit3, ust
GO:0060284	regulation of cell development	2.03E-06	1.80E-09	16	bcl2, cdh4, cpne5, draxin, fstl4, gli2, negr1, nlgn3, odz3, PLXNA2, plxnb1, PTCHD2, rtn4r,
GO:0048675	axon extension	2.24E-06	1.98E-09	8	cdh4, draxin, nlgn3, PLXNA2, plxnb1, rtn4r, sema5, slit3
GO:0050793	regulation of developmental process	2.54E-06	2.25E-09	26	asic2, bcl2, cacna2d2, cdh4, cpne5, dlg1, draxin, fstl4, gli2, htr2c, Lphn1, negr1, nlgn3, odz3, olfm2, PLXNA2, plxnb1, PTCHD2, ror1, rtn4r, rxra, sema5, slit3, stat5, ust, znf385
GO:0007155	cell adhesion	3.32E-06	2.93E-09	23	bcl2, cadm2, cadm4, cdh4, cntn5, dlg1, edil3, gli2, Lphn1, LRFN3, megf11, ncan, nck2, negr1, nlgn3, nlgn4, ntm, odz2, odz3, opcml, plxnb1, ptpru, spon1
GO:0022610	biological adhesion	3.56E-06	3.15E-09	23	bcl2, cadm2, cadm4, cdh4, cntn5, dlg1, edil3, gli2, Lphn1, LRFN3, megf11, ncan, nck2, negr1, nlgn3, nlgn4, ntm, odz2, odz3, opcml, plxnb1, ptpru, spon1
GO:0010769	regulation of cell morphogenesis	7.70E-06	6.81E-09	10	cdh4, draxin, fstl4, nlgn3, PLXNA2, plxnb1, rtn4r, sema5, slit3, ust
GO:0048869	cellular developmental process	8.46E-06	7.48E-09	34	bcl2, cacna2d2, cdh4, cpne5, dlg1, draxin, efna2, fstl4, gdf1, gli2, htr2c, Kazn, lrfn2, LRFN3, nck2, negr1, nlgn3, nlgn4, ntm, odz2, odz3, olfm2, opcml, PLXNA2, plxnb1, PTCHD2, ptpru,
GO:0030154	cell differentiation	8.95E-06	7.92E-09	33	bcl2, cacna2d2, cdh4, cpne5, draxin, efna2, fstl4, gdf1, gli2, htr2c, Kazn, lrfn2, LRFN3, nck2, negr1, nlgn3, nlgn4, ntm, odz2, odz3, olfm2, opcml, PLXNA2, plxnb1, PTCHD2, ptpru, rtn4r,
GO:0022604	regulation of cell morphogenesis	1.02E-05	9.02E-09	12	cdh4, cpne5, dlg1, draxin, fstl4, nlgn3, PLXNA2, plxnb1, rtn4r, sema5, slit3, ust
GO:0048638	regulation of developmental growth	1.80E-05	1.59E-08	10	bcl2, cacna2d2, cdh4, cpne5, draxin, fstl4, PLXNA2, rtn4r, sema5, stat5
GO:0051239	regulation of multicellular organismal process	1.94E-05	1.72E-08	27	asic2, bcl2, cacna2d2, cacng2, cdh4, cpne5, dlg1, draxin, fstl4, gfra4, gli2, htr2c, Lphn1, negr1, nlgn3, odz3, PLXNA2, plxnb1, PTCHD2, ror1, rtn4r, rxra, sema5, slit3, stat5, ust, VAV2
GO:2000026	regulation of multicellular organismal development	3.87E-05	3.42E-08	21	asic2, bcl2, cdh4, cpne5, dlg1, draxin, fstl4, gli2, Lphn1, negr1, nlgn3, odz3, PLXNA2, plxnb1, PTCHD2, ror1, rtn4r, rxra, sema5, slit3, ust
GO:0040007	growth	5.25E-05	4.64E-08	16	bcl2, cacna2d2, cdh4, cpne5, draxin, fstl4, gli2, nlgn3, nlgn4, PLXNA2, plxnb1, rtn4r, rxra,
GO:0023052	signaling	0.00010118	8.95E-08	51	akt3, asic2, bcl2, cacna1i, cacna2d2, cacng2, crfb4, dlg1, draxin, efna2, fstl1, fstl4, gabbr2, GBF1, gdf1, gfra1, gfra4, gli2, glra4, gria3, Gulp1, hpca, htr2c, inadl, iqsec3, kcnh3, Lphn1, lrfn2, LRFN3, magi3, nck2, nlgn3, nlgn4, odz2, odz3, PDE1A, PLXNA2, plxnb1, PTCHD2,
GO:0099537	trans-synaptic signaling	0.00010472	9.26E-08	13	asic2, cacna2d2, dlg1, gabbr2, glra4, gria3, htr2c, Lphn1, lrfn2, LRFN3, nlgn3, nlgn4, rims3
GO:0007268	chemical synaptic transmission	0.00010472	9.26E-08	13	asic2, cacna2d2, dlg1, gabbr2, glra4, gria3, htr2c, Lphn1, lrfn2, LRFN3, nlgn3, nlgn4, rims3
GO:0099536	synaptic signaling	0.00010472	9.26E-08	13	asic2, cacna2d2, dlg1, gabbr2, glra4, gria3, htr2c, Lphn1, lrfn2, LRFN3, nlgn3, nlgn4, rims3
GO:0098916	anterograde trans-synaptic signaling	0.00010472	9.26E-08	13	asic2, cacna2d2, dlg1, gabbr2, glra4, gria3, htr2c, Lphn1, lrfn2, LRFN3, nlgn3, nlgn4, rims3
GO:0007154	cell communication	0.00010515	9.30E-08	51	akt3, asic2, bcl2, cacna1i, cacna2d2, cacng2, crfb4, dlg1, draxin, efna2, fstl1, fstl4, gabbr2, GBF1, gdf1, gfra1, gfra4, gli2, glra4, gria3, Gulp1, hpca, htr2c, inadl, iqsec3, kcnh3, Lphn1, lrfn2, LRFN3, magi3, nck2, nlgn3, nlgn4, odz2, odz3, PDE1A, PLXNA2, plxnb1, PTCHD2,
GO:0007411	axon guidance	0.00011775	1.04E-07	9	cdh4, draxin, efna2, gli2, odz2, PLXNA2, plxnb1, sema5, slit3
GO:0097485	neuron projection guidance	0.00012538	1.11E-07	9	cdh4, draxin, efna2, gli2, odz2, PLXNA2, plxnb1, sema5, slit3
GO:0045595	regulation of cell differentiation	0.00013032	1.15E-07	19	bcl2, cdh4, cpne5, draxin, fstl4, gli2, htr2c, negr1, nlgn3, odz3, olfm2, PLXNA2, plxnb1,
GO:0022603	regulation of anatomical structure morphogenesis	0.00020497	1.81E-07	15	bcl2, cdh4, cpne5, dlg1, draxin, fstl4, nlgn3, PLXNA2, plxnb1, ror1, rtn4r, rxra, sema5, slit3, ust
GO:0009653	anatomical structure morphogenesis	0.00029621	2.62E-07	24	bcl2, cdh4, cpne5, dlg1, draxin, efna2, fstl4, gli2, lrfn2, LRFN3, megf11, nlgn3, odz2, odz3, PLXNA2, plxnb1, ror1, rtn4r, rxra, sema5, slit3, ust, VAV2, znf385
GO:0007157	heterophilic cell-cell adhesion via plasma membrane cell adhesion molecules	0.00037265	3.29E-07	5	cadm2, cadm4, cdh4, Lphn1, odz2
GO:0016049	cell growth	0.00053167	4.70E-07	11	bcl2, cdh4, cpne5, draxin, fstl4, nlgn3, PLXNA2, plxnb1, rtn4r, sema5, slit3
GO:0098609	cell-cell adhesion	0.00063662	5.63E-07	15	bcl2, cadm2, cadm4, cdh4, dlg1, gli2, Lphn1, megf11, nck2, negr1, nlgn3, nlgn4, odz2, odz3,

GO:0007267	cell-cell signaling	0.00064232	5.68E-07	19	asic2, cacna2d2, dlg1, draxin, efna2, gabbr2, gli2, glra4, gria3, htr2c, Lphn1, lrfn2, LRFN3,
GO:0061387	regulation of extent of cell growth	0.00064778	5.73E-07	6	cdh4, draxin, fstl4, PLXNA2, rtn4r, sema5
GO:0007165	signal transduction	0.00073198	6.47E-07	47	akt3, asic2, bcl2, cacna1i, cacng2, crfb4, dlg1, draxin, efna2, fstl1, fstl4, gabbr2, GBF1, gdf1, gfra1, gfra4, gli2, glra4, gria3, Gulp1, hpca, htr2c, inadl, iqsec3, kcnh3, Lphn1, magi3, nck2, nlgn3, nlgn4, odz2, odz3, PDE1A, PLXNA2, plxnb1, PTCHD2, ptpru, rab6, ror1, rtn4r, rxra,
GO:0008361	regulation of cell size	0.00106436	9.41E-07	7	cdh4, draxin, fstl4, PLXNA2, rtn4r, sema5, VAV2
GO:0042391	regulation of membrane potential	0.00137687	1.22E-06	9	asic2, bcl2, cacna1i, cacng2, dlg1, kcnh3, nlgn3, nlgn4, rims3
GO:0051962	positive regulation of nervous system development	0.00141819	1.25E-06	10	asic2, bcl2, cdh4, cpne5, gli2, Lphn1, negr1, nlgn3, odz3, plxnb1
GO:0007166	cell surface receptor signaling pathway	0.00196646	1.74E-06	27	bcl2, cacng2, crfb4, draxin, efna2, fstl1, fstl4, gdf1, gfra1, gfra4, gli2, glra4, gria3, Lphn1, nck2, nlgn3, nlgn4, PLXNA2, plxnb1, PTCHD2, ptpru, ror1, rtn4r, sema5, slit3, stat5, VAV2
GO:0040011	locomotion	0.0026817	2.37E-06	18	adarb1, bcl2, cacna1i, cdh4, dach, draxin, efna2, GBF1, gli2, nck2, odz2, PLXNA2, plxnb1,
GO:0051128	regulation of cellular component organization	0.00307511	2.72E-06	22	asic2, bcl2, cdh4, cpne5, dlg1, draxin, fstl4, hpca, Lphn1, nck2, negr1, nlgn3, odz2, odz3, PLXNA2, plxnb1, rtn4r, sema5, sfpq, slit3, ust, vti1
GO:0001558	regulation of cell growth	0.00454863	4.02E-06	9	bcl2, cdh4, cpne5, draxin, fstl4, PLXNA2, rtn4r, sema5, slit3
GO:0040008	regulation of growth	0.00461023	4.08E-06	11	bcl2, cacna2d2, cdh4, cpne5, draxin, fstl4, PLXNA2, rtn4r, sema5, slit3, stat5
GO:0071840	cellular component organization or biogenesis	0.00494186	4.37E-06	44	akt3, asic2, bcl2, cacna2d2, cacng2, cadm2, cdh4, cpne5, dach, dgat1, dlg1, draxin, efna2, fign, fstl4, GBF1, gli2, Gulp1, hpca, inadl, iqsec3, Lphn1, lrfn2, LRFN3, ncan, nck2, negr1,
GO:0045666	positive regulation of neuron differentiation	0.00630851	5.58E-06	8	bcl2, cdh4, cpne5, gli2, negr1, nlgn3, odz3, plxnb1
GO:0030516	regulation of axon extension	0.0073925	6.54E-06	5	cdh4, draxin, PLXNA2, rtn4r, sema5
GO:0008037	cell recognition	0.00741104	6.55E-06	6	cadm2, cadm4, dlg1, nck2, ntm, opcml
GO:0032535	regulation of cellular component size	0.00793873	7.02E-06	9	cdh4, dlg1, draxin, fstl4, nck2, PLXNA2, rtn4r, sema5, VAV2
GO:0010771	negative regulation of cell morphogenesis	0.00869773	7.69E-06	5	draxin, fstl4, nlgn3, rtn4r, sema5
GO:0006928	movement of cell or subcellular component	0.00949934	8.40E-06	19	adarb1, bcl2, cacna1i, cdh4, dach, draxin, efna2, GBF1, gli2, nck2, odz2, PLXNA2, plxnb1, ptpru, rab6, sema5, slit3, stat5, VAV2

Supplemental Table 3: Expression of mature and intronic RNA of neuronal transcripts

	Gene name	Intronic expression	Mature mRNA expression
**ank2b**	Ankyrin 2b	commissures, radial glia, spinal motor axons	Pan-CNS
**bcas3**	Breast carcinoma amplified 3 (cytoskeletal protein)	spinal neurites and telencephalon	CNS, muscle
**cadm4**	Cadherin molecule 4	motor axons	Pan-CNS
**dlg4**	Disc large homolog 4 (=postsynaptic density 95)	commisures, forebrain & spinal neurites	Pan-CNS neurites
**fut8a**	Fucosyltransferase 8		ubiquitous
**gnao1a**	Guanine nucleotide binding protein1a	commissures, diencephalon, spinal	Pan-CNS
**grip2b**	Glutamate receptor interacting protein 2b	muscle, weak neurites in telencephalon	CNS and somitic muscle
**ncam1b**	Neural cell adhesion molecule1b	weak spinal and forebrain neurites	Pan-CNS
**oprl1**	Opiate receptor like 1	weak in all neuronal cell bodies	Pan-CNS
**tln2**	Talin 2 (cytoskeletal)	telencephalon only	Telencephalon, cerebellum
**nbeaa**	neurobeachin	Cell body in spinal cord, hindbrain, sparse in forebrain	Pan-neuronal

Supplemental Table 4: Dosage series of human SFPQ RNA injection in the zebrafish mutant

pg of *h-sfpq* injected per z-sfpq$^{-/-}$ mutant	Axonal rescue	Motility	qPCR rescue	Nuclear GFP detection
25	-	-	-	-
50	-	-	-	+
100	+	55%	+	+
150	+	100%	+	+

Suppl. Figure 1

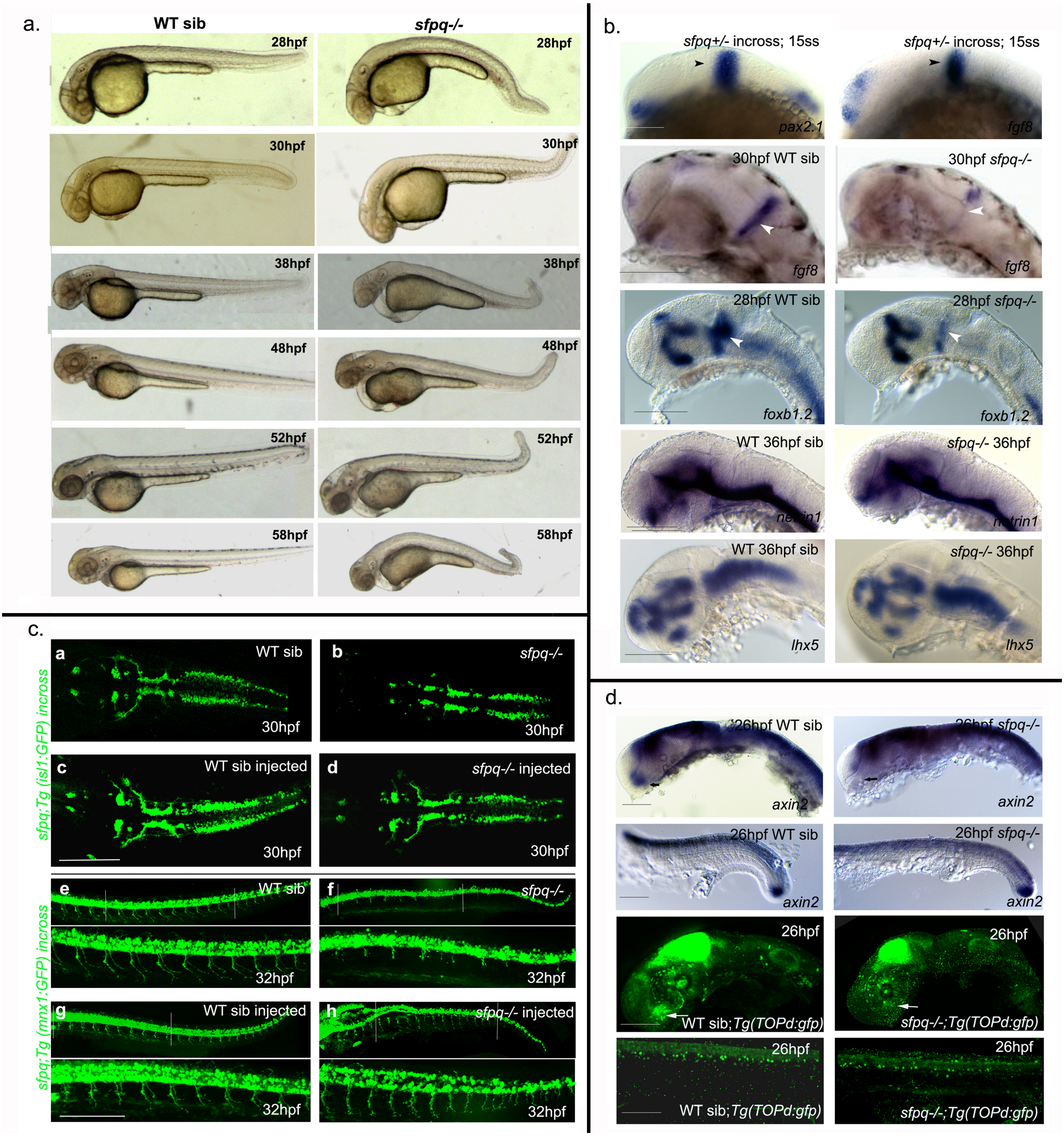

Suppl. Figure 2

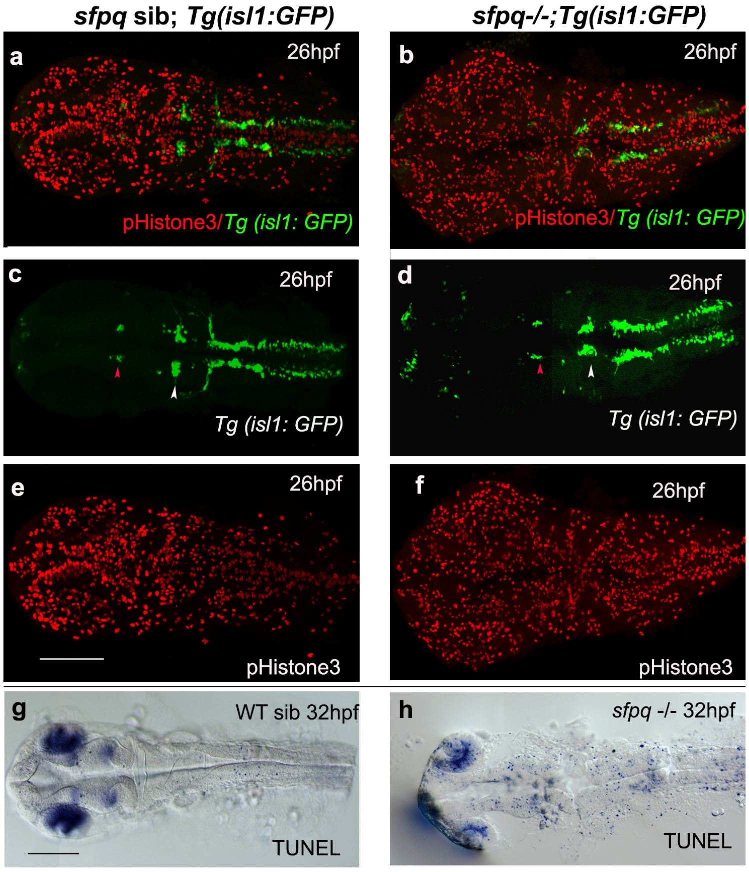

i

Count of pH3 stained cells in *sfpq;Tg(isl1:GFP)* embryos

Stage	Forebrain		Midbrain	Hindbrain			Total	
	Wtsib	*sfpq*	Wtsib	*sfpq*	Wtsib	*sfpq*	Wtsib	*sfpq*
**24hpf**	208	207	212	192	311	192	731	591
	205	117	182	104	214	133	601	354
**26hpf**	296	432	185	225	355	324	836	981
	178	338	144	185	198	340	520	863
	232	176	216	146	292	266	740	588
**48hpf**	234	429	329	321	360	254	923	1004
	207	364	262	459	263	501	732	1324
	214	304	186	466	146	427	546	1197
**56hpf**	325	198	201	169	239	239	765	606
	203	180	257	134	164	239	624	553
	193	174	269	132	192	246	654	553
p-value		**0.28**		**0.84**		**0.36**		**0.39**

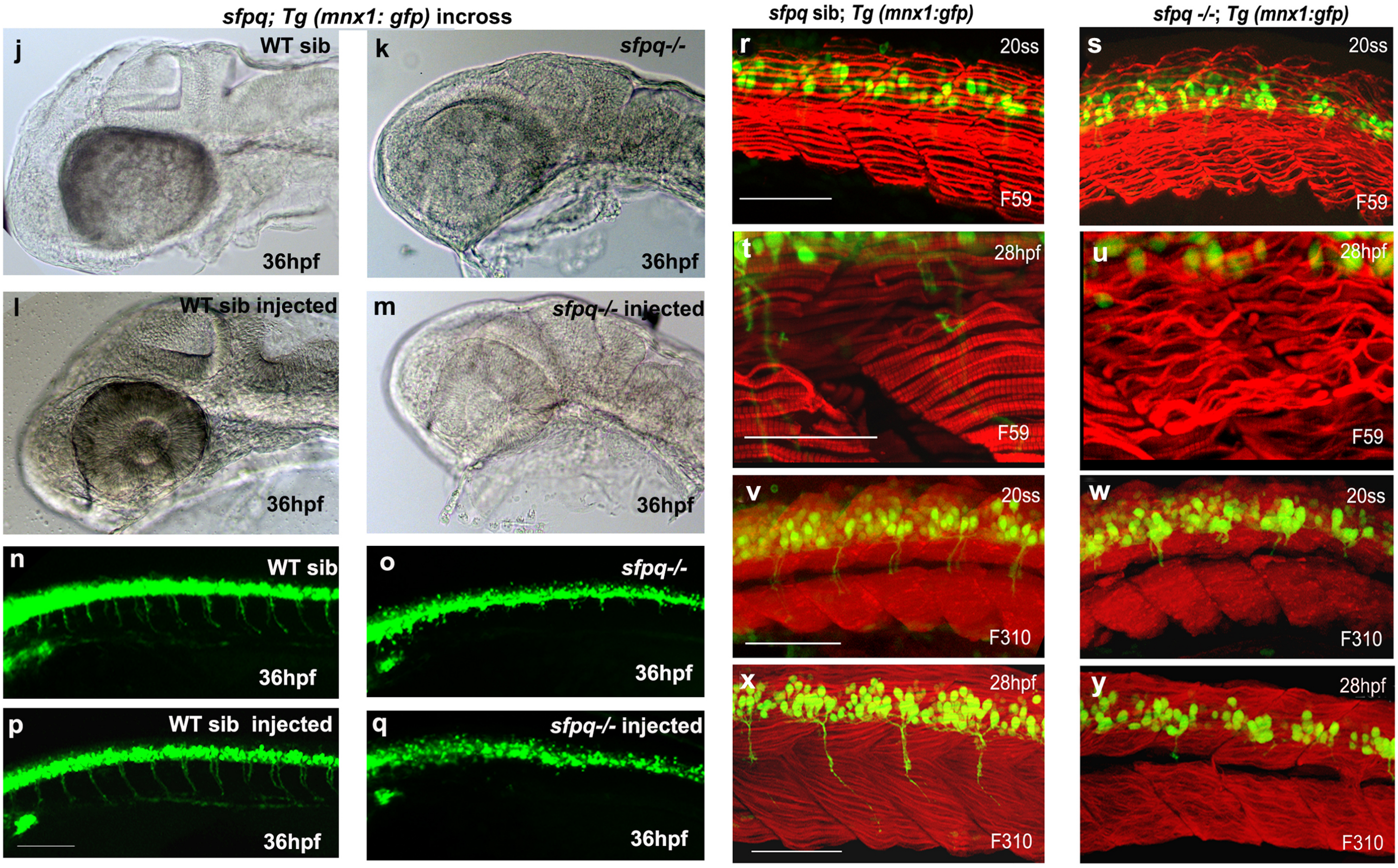

a.

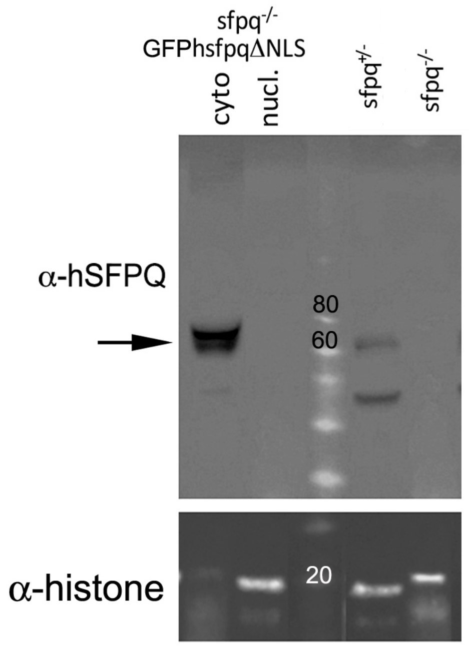

b.

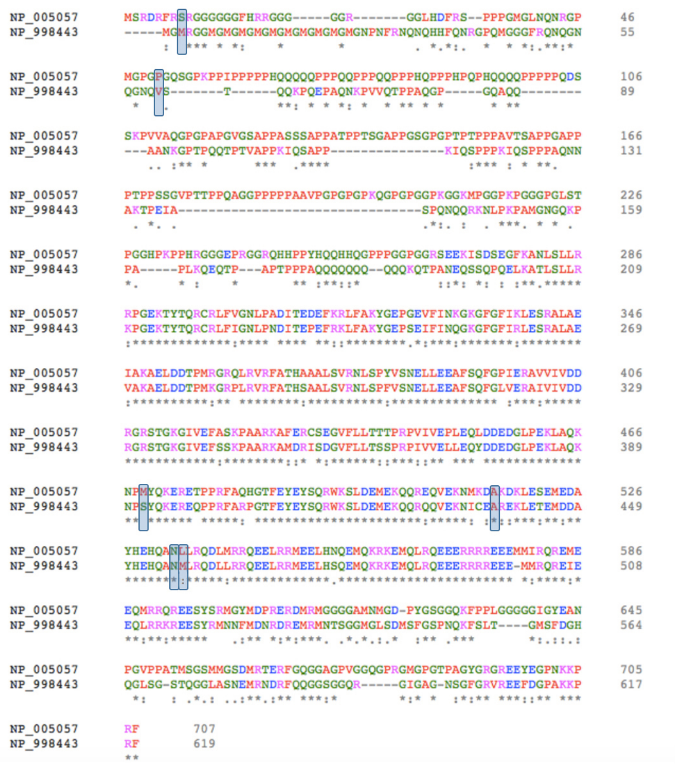

c.

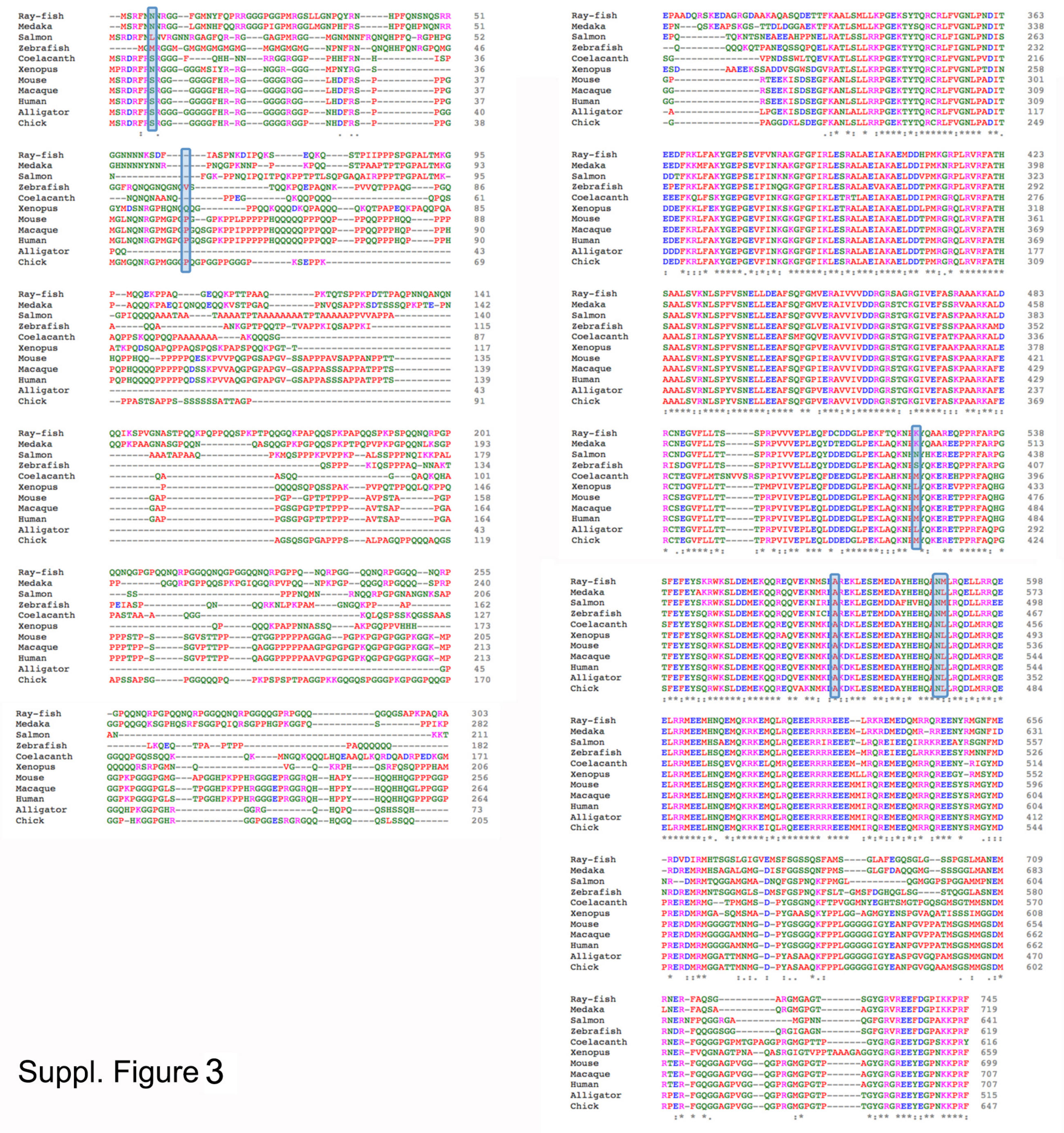

Suppl. Figure 3

Supplemental Table 1 and 2 relates to Figure 4**;** **Table 3** relates to Figure 5 and **Table 4** to Figure 6.

Supplemental Figure 1 (Related to Figure 1)

a. Temporal phenotypic progression in *sfpq* mutant
Lateral view of live WT siblings and *sfpq* mutant with anterior to the left at various developmental stages: 28hpf (n=31), 30hpf (n=47), 38hpf (n=21), 48hpf (n=19), 52hpf (n=41) and 58hpf (n=71), with 25% of the total (n) are homozygous mutants . Scale bar=100µm.
b. Normal overall brain patterning and induction of MHB in *sfpq* mutant
Lateral view of zebrafish brain (**a-l**) and spinal cord (**m,n,q,r**) with anterior to the left. Normal induction of MHB (black arrowhead) is observed in *sfpq* mutants with normal expression of *pax2.1* and *fgf8* at the MHB at 15ss (n=55; n=62). Expression of *fgf8* and *foxb1.2* in the MHB (white arrowhead) is greatly reduced in the *sfpq* mutants (n=10/40; n=5/21) by end of somitogenesis. No difference in the pattern of *netrin1* and *lhx5* expression was detected (n=9/36; n=6/25).
c. Wnt signaling in the sfpq mutant
The *axin2* and *topd gfp* expression was normal in the spinal cord and brain except for its expression (white arrow) in the hypothalamus of WT siblings that is absent in the *sfpq-/-* embryos (n=11/42; n=7/35) showing the absence of Wnt activity in the region. Scale bar=100µm.
c. Rescue of mutant phentotpye with *sfpq* RNA injection
Dorsal view (**a-d**) of zebrafish brain at 30hpf and lateral view (**e-h**) of zebrafish spinal cord at 32hpf with anterior to the left. The embryos from both the *sfpq;Tg(isl1:gfp)* incross (n=51) and the *sfpq;Tg(mnx1:gfp)* incross (n=76) injected with *sfpq* mRNA appeared almost normal compared to its control WT siblings with the rescue of axonogenesis defect and the kinky tail that is observed in the non-injected *sfpq* mutants. Scale bar=100µm.

Supplemental Figure 2 (Related to Figure 2)

Cell proliferation and apoptosis in *sfpq* mutant
Dorsal view of zebrafish brain at 26hpf (**a-f**) and 30hpf (**g-h**) with anterior to the left. **a-f.** Cell proliferation in *sfpq* mutant brain (n=11) analysed using *gfp* expression in *sfpq;Tg:isl1 gfp* as a landmark to delineate the domains: nIII neurons (red arrowhead) as posterior limit of the forebrain and the anterior limit of the midbrain; nV neurons (white arrowhead) as the posterior limit of the midbrain and the anterior limit of the hindbrain. Cell proliferation in the tail of *sfpq* mutant was found in par with that of its WT siblings (data not shown). **g,h.** Increased apoptotis is observed throughout the the *sfpq* brain (n=4) compared to siblings (n=12). **i**. Proliferation quantified using phosphor-Histone3 staining, counted in different brain regions of *sfpq* mutant and siblings. No significant difference was observed in cell count (Student's t-test, p>0.05). Increased cell proliferation observed in some (n=5 of the 11 counted) may be due to cells getting arrested at G2/M phase of cell cycle.
***sfpq* initial phenotype is independent of cell death**
j-q. p53 knockdown in *sfpq;Tg*(*mnx1: gfp*) embryos. Lateral view of zebrafish brain (**j-m**) and spinal cord (**n-q**) at 36hpf with anterior to the left. The p53 MO injected $sfpq^{-/-}$ embryos (**k, o.** n=3) show similar MHB phenotype and axonogenesis defect as the non-injected mutants (**m, q.** n=5), compared to injected (**l, p.** n=14) and non-injected siblings

(**j, n.** n=11). Scale bar=100µm.

Muscle formation in *sfpq* mutant

r-y. Lateral view of zebrafish muscle, anterior to the left. **r-u**. Immunostaining for slow muscle with F59 antibody shows proper somatic boundaries and differentiated slow muscle fibres and sarcomeres in the *sfpq* mutant (20ss: n=6/25; 28hpf**:** n=9/39). The wavy appearance of slow muscle fibres in the mutant at later stages compared to its WT siblings (**c-f**), likely due to inactivity. **v-y.** Immunostaining for fast muscle with F310 antibody shows the integrity of the fast muscle in *sfpq* mutant (20ss: n=5/22; 28hpf: n=6/24) as normal as in its WT siblings. Scale bar=100µm.

Supplemental Figure 3 (Related to Figure 6 and 7)

a. Western blots, incubated with either anti-histone H3 or anti-SFPQ, of total extracts from zebrafish siblings and null mutant (right lanes) and of cytoplasmic or nuclear extracts (left lanes) from null mutants injected with GFP-tagged human SFPQ carrying deletion of its C-terminal NLS. Lower band suggests second shorter isoform in zebrafish or cleaved product.
b. Comparison between human (NP_0105057) and zebrafish (NP_998443) protein sequences. Boxes highlight the amino-acids targeted in the human variants chosen for our blind tests.
c. Comparison across species from fish to mammals. Boxes highlight the amino-acids targeted in the human variants chosen for our blind tests.
