# Supplementary material for: Non-nuclear Pool of Splicing Factor SFPQ Regulates Axonal Transcripts Required for Normal Motor Development
Source: Neuron. 2017 Apr 19;94(2):322–336.e5. doi: 10.1016/j.neuron.2017.03.026 (PMC5405110; doi:10.1016/j.neuron.2017.03.026)
Supplement: Document S2. Article plus Supplemental Information [file mmc5.pdf]

Neuron

Article

Non-nuclear Pool of Splicing Factor SFPQ Regulates Axonal Transcripts Required for Normal Motor Development

Highlights

- SFPQ splicing factor is present in motor axons
- Non-nuclear SFPQ is able to drive axon maturation and connectivity
- Loss of axonal SFPQ affects axonal morphology
- Coiled-coil domain of the protein is important for non-nuclear localization

Authors

Swapna Thomas-Jinu,
Patricia M. Gordon, Triona Fielding, ...,
Ian P. Blair, Christopher E. Shaw,
Corinne Houart

Correspondence

corinne.houart@kcl.ac.uk

In Brief

Thomas-Jinu et al. demonstrate that a non-nuclear pool of the splicing factor SFPQ is necessary for normal motor development, through local mRNA maintenance or processing. SFPQ's coiled-coil domain is required for axonal localization. Their findings may have an important impact in understanding human motor neuron disorders.

CrossMark
Thomas-Jinu et al., 2017, Neuron *94*, 322–336
April 19, 2017 © 2017 The Author(s). Published by Elsevier Inc.
http://dx.doi.org/10.1016/j.neuron.2017.03.026

CellPress

Neuron
Article

Non-nuclear Pool of Splicing Factor SFPQ Regulates Axonal Transcripts Required for Normal Motor Development

Swapna Thomas-Jinu,[1] Patricia M. Gordon,[1] Triona Fielding,[1] Richard Taylor,[1] Bradley N. Smith,[2] Victoria Snowden,[1] Eric Blanc,[1] Caroline Vance,[2] Simon Topp,[2] Chun-Hao Wong,[2] Holger Bielen,[1] Kelly L. Williams,[4] Emily P. McCann,[4] Garth A. Nicholson,[4,5] Alejandro Pan-Vazquez,[1] Archa H. Fox,[6,7,8] Charles S. Bond,[9] William S. Talbot,[3] Ian P. Blair,[4] Christopher E. Shaw,[2] and Corinne Houart[1,10,*]
[1]Centre for Developmental Neurobiology and MRC CNDD, IoPPN, Guy's Campus, King's College London, London SE1 1UL, UK
[2]Department of Basic and Clinical Neuroscience, Institute of Psychiatry, Psychology, and Neuroscience, King's College London, London SE5 8AF, UK
[3]Department of Developmental Biology, Stanford University School of Medicine, Stanford, CA 94305, USA
[4]Department of Biomedical Sciences, Faculty of Medicine and Health Sciences, Macquarie University, Sydney, NSW 2109, Australia
[5]ANZAC Research Institute, University of Sydney, Concord Hospital, Sydney, NSW 2139, Australia
[6]School of Anatomy, Physiology, and Human Biology, University of Western Australia, Crawley, WA 6009, Australia
[7]Harry Perkins Institute for Medical Research, QEII Medical Centre, Nedlands, WA 6009, Australia
[8]Centre for Medical Research
[9]School of Chemistry and Biochemistry
University of Western Australia, Crawley, WA 6009, Australia
[10]Lead Contact
*Correspondence: corinne.houart@kcl.ac.uk
http://dx.doi.org/10.1016/j.neuron.2017.03.026

SUMMARY

Recent progress revealed the complexity of RNA processing and its association to human disorders. Here, we unveil a new facet of this complexity. Complete loss of function of the ubiquitous splicing factor SFPQ affects zebrafish motoneuron differentiation cell autonomously. In addition to its nuclear localization, the protein unexpectedly localizes to motor axons. The cytosolic version of SFPQ abolishes motor axonal defects, rescuing key transcripts, and restores motility in the paralyzed sfpq null mutants, indicating a non-nuclear processing role in motor axons. Novel variants affecting the conserved coiled-coil domain, so far exclusively found in fALS exomes, specifically affect the ability of SFPQ to localize in axons. They broadly rescue morphology and motility in the zebrafish mutant, but alter motor axon morphology, demonstrating functional requirement for axonal SFPQ. Altogether, we uncover the axonal function of the splicing factor SFPQ in motor development and highlight the importance of the coiled-coil domain in this process.

INTRODUCTION

Post-transcriptional regulation of gene expression plays a fundamental role in the temporal and spatial modulation of development and has been recently found at the core of many disease mechanisms. In recent years, much progress has been made in unraveling the players in this process, revealing a daunting complexity in the mechanisms of RNA processing and transport. This complexity is not only created by the many players involved and their intricate interactions in multiple aspects of RNA regulation (transcription termination, non-coding RNA interaction, RNA processing, and transport), but is also generated by the complex role of long non-coding RNAs (Derrien et al., 2012; Mercer and Mattick, 2013; Okazaki et al., 2002).

Our zebrafish forward-genetic screen for embryos displaying abnormalities in neural development led to the isolation of a null mutation in the gene encoding splicing factor proline/glutamine-rich (SFPQ, a.k.a. PSF). SFPQ is a multifunctional protein (Shav-Tal and Zipori, 2002), known to be involved in pre-mRNA splicing (Rosonina et al., 2005), transcriptional repression, and DNA repair, originally isolated from the spliceosome. It is associated with nuclear paraspeckles (Fox et al., 2002) involved in nuclear retention of defective RNAs into subnuclear sequestration of SFPQ complexes (Barry et al., 2014; Johnson, 2012) and with small cytoplasmic RNA granules, the function of which is not yet understood (Kanai et al., 2004).

The structure of SFPQ is conserved and closely related to NONO and PSPC1 proteins, with which it forms heterodimers. They all contain tandem RNA recognition motif domains, a family-specific NOPS (Nono, Pspc1, and Sfpq) domain and a coiled-coil region (Passon et al., 2012). SFPQ additionally contains a DNA-binding domain (DBD) and an N-terminal proline/glutamine-rich low-complexity region (Lee et al., 2015; Patton et al., 1993; Peng et al., 2002; Shav-Tal and Zipori, 2002; Urban et al., 2000). Its RNA-binding activity has been implicated in a variety of cellular processes (Chanas-Sacré et al., 1999; Figueroa et al., 2009; Lukong et al., 2009; Rosonina et al., 2005;

 © 2017 The Author(s). Published by Elsevier Inc.
This is an open access article under the CC BY license (http://creativecommons.org/licenses/by/4.0/).

Shav-Tal et al., 2001; Shav-Tal and Zipori, 2002; Sutherland et al., 2005). Of interest for our study, SFPQ has recently been shown to regulate signal-induced alternative splicing (Ray et al., 2011) and to be required, directly or indirectly, for transport of an RNA regulon that promotes sensory axon viability (Cosker et al., 2016). SFPQ is present in cytoplasmic aggregates in brains of patients with a variety of neurodegenerative disorders and is mis-regulated in autism and dyslexia (Chang et al., 2015; Ke et al., 2012; Kubota et al., 2010; Stamova et al., 2013; Tapia-Páez et al., 2008). The human *SFPQ* gene lies in a region on chromosome 1p34-p36 linked to speech disorders and language impairment (Grigorenko et al., 2001; Kubota et al., 2010; Miscimarra et al., 2007; Rabin et al., 1993; Tzenova et al., 2004).

Phenotypic characterization of our zebrafish *sfpq* homozygous mutants revealed a restricted set of defects, unexpected for a ubiquitously expressed multifunctional protein. The CNS was prominently affected, showing brain boundary and axonal defects associated with absence of motility. We assessed the specificity of SFPQ functional targets by microarray RNA profiling analysis, comparing the transcriptome of the *sfpq* homozygous mutants to its wild-type and heterozygous siblings at the earliest stage at which the phenotype is robustly recognizable. An unexpectedly narrow set of transcripts was affected by the loss of SFPQ, with a specific enrichment in alternatively spliced neuronal and synaptic transcripts. The use of a tagged version of the protein revealed that SFPQ is abundant in the axons of a subset of neuronal populations including all motor neurons (MNs), an observation confirmed by antibody staining of endogenous SFPQ. We demonstrated that these axons also contain intron-retaining transcripts and spliceosome core components, suggesting a possible involvement in non-canonical cytoplasmic RNA processing. We demonstrate that cytoplasmic SFPQ is functionally relevant to MN differentiation, showing that a non-nuclear version of the protein is able to restore a specific set of neuronal transcripts and rescue the axonal and motility defects in the zebrafish null mutant. The severe motor phenotype of the zebrafish null mutant and presence of SFPQ in aggregates in human degenerative disorders encouraged us to screen ALS patients for *SFPQ* mutations. From these patients, we identified two novel *SFPQ* missense variants affecting the coiled-coil domain of the protein and absent from ~66,000 normal human exomes. In double-blinded zebrafish null mutant rescue experiments, the ALS-linked SFPQ variants uniquely led to motor axon morphological abnormalities in the rescued mutants and showed a much-reduced cytoplasmic localization. These findings uncover the importance of the non-nuclear axonal function of SFPQ protein in motor development and indicate the importance of the coiled-coil domain in this process.

RESULTS

Loss of Zebrafish sfpq Affects Development of Brain Boundaries and Motor Function

A recessive zebrafish mutant identified during a small-scale ENU mutagenesis screen was selected for its early brain boundary defects and was named *coma* due to its vegetative state and its later comma-shaped body (Figure S1A). Preceding any morphologically visible defect, homozygous mutants fail to twitch their tail at 15–16 somite stage (ss). By 24 hr post-fertilization (hpf), all homozygous mutant larvae fail to undergo the morphogenetic changes required to form the posterior mesencephalic wall and a mature isthmic organizer (Figures 1A and 1B), and 22% have asymmetric ectopic mesencephalic neuroepithelial folds (Figures 1C and 1D). The expression of midbrain-hindbrain boundary (MHB) markers, *pax2.1* and *fgf8*, is normal at early stages, but reduced or lost at later stages, indicating that the midhindbrain boundary is established, but not maintained (Figures 1E, 1F, and S1B). The mutant also lacks rhombomere boundaries, failing to express early boundary markers such as *rfng* (Figures 1G and 1H), and undergoes ectopic neurogenesis in boundary regions (Figures 1I–1L). These boundary defects are neither due to a general loss of apical-basal polarity, nor to specific cell death in these areas (Figures S2E and S2H).

Most other aspects of CNS patterning appear normal in the mutant apart from a loss of Wnt signaling activity observed in the hypothalamus (Figure S1D). No significant difference in cell proliferation was found during the two first days of development (Figures S2A–S2F and S2I). All mutant embryos develop further defects such as lack of heart looping, pericardial edema, short curved body, and kinky tail (Figure S1A); never acquire motility (Movie S1); and die by 4 days post-fertilization (dpf).

The genetic characterization of *coma*$^{-/-}$ by fine mapping and candidate gene analysis indicated that the genetic lesion was in *sfpq*. This was confirmed by complementation test with an existing allele of the gene (*whitesnake*, *wis*^{tr241}) (Lowery et al., 2007). The point mutation in *coma* was then identified by sequencing as a C-to-T transition at +604 position from the ATG start codon, changing CAA to TAA (glutamine [Q] at aa. 203 to STOP codon) in the proline (P)/glutamine (Q)-rich domain of the protein (Figure 1M). The rescue of the mutant phenotype by microinjection of the zebrafish or human *sfpq* full-length mRNA (Figures S1C and 7) establishes that *coma* is a novel null allele of the gene *sfpq* (named *sfpq*^{kg41}) located at 56.2 cM on zebrafish chromosome 19 and that the protein has a conserved function in zebrafish and human.

Sfpq Is Cell-Autonomously Required for Motor Axon Development

As the earliest observable *sfpq*^{kg41} defect is its lack of early motility at 16 ss, we investigated its neuronal differentiation. From 24 hpf onward, homozygous *sfpq*$^{-/-}$ embryos show defects in axonal tracts in discrete areas of CNS and peripheral nervous system (PNS), with lack of posterior and supra-optic commissures and reduced inter-rhombomeric commissural axons (Figures 2A–2D). These also show defects in the axonal projections of both cranial (Figures 2E and 2F) and spinal cord MNs (Figures 2G–2P).

The *sfpq*^{kg41} anterior spinal motor axons do occasionally manage to form and exit normally the spinal cord, neuronal behavior more readily observed in the *Tg(Xla.Tubb2b:Hsa.MAPT-GFP)*^{zc1} background, expressing a GFP-tagged microtubule-associated Tau protein in neurons, indicating that Tau overexpression has some beneficial effect in sfpq^{kg41} neurons. In this transgenic background, around 15% of homozygous mutants form anterior axons with neuro-muscular junctions (NMJs) and

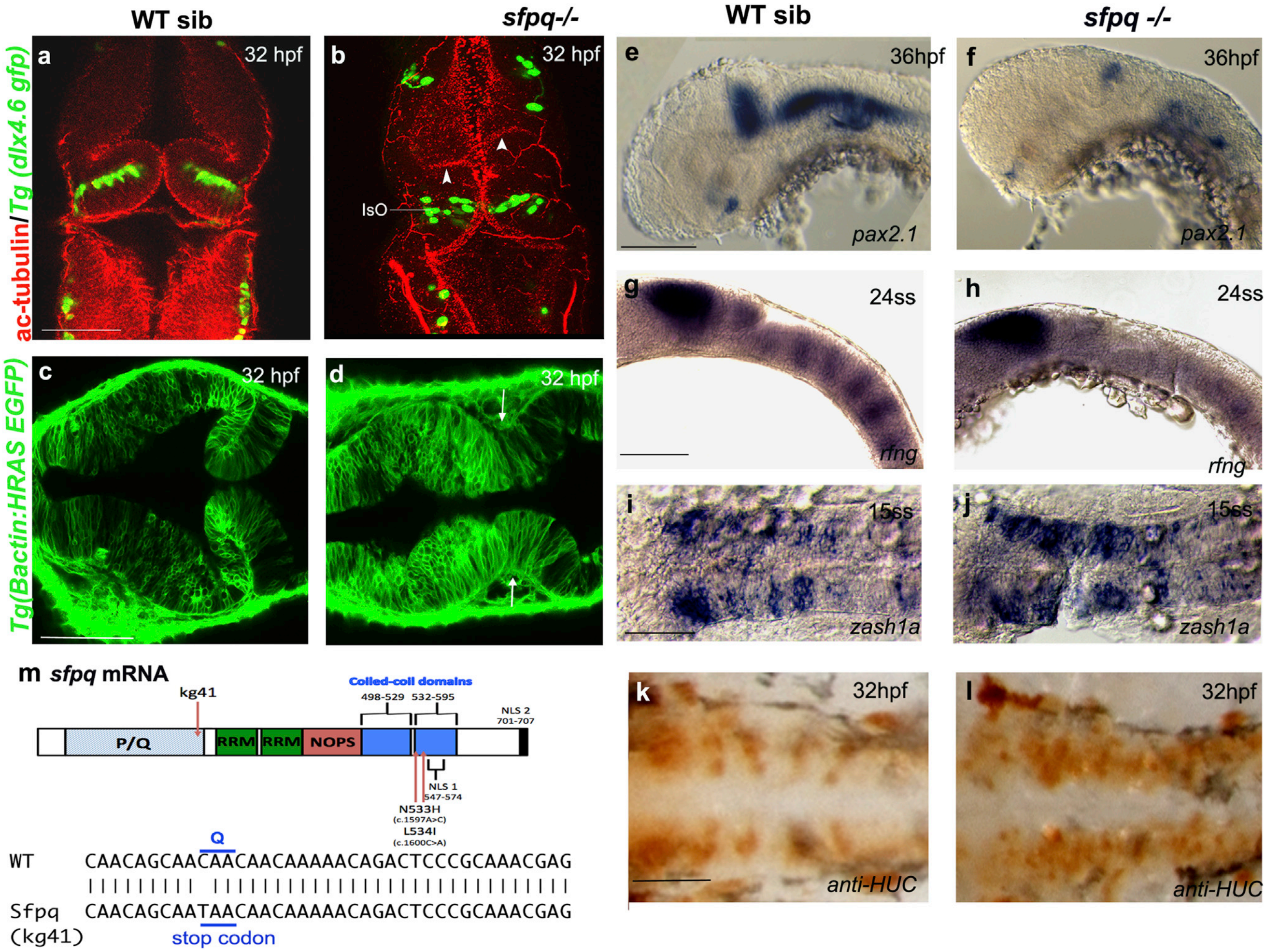

Figure 1. SFPQ Is Required for Brain Boundaries

Dorsal (A–D and I–L) and lateral (E–H) views of 32 hpf zebrafish brain with anterior to the top (A and B) or left (C–L).

(A and B) Immunostaining of *sfpq; Tg(dlx4.6 GFP)* embryos. Anti-acetylated tubulin staining (red) reveals asymmetrical folds in the midbrain (white arrowheads) and thickening of the isthmic organizer (IsO) in coma mutant (B; n = 8) compared to its wild-type sibling (A; n = 24). GFP staining (green) reveals disorganized neuronal distribution in the cerebellum.

(C and D) Dorsal views, anterior to the top, of sibling (C) and mutant (D) *sfpq;Tg(βactin: HRAS gfp)* embryos showing failure of morphological thinning of the isthmus (white arrows in D, n = 9/9) in the homozygous mutant.

(E and F) Expression of *pax2.1* in the MHB, greatly reduced in all *sfpq* mutants (F; n = 10) compared to siblings (E; n = 30) at 36 hpf.

(G and H) Expression of boundary marker, *rfng*, in siblings (G; n = 21) and mutant (H; n = 7, reduced or absent) at 24 ss.

(I and J) Expression of *zash1a* in the hindbrain at 15 ss, in siblings (I; n = 19) and mutant (J; n = 6).

(K and L) HuC staining at 32 hpf, in siblings (K; n = 17) and mutant (L; n = 5). Scale bar, 100 μm.

(M) Schematic of the *sfpq* gene and the zebrafish and human mutations described in this report and, below, the sequence altered in the zebrafish *coma* mutant. PQ, proline (P) glutamine (Q) rich; RRM, RNA recognition motif; NOPS, NONA/paraspeckle domain; NLS, nuclear localization signals.

can generate some movements (Movie S2). In all other backgrounds, most anterior MNs do not progress, stalling at the horizontal myoseptum and either stop there or branch excessively with poor progression along the somitic mass (Figures 2G–2N). Immunostaining with α-bungarotoxin (α-btxn; data not shown) and pre-synaptic SV2 shows normal pre- and post-synaptic pre-patterning in the mutant (Figures 2O and 2P; data not shown). However, the mutants never form mature NMJs. The motor axon defect of the mutant is not due to cell death, as healthy MN cell bodies are found until day 4 (GFP^+ in Figures 2G–2N) and the mutants lacking p53 (thereby devoid of cell death; Sakhi et al., 1994) still exhibit severe axogenesis defects (Figures S2J–S2Q).

Somitic muscle differentiation occurs normally in the *sfpq* mutant, with typical V-shaped somitic boundaries, proper differentiation of both slow and fast muscle, and formation of sarcomeres (Figures S2R–S2Y). The absence of axons in the vast majority of MNs, without initial defects in muscle differentiation, indicates that the primary defect is in the MNs.

Mosaic experiments (Figure 3) further demonstrate that *sfpq* is cell-autonomously required in neurons for their maturation.

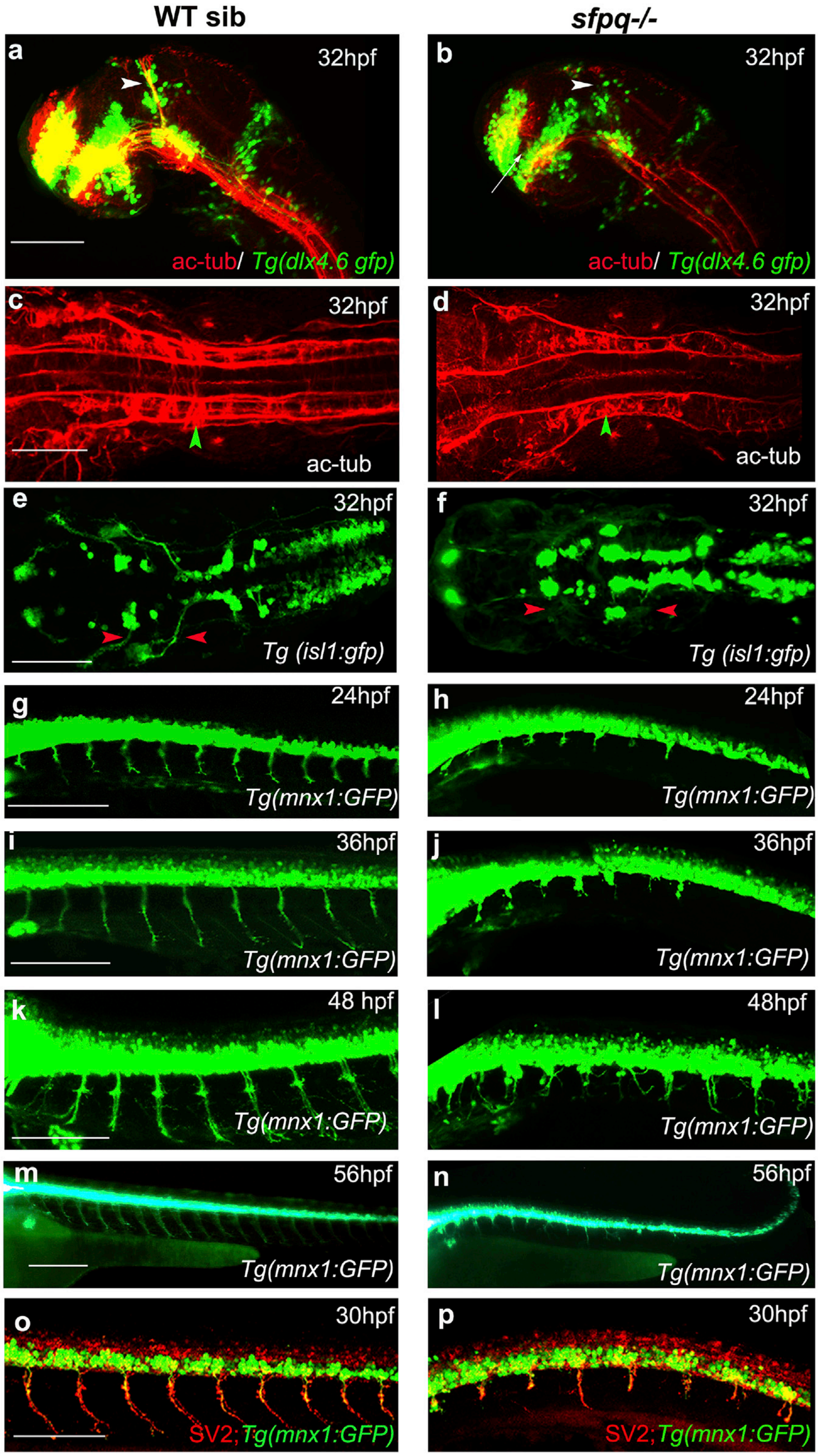

Figure 2. Axonogenesis Is Affected in *sfpq*$^{-/-}$ Embryos

Lateral (A, B, and G–P) and dorsal (C–F) views, anterior to the left, of zebrafish brain at 32 hpf (A and B) and spinal cord at 24–56 hpf.

(A and B) *sfpq;Tg(dlx4.6:gfp)* embryos. Lack of supra-optic commissure (white arrow) and posterior commissure (white arrowhead) in coma (n = 8/32) is revealed by acetylated tubulin staining.

(C and D) Dorsal view, anterior to the left of acetylated tubulin staining showing hindbrain disorganized axonal tracks in *sfpq* mutant (D; green arrowhead, n = 8) compared to siblings (C; n = 26).

(E and F) Dorsal view, anterior to the left of GFP^{+} motor neurons in *sfpq; Tg(isl1:gfp)* siblings (E) and mutant (F), showing cranial motor neuronal clusters lacking axonal projections in the mutant (red arrowhead, n = 7).

(G–N) Lateral view, anterior to the left, of confocal live imaging, showing temporal defect in axonogenesis in the majority of spinal motor neurons in a mutant (H, J, L, and N; n = 14) compared to a sibling (G, I, K, and M; n = 42) in the *Tg(mnx1: gfp)* background.

(O and P) Lateral view, anterior to the left, of SV2 antibody staining of siblings (O) and *sfpq* null mutant (P), showing pre-synaptic protein in the few axons formed in *sfpq* mutants (n = 9). Scale bar, 100 μm.

Cell transplantations in spinal cord territory of the neural plate at ~70% epiboly show that *sfpq*$^{-/-}$ MNs transplanted into wild-type embryos fail to show motor axon projections in 30 hpf host embryos (Figures 3G–3L and 3N; n = 8). Transplanted *sfpq*$^{-/-}$ ventral neuro-progenitors do not show significant change in motor to interneuron ratio (Figure 3M; isl1/isl2a staining; data not shown). Conversely, donor wild-type MNs form functional axons (Figures 3C, 3D, and 3N; n = 7), eliciting muscle twitching, in 30 hpf *sfpq*$^{-/-}$ mutant embryos. The donor neurons show some expected late axonal deformation in the strongly affected 3 dpf mutants (Figures 3E and 3F).

SFPQ Regulates a Specialized Set of Transcripts

To uncover the molecular complexity driving loss of motility in *sfpq*^{kg41}, we analyzed the mutant transcriptome at onset of the phenotype. Nimblegen microarray transcriptome analysis, on triplicate samples of total RNA extracted from 50 *sfpq*$^{-/-}$*;Tg(mnx1:GFP)* and sibling embryos at the 22 ss, shows 571 of the 38,489 probes differentially expressed (99% confidence level). The vast majority of these transcripts (566/571) were downregulated in the homozygous *sfpq* mutant, and only five were overexpressed (Figure 4A). Validation of the microarray data was achieved by qPCR on randomly picked downregulated transcripts (Figure 4B).

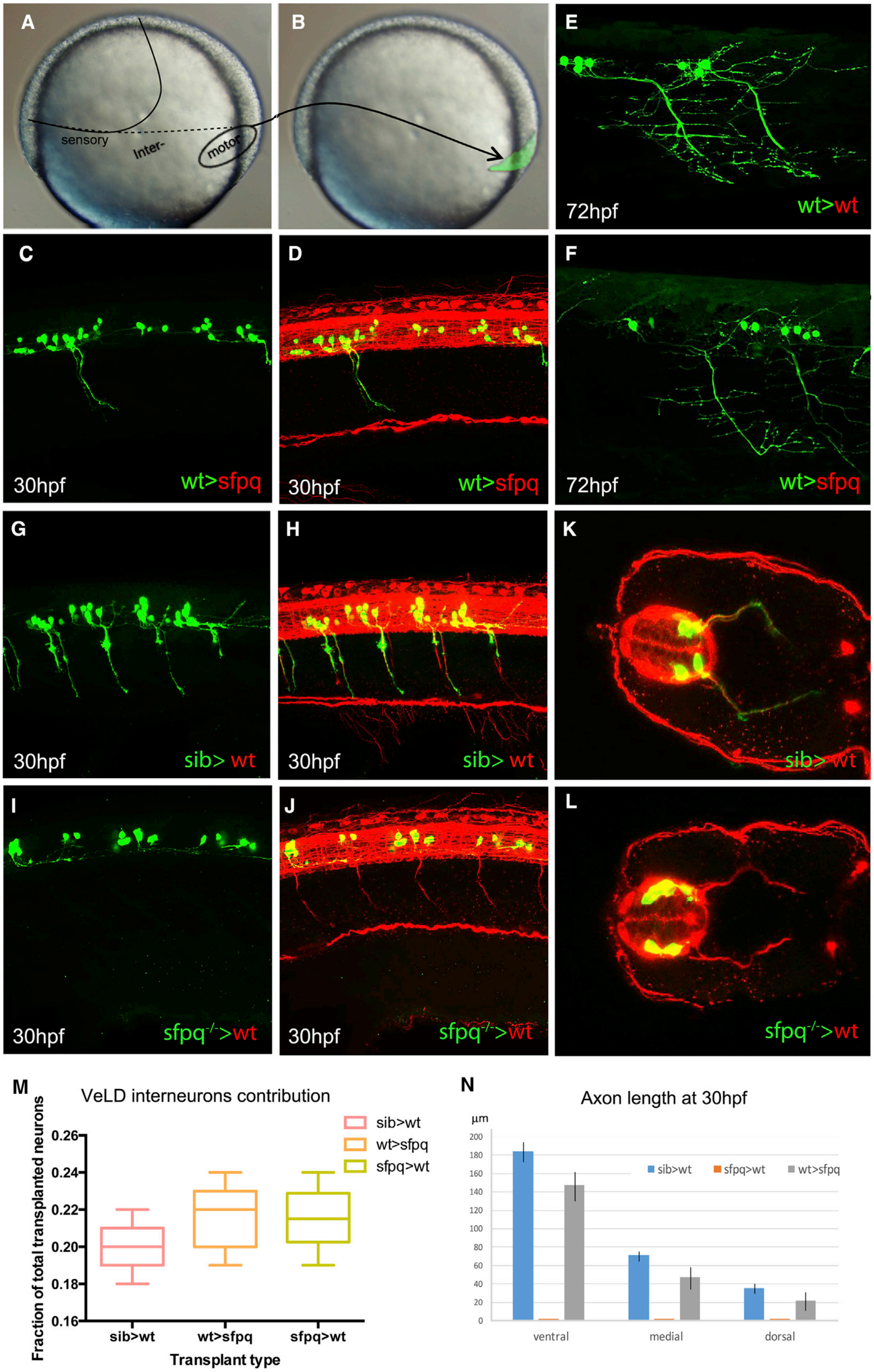

(legend on next page)

ArrayStar analysis with the ZFIN gene annotation file provided annotation with gene ontology (GO) terms for only 8% (3,043 probes) of the whole chip and 7% (41 probes) of the SFPQ differentially expressed set (DES). With the customized gene annotation file generated (see STAR Methods), 76% (29,364 probes) of the chip was mapped to gene identities and 70% (26,798 probes) provided GO terms for the total set on the array. Of the DES, 81% (461 of the 571 probes) were mapped to gene symbols (Table S1) and 74% (424 probes) of these are associated with GO terms (Table S2). Of the five upregulated in the DES, only three are mapped to gene symbols. Out of the 26,798 total annotated probes, a total of 16,745 whole chip were annotated with GO terms. In total, 14 of the 59 GO terms at level 2 of the GO term hierarchy were enriched in the annotated array ($p < 0.05$ after multiple testing correction; Figure 4C). Considering all levels of hierarchy, only 192 of the 8,624 GO terms represented in the total annotated array set were found enriched in the DES. These identified an unambiguously specific enrichment for molecules involved in cell adhesion, cell junctions, neuronal/synaptic structures, glutamate, and Wnt signaling (Figure 4C; Table S2). Moreover, the three upregulated transcripts (*pard3*, *arvcfb*, and *dgat1*) are involved in cell adhesion (Kausalya et al., 2004), cell polarity (Shi et al., 2003), adherens junction complexes (Gladden et al., 2010; Mariner et al., 2000), and dendritic spine morphogenesis (Zhang and Macara, 2006). Finally, 21% of the SFPQ transcriptome code for proteins identified as part of the synaptic proteome. The mutants undergo a substantial downregulation of neuronal-specific transcripts (genes listed in Figure S4) despite an increase in neuronal populations (ectopic neurogenesis in rhombomeric boundaries; Figure 1). Finally, when compared with the whole-genome transcriptome, the differentially expressed set is substantially enriched for alternatively spliced transcripts and transcripts containing multiple, longer than average introns (Figure 4D; data not shown). The enrichment in transcripts with long introns is reminiscent of the findings showing a preference of TDP43 and FUS for binding to transcripts with long first introns (Lagier-Tourenne et al., 2012).

Non-nuclear Localization of Splicing Factors and Introns in Developing Neurons

Using antibodies, we assessed SFPQ protein localization in wild-type and maternal proteins in the mutant. In line with the reported role of SFPQ in many nuclear functions, we found robust nuclear staining throughout zebrafish development, with systematic enhanced expression in nuclei of post-mitotic neurons throughout development (Figures 5A and 5D–5F). Moreover, protein detection was unexpectedly found in variable levels in motor axons (arrows in Figures 5B, 5D, and 5F) and discrete axonal and dendritic populations throughout the CNS. Whole mount and western blot show that no detectable SFPQ protein was found in the mutant after the 12 ss (data not shown), showing that maternal contribution stops by that stage.

In order to look at the dynamics of protein localization in live zebrafish, we designed a functionally active GFP-tagged version of zebrafish *sfpq*. Confirming the antibody results, we have found that injection of z-SFPQ-GFP DNA leads to nuclear GFP expression, including bright nuclear foci, in all cell types and GFP detection in neurites of some neuronal populations, including MNs (Figures 5E and 5F).

Together with the downregulation of neuronal and synaptic transcripts in the $sfpq^{-/-}$ zebrafish mutant, this supports the possibility of a non-nuclear function for SFPQ, related either to the transport of mRNA and/or pre-RNA, or to non-nuclear RNA processing events. To begin to discriminate between these possibilities, we assessed whether SFPQ-regulated transcripts were present in any unprocessed form in wild-type neurites. We chose to screen for the first alternative intron of a series of SFPQ-dependent neuronal transcripts. Introns were detected in neurites of wild-type embryos for 11 of 36 neuronal transcripts tested (Figures 5G–5M; Table S3; at least 20 embryos per probe), but undetectable in homozygous sfpq mutants, suggesting that unprocessed transcripts may be abundant in specific neuronal populations and degraded, or not produced, in *sfpq* mutants. The axonal presence of intronic sequences is coincidental with detection of the core spliceosome protein snRNP70 (U1 snRNP), expressed in neurites across the whole of the CNS, including motor axons (Figures 5N and 5O; n = 32).

Non-nuclear SFPQ Rescues the Motor Axon Defect of the sfpq Mutant

To assess whether the presence of SFPQ in axons is functionally relevant to MN function, we tested whether a cytoplasmic form of the protein was able to rescue the neuronal defect in the mutant. We made use of the human truncated protein lacking the C-terminal non-canonical nuclear localization signal (NLS), previously shown to prevent SFPQ nuclear import without affecting the structure and functional domains (Dye and Patton, 2001). We found that injection of the RNA coding for this cytoplasmic form is able to restore both motor axon growth and movement in the homozygous *sfpq* mutant (Figures 6E–6L; Movie S3; n = 37 homozygous embryos). Deletion of a second putative minor NLS sequence sitting in the coiled-coil domain (see Figure 1E) affected the protein structure and function (Lee et al., 2015; data not shown). The motor rescue can't be

Figure 3. Sfpq Is Required Cell Autonomously for Motor Axon Development

Homotopic transplantation at 70%–80% epiboly of $sfpq^{-/-}$; *Tg(mnx1:GFP)* and sibling *Tg(mnx1:GFP)* ventral spinal cord progenitors into wild-type or $sfpq^{-/-}$ hosts.

(A and B) Schematic of the transplants from donor (A) to host (B) embryo.

(C, D, and G–L) Lateral view, anterior to the left (C, D, and G–J) or transverse (K and L) of 30 hpf zebrafish transplanted trunks, showing the transplanted mnx1+ neurons in green and all axons in red (acetylated tubulin staining).

(E and F) Lateral view, anterior left of transplanted wild-type MNs (green) in early larvae (3 dpf) in wild-type (E) or $sfpq^{-/-}$ (F) hosts.

(M) Quantification of VeLD interneurons in transplanted clones at 30 hpf (value is number of interneurons/total number of transplanted neurons, number of embryos in box).

(N) Quantification of axonal lengths of transplanted neurons at 30 hpf. Scale bar, 100 μm.

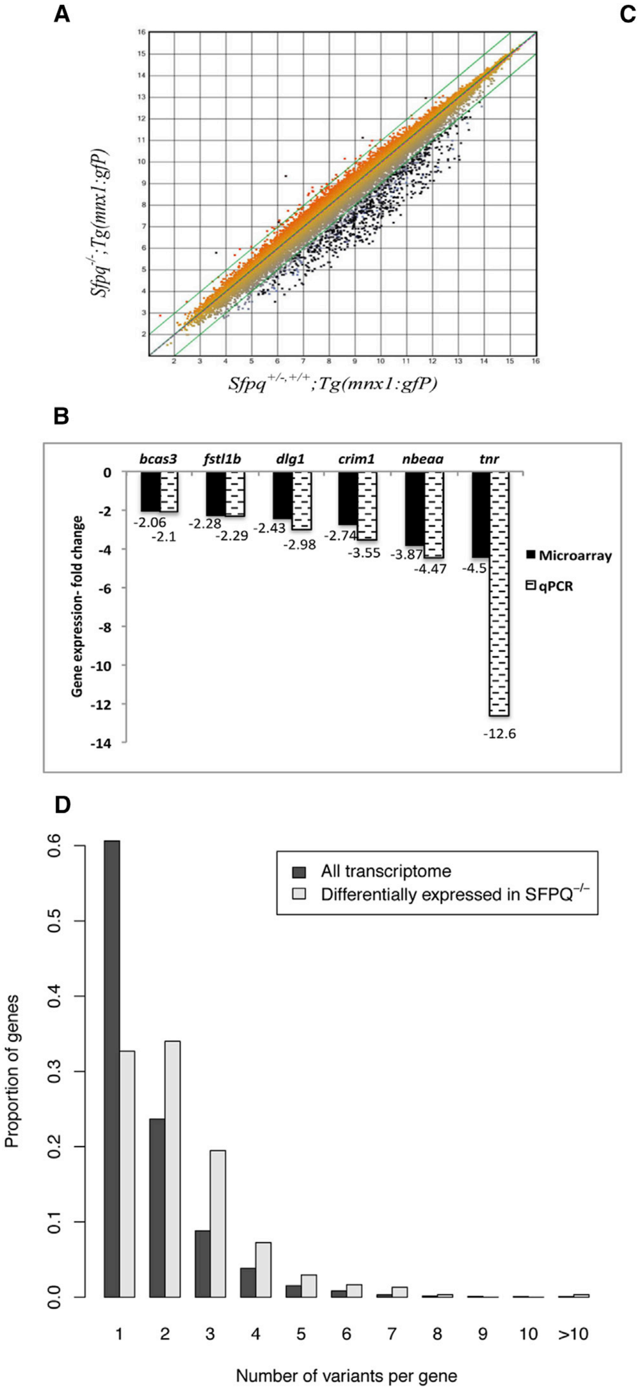

C

GO Terms	GO ID	P-Value	Fold enrichment
**Regulation of signalling**			
Positive regulation of non-canonical Wnt signalling	2000052	0.00000202	19
Ephrin receptor signaling	48013	0.0102	11
Glutamate receptor signaling	7215	0.0000292	9.9
Negative regulation of canonical Wnt signaling	90090	0.00000512	9
Regulation of Ras protein signal transduction	46578	0.0183	2.5
Negative regulation of response to stimulus	48585	0.000531	2.26
Negative regulation of cell communication	10648	0.00276	2.1
**Membrane**			
anchored to membrane	31225	0.0069	15.25
outer membrane-bounded periplasmic space	30288	0.0000177	9.9
cell surface	9986	0.00000528	7.7
integral to plasma membrane	5887	0.00641	3.1
**Receptor activity**			
glutamate receptor activity	8066	0.000118	9.15
transmembrane receptor protein tyrosine kinase activity	4714	0.0013	4.92
**Gated channel activity**	22836		
excitatory extracellular ligand-gated ion channel activity	5231	0.000952	5.2
ligand-gated ion channel activity	15276	0.000359	4.8
ion gated channel activity	22839	0.00277	3.4
**Synaptic part**			
postsynaptic membrane	45211	0.00249	4.03
**Regulation gene expression**			
regulation of RNA splicing	43484	0.0213	3.6
**Growth**			
developmental growth involved in morphogenesis	60560	0.000852	3.41
axis elongation	3401	0.0027	2.6
**Cell junction**	**30054**	**0.00049**	3.4
**Biological Adhesion**			
Cell adhesion	7155	0.000928	3.33
**Synapse**	**45202**	**0.0054**	3.2

Figure 4. Sfpq Loss of Function Affects a Specific Set of Transcripts

(A) Scattered plot of the expression level of the total probe sets in the array. The 38,489 dots represent total individual probes for genes or expressed sequence tags (ESTs). The gene expression level is expressed in relative arbitrary units of fluorescence intensity on a logarithmic scale. Genes whose expression level is identical between the two groups lie on the diagonal line and the non-identical ones lie outside of the two lines parallel to the central diagonal purple line. The dots positioned above the upper diagonal line represent probe sets that are upregulated and the dots appearing below represent probe sets that are downregulated in the mutants as compared to siblings. Black dots represent those 571 probes with expression level at least 2-fold significantly different ($p < 0.05$, moderated t test) between *sfpq*$^{+/-;+/+}$; (*Tg:mnx1 gfp*) and *sfpq*$^{-/-}$;(*Tg:mnx1 gfp*) embryos. A high correlation ($R^2 = 0.98$) was observed for the linear regression performed on the gene set.

(B) Validation of microarray results by qRT-PCR performed on randomly picked downregulated transcripts. The gene expression data from microarray and qPCR exhibited high correlation (Spearman's rho test; $r(6) = 1$, $p = 0.01$). Statistical comparison was performed using paired t test with Bonferroni correction for multiple

(legend continued on next page)

explained by residual nuclear localization of hsfpqΔNLS, as the threshold required for rescue by injection of the wild-type form is well above detection level (Table S4) and mosaic co-injection of pmnx1:GFP and pCS2:hsfpqΔNLS DNA in the mutant shows axonal expression of SFPQ in restored axons, with nuclei lacking detectable hSFPQ protein (Figures 6E and 6F; n = 14; Figure S3A).

The motor rescue obtained with the hsfpqΔNLS is accompanied by a strong morphological phenotype (short body axis and variable cyclopia) likely induced by cytoplasmic accumulation of SFPQ in the developing embryo. This phenotype is observed in homozygous, but not in heterozygous and wild-type siblings (Figures 6A–6D). The absence of phenotype in siblings is explained by the presence of GFP-hsfpqΔNLS in their nuclei, due to correct transport elicited through complex with endogenous SFPQ (inset in Figure 6D). Finally, qPCR done on extracts from control and hsfpqΔNLS-injected $sfpq^{+/-}$ progeny for ten randomly picked neuronal SFPQ-dependent transcripts shows rescue of expression level for six of these by the ΔNLS injection (Figure 6M), demonstrating a substantial impact of the cytoplasmic protein on transcript levels. The six transcripts rescued are all connected to axonal and synaptic functions, and we found intron retention for three of them (bcas3, nbeaa, and tnr) in wild-type neurites. All together, these results show that cytoplasmic SFPQ is sufficient to establish motor connectivity and to protect key axonal transcripts from degradation.

Human SFPQ Mutations Found in ALS Patients Affect SFPQ Localization and Induce Abnormal Motor Axon Morphology

Exome sequencing of the human *SFPQ* gene (Refseq ID NM_005066) in DNA samples from 151 index familial ALS (fALS) patients revealed two independent sequential missense mutations, N533H (c.1597A>C, we have called hsfpqN) and L534I (c.1600C>A, we have called hsfpqL). These mutations affect adjacent residues within the second coiled-coiled domain of the molecule (residues 528–555; Figure 7M) in a region fully conserved from fish to mammals (Figure S3C). This coiled-coil domain has been identified as being essential for the polymerization of SFPQ dimers, paraspeckle formation, and transcript processing (Lee et al., 2015). From the ExAC database, we know that many missense variants are found in normal individuals (Figure 7L). Both mutations are present in heterozygous form in the patients and are absent from all public polymorphism databases (~66,000 individuals), including dbSNP142, 1000 Genomes (http://www.internationalgenome.org/), the Exome Variant Server (http://evs.gs.washington.edu/EVS/), and ExAC (http://exac.broadinstitute.org/). Unfortunately, no family members were available from these kindreds for segregation analysis.

To evaluate the effect of human mutations on the protein function, we tested whether a series of human variants was able to rescue the zebrafish null mutant. We injected mRNA of wild-type or variant alleles and characterized SFPQ protein localization, axonal morphology, and larval motility. In addition to the two ALS-linked mutations, we cloned five other missense variants, choosing those (from ALSDB and ExAC databases; Figures S3B and S3C) that cover various areas of the gene, some only found in normal individuals, others found in both ALS and normal samples (Figure 7M). We quantified the degree of rescue after injection of eight different human transcripts in replicated double-blind experiments. We found that all SFPQ variants are able to broadly rescue the mutant phenotype (including heart defects). Larval motility is normal from its onset and MNs develop at the same pace as wild-type (data not shown). However, when quantifying axon length and shape, we find that the rescued motor axons are often shorter and branch excessively in the hsfpqN and L-injected mutant embryos (Figures 7A–7K, 7Q, and 7R), but not in any injected with other missense variants. Correlated to these defects, detection of SFPQ protein levels is severely reduced in the motor axons of hsfpqN and L-injected mutant embryos and at normal levels in mutants rescued with wild-type and the five other hsfpq variants (Figure 7P). Levels are rarely reduced in the proximal axonal segment near the spinal exit but are pronounced distally (Figures 7N–7O′), indicating that ALS-linked mutations in the coiled-coil domain of the protein may impair the axonal transport of SFPQ.

Altogether, our results show that axonal SFPQ is required for normal motor axonal development, connectivity, and thereby motility in zebrafish. Furthermore, two fALS-linked mutations, lying within the coiled-coil domain of SFPQ, significantly impede its distribution into the distal axon and affect motor axonal branching in zebrafish, showing the importance of the coiled-coil domain in axonal localization and normal motor axon development. Perturbation of motor axon morphology in *sfpq* mutant embryos rescued by fALS-linked variants may suggest a causal link between these two mutations and the human motor degenerative disease. However, segregation data and additional cases carrying mutations in the same residues are needed to support this possibility further.

DISCUSSION

Our study shows that the ubiquitously expressed multi-functional splicing factor SFPQ is functionally required for expression of a restricted set of transcripts during development and carries an essential non-nuclear function in developing motor axons. Moreover, we identify two human *sfpq* point mutations affecting the protein function in zebrafish MNs. We show that these human mutated proteins are affected in their ability to localize in motor axons and to elicit normal motor axon morphology in an endogenous SFPQ null background, while still enabling NMJ formation and normal motility, indicating a likely role for axonal SFPQ in

comparisons. Fold change is expressed as the ratio between the linear expression levels of $sfpq^{+/-;-/-}$; (*Tg: mnx1 gfp*) and $sfpq^{-/-}$;(*Tg:mnx1 gfp*) samples and negative value represents downregulation of gene expression.

(C) GO analysis of the differentially expressed genes.

(D) Analysis of number of splice variants in whole transcriptome and SFPQ-dependent transcriptome. The SFPQ-dependent genes are substantially enriched for high numbers of splice variants (one-sided Wilcoxon-Mann-Whitney rank-sum test with continuity correction, $p = 2.51 \times 10^{-26}$).

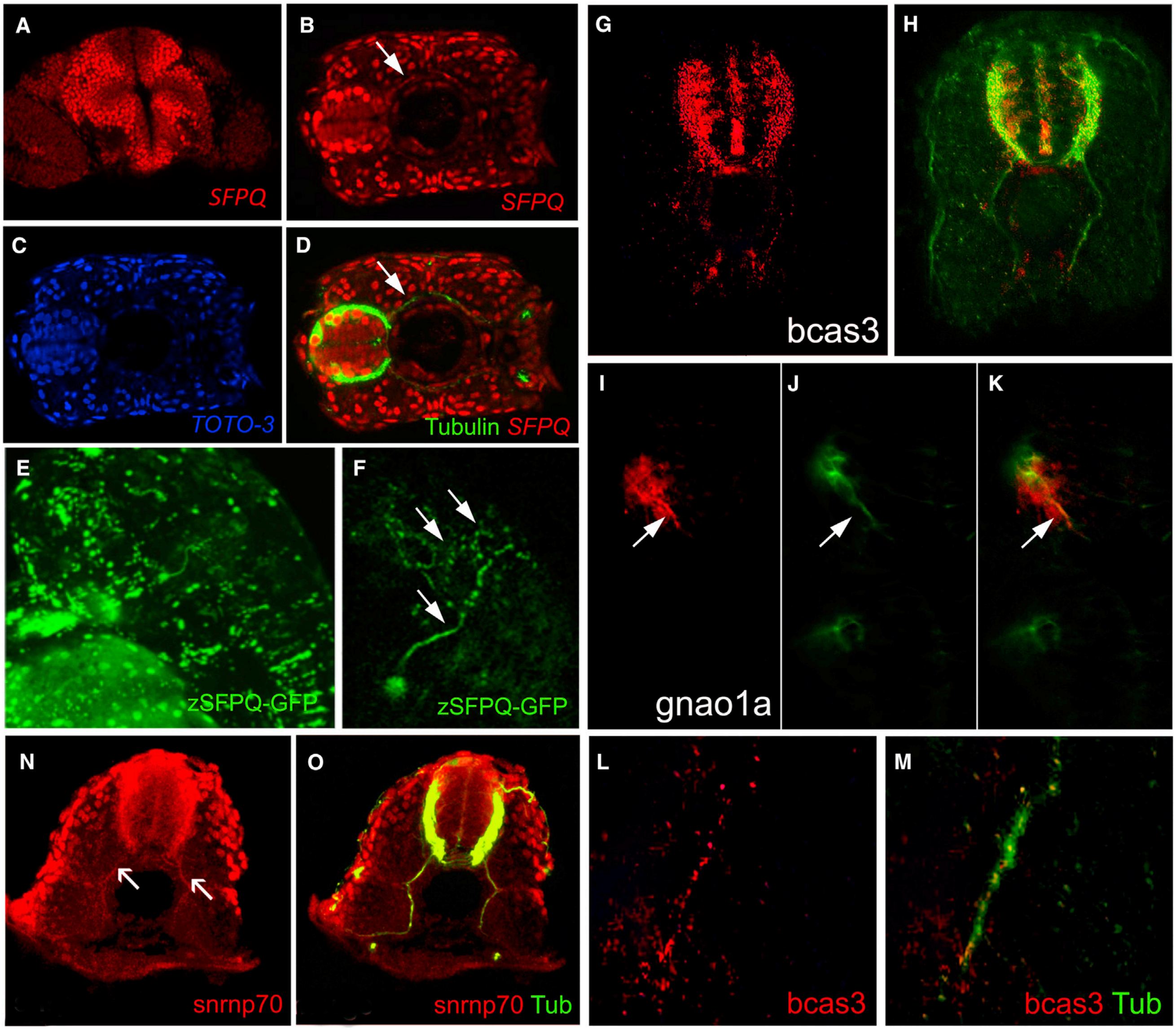

Figure 5. SFPQ Protein Is Found in Axons, with Unprocessed RNA Targets and Spliceosome Component U1 snRNP70

Transverse single confocal sections of 24 hpf (A, N, and O) and 48 hpf (B–M) forebrain (A and I–K) or spinal cord dorsal to the top (G, H, L, and M) or to the left (B–D), and lateral view of head, anterior to the left (E and F).

(A–F) SFPQ protein expression shown by antibody staining (A–D) or GFP-tagged ectopically expressed protein (E and F). Identity of red, green, and blue staining is labeled on bottom right corner of each picture. Arrow shows axonal localization.

(G–K) Localization of first alternative introns (in red) of SFPQ-dependent transcripts, *bcas3* (G and H), and *gnao1a* (I–K). Axons are stained by acetylated tubulin antibody in green.

(L and M) High-resolution imaging of *bcas3* intron detection (L; red) in motor axons stained with acetylated tubulin antibody (M; green).

(N and O) Double antibody staining of U1 snRNP70 proteins (N) with acetylated tubulin (O; merged channels) in spinal axons. Arrows are pointing to motor axons. Scale bar, 25 μm.

human motor function and a possible developmental origin to some forms of motor degeneration.

SFPQ in RNA Transport and Cytoplasmic Processing

SFPQ has been previously described as associated with transport granules in cell culture (Kanai et al., 2004) and very recently shown to be required directly or indirectly for transport of RNA regulon in sensory neurons (Cosker et al., 2016). Our study provides the first in vivo evidence that the cytoplasmic pool of SFPQ is required for axon development through regulation of RNA transport and/or processing in MNs. As transport granules are indeed found to be essential for neuronal function (Kanai et al., 2004), one possible requirement of SFPQ in axon may be transport, as suggested by Cosker and colleagues in cultured sensory

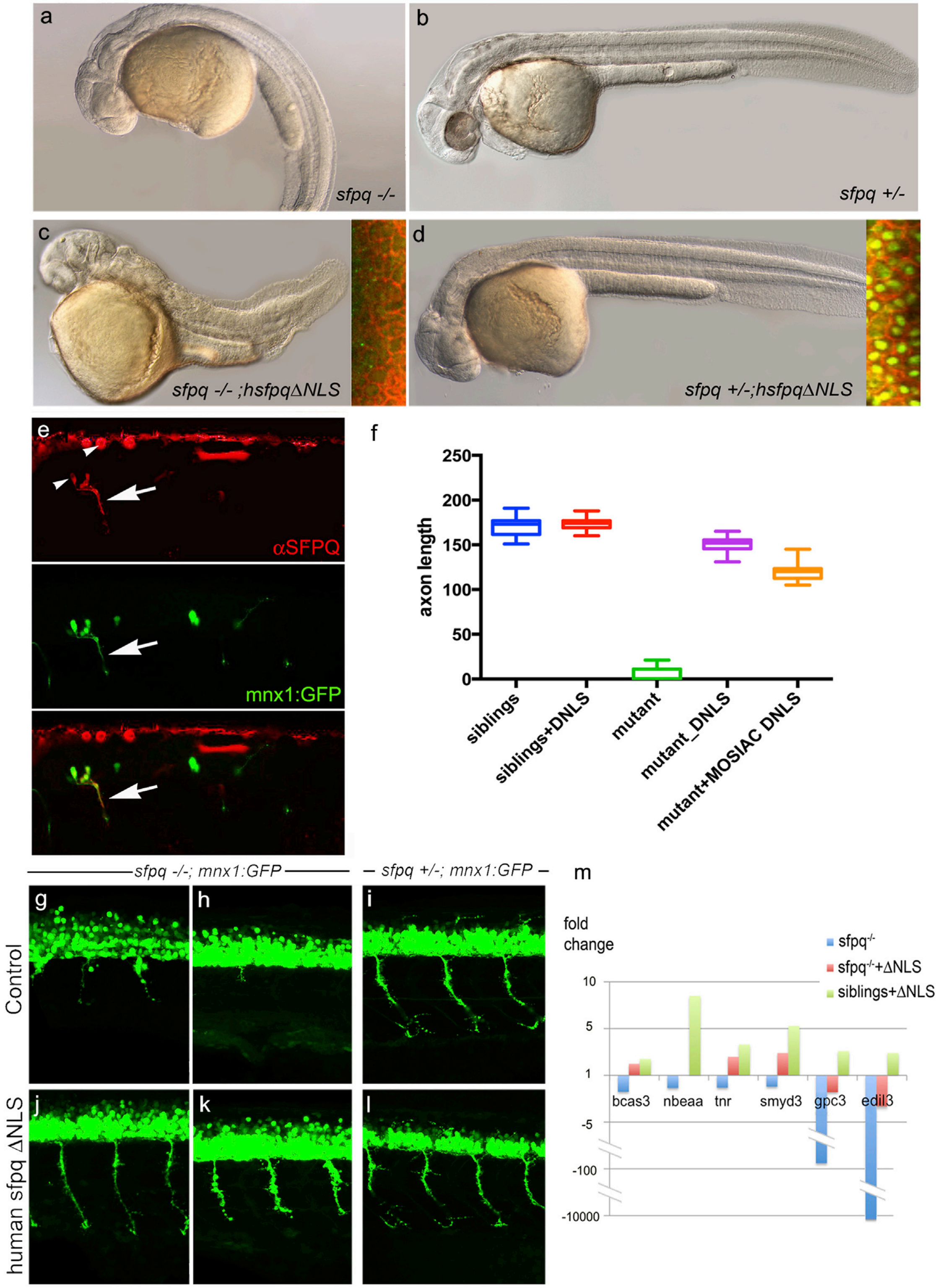

Figure 6. Non-nuclear Sfpq Rescues Loss of Transcripts and Axonal Phenotype in the *coma* Mutant

Lateral view of 30 hpf zebrafish (A–D) and spinal cord (G–L), anterior to the left.

(A–D) General morphology of *sfpq*$^{-/-}$ (A); *sfpq*$^{+/-}$ siblings (B); *sfpq*$^{-/-}$ injected with *GFP-hsfpqΔNLS* (C) and *sfpq*$^{+/-}$ injected with *GFP-hsfpqΔNLS* (D). Insets showing localization of the human GFP-tagged protein in early ss embryos, CAAX-Cherry in red.

(legend continued on next page)

neurons. However, we found that complete loss of SFPQ function, such as the one they induced in sensory neurons, severely affects transcripts coding for transport proteins such as dynein chains (Table S1), making it difficult to distinguish direct from indirect requirement in transport. More compelling is the ability of our cytoplasmic version of SFPQ to rescue motor activity and restore a subset of neuronal transcripts in the zebrafish mutant, strongly demonstrating a cytoplasmic role in transcript maintenance. One such function may be to protect transcripts (intron retaining or not) from nonsense-mediated decay (Yap et al., 2012). Intron retention has been previously reported in neurons (Bell et al., 2010). Our study shows intron retention in developing axons, and detection of U1 snRNP70 spliceosome protein, opening a third possibility: SFPQ may be involved in some form of extra-nuclear processing. Tissue-specific alternative splicing has long been recognized as a powerful source of molecular diversity. This mechanism is particularly important in the nervous system, where isoform variation is involved in synapse formation, neurotransmission, synaptic plasticity, cell recognition, and ion channel function (Grabowski and Black, 2001; Kiebler et al., 2013; Norris and Calarco, 2012). While post-transcriptional regulation commonly occurs in the nucleus, recent studies (Bell et al., 2010; Buckley et al., 2014; Glanzer et al., 2005; König et al., 2007) indicate that modification of 3′ UTR sequences, alternative polyadenylation, and even splicing may be in some situation cytoplasmic, which opens the possibility of sophisticated local axonal and dendritic RNA processing occurring within developing and mature neurons.

The possibility that a subset of transport granules may be specialized for local processing of specific types of introns is a very attractive idea in the context of neurons. Many of the neuronal populations controlling our behaviors have the cell nucleus tens of centimeters away from the site of active synapses. The option of local splicing would be an advantageous mechanism to generate alternative transcripts known to be required for the rapid modulation of synaptic connectivity and other neuronal functions. Our SFPQ mutants are ideal models to now address these possibilities.

SFPQ and Neurodegenerative Disorders

SFPQ plays a key role in processing pre-mRNA coding for proteins that are central to several neurodegenerative conditions. It regulates tau pre-mRNA splicing by suppressing Tau exon 10 inclusion and thus regulating the balance of Tau4R/Tau3R ratio (Ray et al., 2011), associated with tauopathies such as frontotemporal dementia (FTD; Cairns et al., 2007; Connell et al., 2001; Grover et al., 1999; Hasegawa et al., 1998; Hasegawa et al., 1999; Hutton et al., 1998; Mackenzie et al., 2010; Spillantini et al., 1998). SFPQ is expressed at higher levels in the hippocampus and cortex of human brain, the regions principally affected by tauopathy (Ray et al., 2011). The nucleo-cytoplasmic redistribution of SFPQ under pathological conditions is similar to that reported for the RNA-binding proteins, TDP-43 and FUS, in ALS and FTLD (Neumann et al., 2006; Urwin et al., 2010; Vance et al., 2009). Microarray data analysis of transcripts in cortical neurons of Alzheimer's disease (AD) patients identified upregulated *SFPQ* expression (Guttula et al., 2012). In addition to neurodegenerative diseases, SFPQ is also associated with other neurodevelopmental disorders. Recent studies have associated SFPQ and its partner NONO to autism spectrum disorders and intellectual disability, and found SFPQ upregulated in the frontal cortex of bipolar disorder patients (Nakatani et al., 2006; Le-Niculescu et al., 2008).

Our work showed U1-snRNP70 and SFPQ together in motor axons. Intriguingly, two very recent publications showed that this U1 spliceosome protein functionally interacts with the ALS-causative RNA processing protein FUS (Yu et al., 2015; Sun et al., 2015). In these studies, the expectation is that these interact inside the nucleus. However, we find partial co-localization of SFPQ with FUS in axons and FUS has now been found enriched in synapses, suggesting that the synaptic dysfunction observed in FUS mutant may be due to local axonal/synaptic loss of function.

Our work puts a new light on these recent findings, shifting the focus onto cytoplasmic interaction of these proteins and RNAs in the normal context and potential dysregulation of these interactions in neurodegenerative disorders. Neurodegeneration has mostly been understood as caused by sequestration of splicing factors and/or other proteins in cytoplasmic aggregates that induce loss of essential protein function in the nucleus and/or detrimental toxicity in the cytoplasm. However, our data support the intriguing possibility that some neurodegenerative and neurodevelopmental disorders may arise from progressive loss of a cytoplasmic RNA processing function in axons and synapses, triggered during development. Such a mechanism is suggested by a very recent study showing that HNRNP2AB1 is shaping the splicing landscape of neurons, a role specifically affected by ALS-associated mutations in this gene (Martinez et al., 2016). Neuronal homeostasis may be dependent upon a complex balance between nuclear and extra-nuclear RNA processing events controlled by proteins such as SFPQ, HNRNPA2, U1 snRNP70, and FUS, a balance that, when disturbed, may contribute mechanistically to a wide range of neurological disorders.

(E) Whole-mount detection of SFPQ (red) in a 28 hpf *sfpq*$^{-/-}$ mutant, injected at one-cell stage with pmnx1:GFP and pCS2:hsfpqΔNLS DNA, expressing these mosaically. Arrow shows motor axon. Note the negative nuclei (arrowhead).

(F) Measurement of ventral motor axon length in siblings and homozygous mutant uninjected or injected with RNA (DNLS) or DNA (MOSAIC DNLS) coding for hSFPQΔNLS. For the controls and RNA injections, the ventral motor axons were measured in five embryos over five-somite length upstream of the cloaca. For mosaic DNA-injected sample, measures were done only in seven homozygous mutants, in the same trunk area for the rare SFPQ+ neurons.

(G–L) Lateral view, anterior to the left of spinal cords, showing rescue of the axonal defect both in the anterior (G and J) and posterior (H and K) trunk of *sfpq*$^{-/-}$;*Tg (mnx1:GFP)* by injection of the ΔNLS human SFPQ. Sibling axons are unaffected by the injection (I and L).

(M) qPCR results for six out of ten DES transcripts from 32 hpf siblings and *sfpq*$^{-/-}$ mutant embryos uninjected or injected with human ΔNLS *sfpq* RNA. Bars show expression fold changes compared to the expression level of the transcript in uninjected sibling embryos taken as reference. The four transcripts not plotted did not show any improvement after ΔNLS rescue.

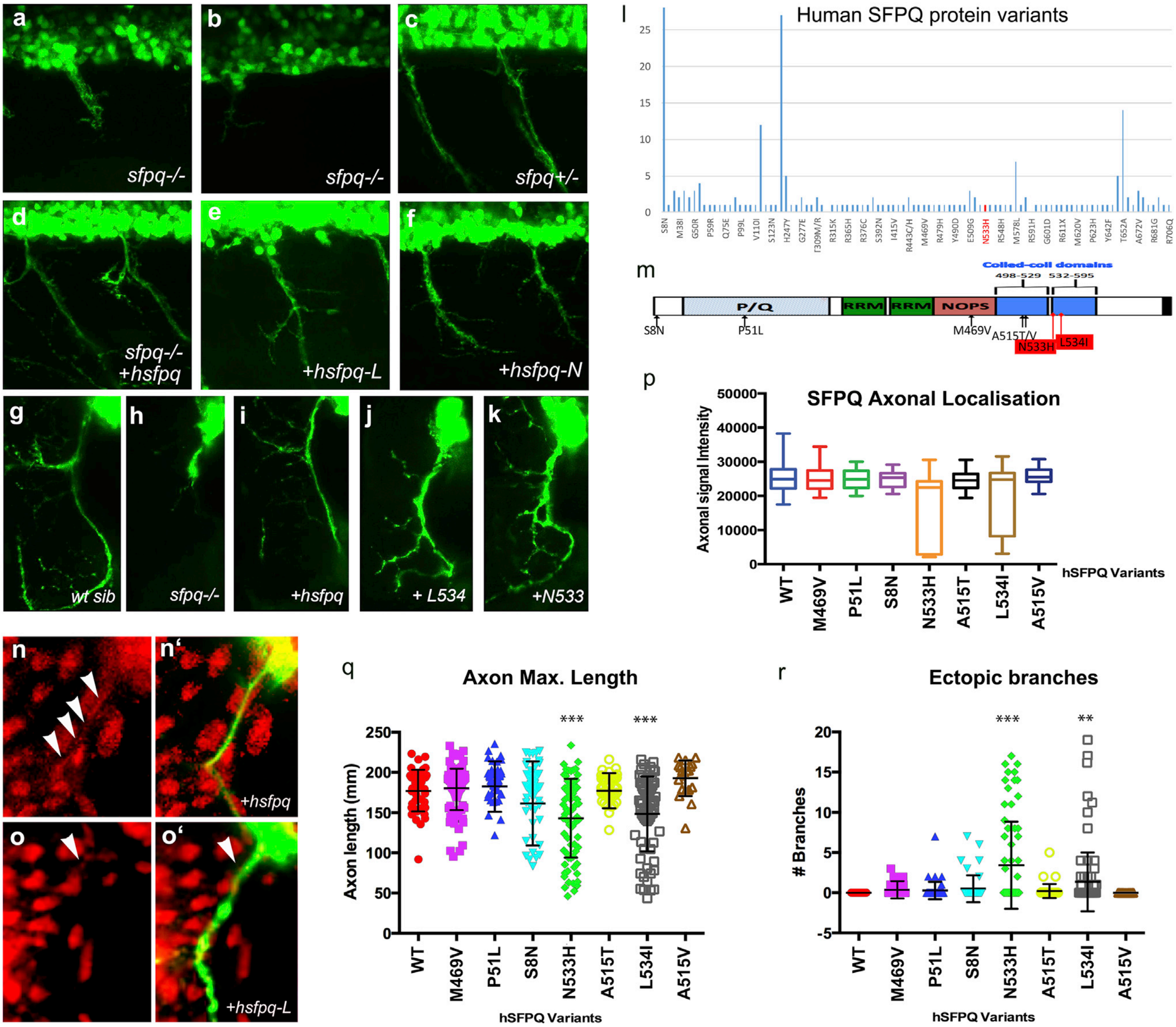

Figure 7. Human Mutations Affect SFPQ Localization and Motor Development in the Zebrafish

(A–K) Lateral (A–F) and transverse (G–K, N, and O) views of the spinal cord in *sfpq*$^{-/-}$ mutant (A, B, and H), siblings (C and G), and homozygous mutant rescued by wild-type human *Sfpq* (D and I) or L534 (E, J, and O) or N533 (F and K) mutant human *Sfpq*.

(L) Distribution of SFPQ variants in all exome-sequenced human individuals. In red, the position of the variants only found in fALS patients.

(M) Schematic of the wild-type hSFPQ protein and the seven variants cloned and tested in double-blind rescue experiments in the Tg(mnx1:GFP) background. S8N (c.23G>A) is present in 4 SALS and 24 ExAC samples, showing a modest enrichment in SALS ($p = 0.029$, two-tailed Fisher's test). P51L (c.152C>T) is private to a single SALS patient and absent from all controls. M469V (c.1405A>G) is in a single SALS patient and twice in ExAC ($p = 0.071$). A515V (c.1544C>T) and A515T (c.1543G>A) are in two and one ExAC normal individuals, respectively, and are located in the coiled-coil domain close to the two FALS variants we identified.

(N and O) Close-up of the motor axon and its environment, stained for SFPQ in red and GFP in green in the *sfpq*$^{-/-}$*; Tg(mnx1:GFP)* rescued by injection of the human (N and N′) or L-mutated form (O and O′). Arrowheads show localization of SFPQ in motor axons.

(P) Quantification of the α-SFPQ red fluorescent signal in motor axons on confocal stacks, for five pairs of motor axons per 48 hpf embryo stained with SFPQ antibody. Measurement was done in no less than 30 (maximum, 46) embryos per variant injected. Injection is done in *sfpq*$^{+/-}$;*Tg(mnx1:GFP)* incross progeny. The embryos showing poor signal intensity were genotyped and were all homozygous mutants.

(Q) Quantification of the length of ventral motor axons. Measures were made for five segments per 48 hpf embryo (five somites anterior to the cloaca) on confocal stacks, using FIJI Single Neurite Tracer in no less than 28 (maximum, 41) embryos per variant injected. Injection is done in *sfpq*$^{+/-}$;*Tg(mnx1:GFP)* incross progeny. The embryos showing shorter axon length were genotyped and were all homozygous mutants. Asterisks indicate highly significant reduction in axon lengths compared to wild-type (pairwise ANOVA, ***$p < 0.0001$). The two significantly different variants show same difference when comparing pairwise to the other variants. All other variants are not significantly different from wild-type.

(legend continued on next page)

STAR★METHODS

Detailed methods are provided in the online version of this paper and include the following:

- KEY RESOURCES TABLE
- CONTACT FOR REAGENT AND RESOURCE SHARING
- EXPERIMENTAL MODEL AND SUBJECT DETAILS
  - Zebrafish Embryos Maintenance
  - Recruitment of Human Subjects
- METHOD DETAILS
  - Genotyping of sfpq mutant
  - Whole mount in situ hybridization and Immunohistochemistry
  - Live imaging
  - Rescue experiments and localization of GFP-tagged SFPQ proteins
  - Mutation screening of Human SFPQ
  - Spinal motor axon mosaic experiment
  - DNA Microarray
  - RT-qPCR
  - Intronic whole mount in situ hybridization
- QUANTIFICATION AND STATISTICAL ANALYSIS
  - Gene Expression and Ontology Analysis
  - Validation of microarray results
  - Splice variant analysis
  - Quantification of neuronal morphological parameters
- DATA AND SOFTWARE AVAILABILITY

SUPPLEMENTAL INFORMATION

Supplemental Information includes three figures, four tables, and three movies and can be found with this article online at http://dx.doi.org/10.1016/j.neuron.2017.03.026.

A video abstract is available at http://dx.doi.org/10.1016/j.neuron.2017.03.026#mmc6.

AUTHOR CONTRIBUTIONS

C.H. designed the research project and supervised and secured funding for the zebrafish team. S.T.-J. and C.H. wrote the manuscript. S.T.-J., T.F., P.M.G., R.T., V.S., H.B., and A.P.-V. performed and conceived experiments in zebrafish, and E.B. and S.T. provided the computational analysis of zebrafish and human genomic datasets, respectively. B.N.S., C.V., C.-H.W., E.P.Mc.C., K.L.W., G.A.N., I.P.B., and C.E.S. generated and provided the patients' genetic data. I.P.B. and C.E.S. supervised and secured funding for the human genetic teams. A.H.F. and C.S.B. shared unpublished data on SFPQ protein structure, and W.S.T. performed the genetic mapping of the zebrafish mutant.

ACKNOWLEDGMENTS

We thank James Patton (Vanderbilt) for the human wild-type and NLS deletion *SFPQ* constructs, and Hazel Sive (MIT) for providing the *whitesnake* zebrafish mutant. We thank Michael Simpson, Division of Genetics and Molecular Medicine, Kings College London, for the assembly and variant calling of the KCL exome data. This work is supported by the Medical Research Foundation (MRF-060-0003-RG) to B.N.S.; the Medical Research Council (G0901525) and the BBSRC (BB/P001599/1) to C.H.; the Wellcome Trust to C.H. and C.E.S. (MC G_1000733); MRC (G0900688) and Motor Neuron Disease Association and Heaton Ellis Trust to C.E.S.; NHMRC of Australia (APP1095215) and MND Australia Leadership grant to I.P.B.; and the MND Research Institute of Australia Grants-in-Aid to I.P.B., K.L.W., and G.A.N.

Received: September 5, 2016
Revised: January 2, 2017
Accepted: March 17, 2017
Published: April 6, 2017; corrected online: May 2, 2017

REFERENCES

Allende, M.L., and Weinberg, E.S. (1994). The expression pattern of two zebrafish achaete-scute homolog (ash) genes is altered in the embryonic brain of the cyclops mutant. Dev. Biol. *166*, 509–530.

Amoyel, M., Cheng, Y.C., Jiang, Y.J., and Wilkinson, D.G. (2005). Wnt1 regulates neurogenesis and mediates lateral inhibition of boundary cell specification in the zebrafish hindbrain. Development *132*, 775–785.

Barry, G., Briggs, J.A., Vanichkina, D.P., Poth, E.M., Beveridge, N.J., Ratnu, V.S., Nayler, S.P., Nones, K., Hu, J., Bredy, T.W., et al. (2014). The long noncoding RNA Gomafu is acutely regulated in response to neuronal activation and involved in schizophrenia-associated alternative splicing. Mol. Psychiatry *19*, 486–494.

Bell, T.J., Miyashiro, K.Y., Sul, J.Y., Buckley, P.T., Lee, M.T., McCullough, R., Jochems, J., Kim, J., Cantor, C.R., Parsons, T.D., and Eberwine, J.H. (2010). Intron retention facilitates splice variant diversity in calcium-activated big potassium channel populations. Proc. Natl. Acad. Sci. USA *107*, 21152–21157.

Brand, M., Granato, M., and Nuesslein-Volhard, C. (2002). Keeping and raising zebrafish. In Zebrafish, a Practical Approach, C. Nuesslein-Volhard and R. Dahm, eds. (Oxford University Press), pp. 7–37.

Buckley, P.T., Khaladkar, M., Kim, J., and Eberwine, J. (2014). Cytoplasmic intron retention, function, splicing, and the sentinel RNA hypothesis. Wiley Interdiscip. Rev. RNA *5*, 223–230.

Cairns, N.J., Bigio, E.H., Mackenzie, I.R., Neumann, M., Lee, V.M., Hatanpaa, K.J., White, C.L., 3rd, Schneider, J.A., Grinberg, L.T., Halliday, G., et al.; Consortium for Frontotemporal Lobar Degeneration (2007). Neuropathologic diagnostic and nosologic criteria for frontotemporal lobar degeneration: consensus of the Consortium for Frontotemporal Lobar Degeneration. Acta Neuropathol. *114*, 5–22.

Carl, M., Bianco, I.H., Bajoghli, B., Aghaallaei, N., Czerny, T., and Wilson, S.W. (2007). Wnt/Axin1/beta-catenin signaling regulates asymmetric nodal activation, elaboration, and concordance of CNS asymmetries. Neuron *55*, 393–405.

Chanas-Sacré, G., Mazy-Servais, C., Wattiez, R., Pirard, S., Rogister, B., Patton, J.G., Belachew, S., Malgrange, B., Moonen, G., and Leprince, P. (1999). Identification of PSF, the polypyrimidine tract-binding protein-associated splicing factor, as a developmentally regulated neuronal protein. J. Neurosci. Res. *57*, 62–73.

Chang, J., Gilman, S.R., Chiang, A.H., Sanders, S.J., and Vitkup, D. (2015). Genotype to phenotype relationships in autism spectrum disorders. Nat. Neurosci. *18*, 191–198.

Cheng, Y.C., Amoyel, M., Qiu, X., Jiang, Y.J., Xu, Q., and Wilkinson, D.G. (2004). Notch activation regulates the segregation and differentiation of rhombomere boundary cells in the zebrafish hindbrain. Dev. Cell *6*, 539–550.

(R) Quantification of ectopic ventral motor branching. Measures were made for five segments per 48 hpf embryo (five somites anterior to the cloaca) on confocal stacks using FIJI Single Neurite tracer in no less than 28 (maximum, 41) embryos per variant injected. Injection is done in *sfpq*$^{+/-}$;*Tg(mnx1:GFP)* incross progeny. The embryos showing excessive branching were genotyped and were all homozygous mutants. Asterisks indicate significance (pairwise ANOVA, ***$p < 0.0001$ and **$p < 0.001$) compared to wild-type. The two significantly different variants show same difference when comparing pairwise to the other variants. All other variants are not significantly different from wild-type.

Connell, J.W., Gibb, G.M., Betts, J.C., Blackstock, W.P., Gallo, J., Lovestone, S., Hutton, M., and Anderton, B.H. (2001). Effects of FTDP-17 mutations on the in vitro phosphorylation of tau by glycogen synthase kinase 3beta identified by mass spectrometry demonstrate certain mutations exert long-range conformational changes. FEBS Lett. *493*, 40–44.

Cooper, M.S., Szeto, D.P., Sommers-Herivel, G., Topczewski, J., Solnica-Krezel, L., Kang, H.C., Johnson, I., and Kimelman, D. (2005). Visualizing morphogenesis in transgenic zebrafish embryos using BODIPY TR methyl ester dye as a vital counterstain for GFP. Dev. Dyn. *232*, 359–368.

Cosker, K.E., Fenstermacher, S.J., Pazyra-Murphy, M.F., Elliott, H.L., and Segal, R.A. (2016). The RNA-binding protein SFPQ orchestrates an RNA regulon to promote axon viability. Nat. Neurosci. *19*, 690–696.

Derrien, T., Johnson, R., Bussotti, G., Tanzer, A., Djebali, S., Tilgner, H., Guernec, G., Martin, D., Merkel, A., Knowles, D.G., et al. (2012). The GENCODE v7 catalog of human long noncoding RNAs: analysis of their gene structure, evolution, and expression. Genome Res. *22*, 1775–1789.

Dorsky, R.I., Sheldahl, L.C., and Moon, R.T. (2002). A transgenic Lef1/beta-catenin-dependent reporter is expressed in spatially restricted domains throughout zebrafish development. Dev. Biol. *241*, 229–237.

Dye, B.T., and Patton, J.G. (2001). An RNA recognition motif (RRM) is required for the localization of PTB-associated splicing factor (PSF) to subnuclear speckles. Exp. Cell Res. *263*, 131–144.

Figueroa, A., Fujita, Y., and Gorospe, M. (2009). Hacking RNA: Hakai promotes tumorigenesis by enhancing the RNA-binding function of PSF. Cell Cycle *8*, 3648–3651.

Flanagan-Steet, H., Fox, M.A., Meyer, D., and Sanes, J.R. (2005). Neuromuscular synapses can form in vivo by incorporation of initially aneural postsynaptic specializations. Development *132*, 4471–4481.

Fox, A.H., Lam, Y.W., Leung, A.K., Lyon, C.E., Andersen, J., Mann, M., and Lamond, A.I. (2002). Paraspeckles: a novel nuclear domain. Curr. Biol. *12*, 13–25.

Ghanem, N., Jarinova, O., Amores, A., Long, Q., Hatch, G., Park, B.K., Rubenstein, J.L., and Ekker, M. (2003). Regulatory roles of conserved intergenic domains in vertebrate Dlx bigene clusters. Genome Res. *13*, 533–543.

Gladden, A.B., Hebert, A.M., Schneeberger, E.E., and McClatchey, A.I. (2010). The NF2 tumor suppressor, Merlin, regulates epidermal development through the establishment of a junctional polarity complex. Dev. Cell *19*, 727–739.

Glanzer, J., Miyashiro, K.Y., Sul, J.Y., Barrett, L., Belt, B., Haydon, P., and Eberwine, J. (2005). RNA splicing capability of live neuronal dendrites. Proc. Natl. Acad. Sci. USA *102*, 16859–16864.

Grabowski, P.J., and Black, D.L. (2001). Alternative RNA splicing in the nervous system. Prog. Neurobiol. *65*, 289–308.

Grigorenko, E.L., Wood, F.B., Meyer, M.S., Pauls, J.E., Hart, L.A., and Pauls, D.L. (2001). Linkage studies suggest a possible locus for developmental dyslexia on chromosome 1p. Am. J. Med. Genet. *105*, 120–129.

Grover, A., Houlden, H., Baker, M., Adamson, J., Lewis, J., Prihar, G., Pickering-Brown, S., Duff, K., and Hutton, M. (1999). 5′ splice site mutations in tau associated with the inherited dementia FTDP-17 affect a stem-loop structure that regulates alternative splicing of exon 10. J. Biol. Chem. *274*, 15134–15143.

Guttula, S.V., Allam, A., and Gumpeny, R.S. (2012). Analyzing microarray data of Alzheimer's using cluster analysis to identify the biomarker genes. Int. J. Alzheimers Dis. *2012*, 649456.

Hasegawa, M., Smith, M.J., and Goedert, M. (1998). Tau proteins with FTDP-17 mutations have a reduced ability to promote microtubule assembly. FEBS Lett. *437*, 207–210.

Hasegawa, M., Smith, M.J., Iijima, M., Tabira, T., and Goedert, M. (1999). FTDP-17 mutations N279K and S305N in tau produce increased splicing of exon 10. FEBS Lett. *443*, 93–96.

Higashijima, S., Hotta, Y., and Okamoto, H. (2000). Visualization of cranial motor neurons in live transgenic zebrafish expressing green fluorescent protein under the control of the islet-1 promoter/enhancer. J. Neurosci. *20*, 206–218.

Hutton, M., Lendon, C.L., Rizzu, P., Baker, M., Froelich, S., Houlden, H., Pickering-Brown, S., Chakraverty, S., Isaacs, A., Grover, A., et al. (1998). Association of missense and 5′-splice-site mutations in tau with the inherited dementia FTDP-17. Nature *393*, 702–705.

Johnson, R. (2012). Long non-coding RNAs in Huntington's disease neurodegeneration. Neurobiol. Dis. *46*, 245–254.

Kanai, Y., Dohmae, N., and Hirokawa, N. (2004). Kinesin transports RNA: isolation and characterization of an RNA-transporting granule. Neuron *43*, 513–525.

Kausalya, P.J., Phua, D.C., and Hunziker, W. (2004). Association of ARVCF with zonula occludens (ZO)-1 and ZO-2: binding to PDZ-domain proteins and cell-cell adhesion regulate plasma membrane and nuclear localization of ARVCF. Mol. Biol. Cell *15*, 5503–5515.

Ke, Y.D., Dramiga, J., Schütz, U., Kril, J.J., Ittner, L.M., Schröder, H., and Götz, J. (2012). Tau-mediated nuclear depletion and cytoplasmic accumulation of SFPQ in Alzheimer's and Pick's disease. PLoS ONE *7*, e35678.

Kiebler, M.A., Scheiffele, P., and Ule, J. (2013). What, where, and when: the importance of post-transcriptional regulation in the brain. Front. Neurosci. *7*, 192.

König, H., Matter, N., Bader, R., Thiele, W., and Müller, F. (2007). Splicing segregation: the minor spliceosome acts outside the nucleus and controls cell proliferation. Cell *131*, 718–729.

Krauss, S., Johansen, T., Korzh, V., and Fjose, A. (1991). Expression of the zebrafish paired box gene pax[zf-b] during early neurogenesis. Development *113*, 1193–1206.

Kubota, M., Kasahara, T., Iwamoto, K., Komori, A., Ishiwata, M., Miyauchi, T., and Kato, T. (2010). Therapeutic implications of down-regulation of cyclophilin D in bipolar disorder. Int. J. Neuropsychopharmacol. *13*, 1355–1368.

Kwan, K.M., Fujimoto, E., Grabher, C., Mangum, B.D., Hardy, M.E., Campbell, D.S., Parant, J.M., Yost, H.J., Kanki, J.P., and Chien, C.B. (2007). The Tol2kit: a multisite gateway-based construction kit for Tol2 transposon transgenesis constructs. Dev. Dyn. *236*, 3088–3099.

Lagier-Tourenne, C., Polymenidou, M., Hutt, K.R., Vu, A.Q., Baughn, M., Huelga, S.C., Clutario, K.M., Ling, S.C., Liang, T.Y., Mazur, C., et al. (2012). Divergent roles of ALS-linked proteins FUS/TLS and TDP-43 intersect in processing long pre-mRNAs. Nat. Neurosci. *15*, 1488–1497.

Le-Niculescu, H., McFarland, M.J., Ogden, C.A., Balaraman, Y., Patel, S., Tan, J., Rodd, Z.A., Paulus, M., Geyer, M.A., Edenberg, H.J., et al. (2008). Phenomic, convergent functional genomic, and biomarker studies in a stress-reactive genetic animal model of bipolar disorder and co-morbid alcoholism. Am. J. Med. Genet. B. Neuropsychiatr. Genet. *147B*, 134–166.

Lee, M., Sadowska, A., Bekere, I., Ho, D., Gully, B.S., Lu, Y., Iyer, K.S., Trewhella, J., Fox, A.H., and Bond, C.S. (2015). The structure of human SFPQ reveals a coiled-coil mediated polymer essential for functional aggregation in gene regulation. Nucleic Acids Res. *43*, 3826–3840.

Lowery, L.A., Rubin, J., and Sive, H. (2007). Whitesnake/sfpq is required for cell survival and neuronal development in the zebrafish. Dev. Dyn. *236*, 1347–1357.

Lukong, K.E., Huot, M.E., and Richard, S. (2009). BRK phosphorylates PSF promoting its cytoplasmic localization and cell cycle arrest. Cell. Signal. *21*, 1415–1422.

Mackenzie, I.R., Neumann, M., Bigio, E.H., Cairns, N.J., Alafuzoff, I., Kril, J., Kovacs, G.G., Ghetti, B., Halliday, G., Holm, I.E., et al. (2010). Nomenclature and nosology for neuropathologic subtypes of frontotemporal lobar degeneration: an update. Acta Neuropathol. *119*, 1–4.

Mariner, D.J., Wang, J., and Reynolds, A.B. (2000). ARVCF localizes to the nucleus and adherens junction and is mutually exclusive with p120(ctn) in E-cadherin complexes. J. Cell Sci. *113*, 1481–1490.

Martinez, F.J., Pratt, G.A., Van Nostrand, E.L., Batra, R., Huelga, S.C., Kapeli, K., Freese, P., Chun, S.J., Ling, K., Gelboin-Burkhart, C., et al. (2016). Protein-RNA networks regulated by normal and ALS-associated mutant HNRNPA2B1 in the nervous system. Neuron *92*, 780–795.

Mercer, T.R., and Mattick, J.S. (2013). Structure and function of long non-coding RNAs in epigenetic regulation. Nat. Struct. Mol. Biol. *20*, 300–307.

Miscimarra, L., Stein, C., Millard, C., Kluge, A., Cartier, K., Freebairn, L., Hansen, A., Shriberg, L., Taylor, H.G., Lewis, B., and Iyengar, S.K. (2007). Further evidence of pleiotropy influencing speech and language: analysis of the DYX8 region. Hum. Hered. *63*, 47–58.

Nakada, C., Satoh, S., Tabata, Y., Arai, K., and Watanabe, S. (2006). Transcriptional repressor foxl1 regulates central nervous system development by suppressing shh expression in zebra fish. Mol. Cell. Biol. *26*, 7246–7257.

Nakatani, N., Hattori, E., Ohnishi, T., Dean, B., Iwayama, Y., Matsumoto, I., Kato, T., Osumi, N., Higuchi, T., Niwa, S., and Yoshikawa, T. (2006). Genome-wide expression analysis detects eight genes with robust alterations specific to bipolar I disorder: relevance to neuronal network perturbation. Hum. Mol. Genet. *15*, 1949–1962.

Neumann, M., Sampathu, D.M., Kwong, L.K., Truax, A.C., Micsenyi, M.C., Chou, T.T., Bruce, J., Schuck, T., Grossman, M., Clark, C.M., et al. (2006). Ubiquitinated TDP-43 in frontotemporal lobar degeneration and amyotrophic lateral sclerosis. Science *314*, 130–133.

Norris, A.D., and Calarco, J.A. (2012). Emerging roles of alternative pre-mRNA splicing regulation in neuronal development and function. Front. Neurosci. *6*, 122.

Okazaki, Y., Furuno, M., Kasukawa, T., Adachi, J., Bono, H., Kondo, S., Nikaido, I., Osato, N., Saito, R., Suzuki, H., et al.; FANTOM Consortium; RIKEN Genome Exploration Research Group Phase I & II Team (2002). Analysis of the mouse transcriptome based on functional annotation of 60,770 full-length cDNAs. Nature *420*, 563–573.

Passon, D.M., Lee, M., Rackham, O., Stanley, W.A., Sadowska, A., Filipovska, A., Fox, A.H., and Bond, C.S. (2012). Structure of the heterodimer of human NONO and paraspeckle protein component 1 and analysis of its role in subnuclear body formation. Proc. Natl. Acad. Sci. USA *109*, 4846–4850.

Patton, J.G., Porro, E.B., Galceran, J., Tempst, P., and Nadal-Ginard, B. (1993). Cloning and characterization of PSF, a novel pre-mRNA splicing factor. Genes Dev. *7*, 393–406.

Peng, R., Dye, B.T., Pérez, I., Barnard, D.C., Thompson, A.B., and Patton, J.G. (2002). PSF and p54nrb bind a conserved stem in U5 snRNA. RNA *8*, 1334–1347.

Postlethwait, J.H., Johnson, S.L., Midson, C.N., Talbot, W.S., Gates, M., Ballinger, E.W., Africa, D., Andrews, R., Carl, T., Eisen, J.S., et al. (1994). A genetic linkage map for the zebrafish. Science *264*, 699–703.

Rabin, M., Wen, X.L., Hepburn, M., Lubs, H.A., Feldman, E., and Duara, R. (1993). Suggestive linkage of developmental dyslexia to chromosome 1p34-p36. Lancet *342*, 178.

Ray, P., Kar, A., Fushimi, K., Havlioglu, N., Chen, X., and Wu, J.Y. (2011). PSF suppresses tau exon 10 inclusion by interacting with a stem-loop structure downstream of exon 10. J. Mol. Neurosci. *45*, 453–466.

Reifers, F., Böhli, H., Walsh, E.C., Crossley, P.H., Stainier, D.Y., and Brand, M. (1998). Fgf8 is mutated in zebrafish acerebellar (ace) mutants and is required for maintenance of midbrain-hindbrain boundary development and somitogenesis. Development *125*, 2381–2395.

Rosonina, E., Ip, J.Y., Calarco, J.A., Bakowski, M.A., Emili, A., McCracken, S., Tucker, P., Ingles, C.J., and Blencowe, B.J. (2005). Role for PSF in mediating transcriptional activator-dependent stimulation of pre-mRNA processing in vivo. Mol. Cell. Biol. *25*, 6734–6746.

Sakhi, S., Bruce, A., Sun, N., Tocco, G., Baudry, M., and Schreiber, S.S. (1994). p53 induction is associated with neuronal damage in the central nervous system. Proc. Natl. Acad. Sci. USA *91*, 7525–7529.

Shanmugalingam, S., Houart, C., Picker, A., Reifers, F., Macdonald, R., Barth, A., Griffin, K., Brand, M., and Wilson, S.W. (2000). Ace/Fgf8 is required for forebrain commissure formation and patterning of the telencephalon. Development *127*, 2549–2561.

Shav-Tal, Y., and Zipori, D. (2002). PSF and p54(nrb)/NonO–multi-functional nuclear proteins. FEBS Lett. *531*, 109–114.

Shav-Tal, Y., Cohen, M., Lapter, S., Dye, B., Patton, J.G., Vandekerckhove, J., and Zipori, D. (2001). Nuclear relocalization of the pre-mRNA splicing factor PSF during apoptosis involves hyperphosphorylation, masking of antigenic epitopes, and changes in protein interactions. Mol. Biol. Cell *12*, 2328–2340.

Shi, S.H., Jan, L.Y., and Jan, Y.N. (2003). Hippocampal neuronal polarity specified by spatially localized mPar3/mPar6 and PI 3-kinase activity. Cell *112*, 63–75.

Solnica-Krezel, L., Schier, A.F., and Driever, W. (1994). Efficient recovery of ENU-induced mutations from the zebrafish germline. Genetics *136*, 1401–1420.

Spillantini, M.G., Murrell, J.R., Goedert, M., Farlow, M.R., Klug, A., and Ghetti, B. (1998). Mutation in the tau gene in familial multiple system tauopathy with presenile dementia. Proc. Natl. Acad. Sci. USA *95*, 7737–7741.

Stamova, B.S., Tian, Y., Nordahl, C.W., Shen, M.D., Rogers, S., Amaral, D.G., and Sharp, F.R. (2013). Evidence for differential alternative splicing in blood of young boys with autism spectrum disorders. Mol. Autism *4*, 30.

Sun, S., Ling, S.C., Qiu, J., Albuquerque, C.P., Zhou, Y., Tokunaga, S., Li, H., Qiu, H., Bui, A., Yeo, G.W., et al. (2015). ALS-causative mutations in FUS/TLS confer gain and loss of function by altered association with SMN and U1-snRNP. Nat. Commun. *6*, 6171.

Sutherland, L.C., Rintala-Maki, N.D., White, R.D., and Morin, C.D. (2005). RNA binding motif (RBM) proteins: a novel family of apoptosis modulators? J. Cell. Biochem. *94*, 5–24.

Tapia-Páez, I., Tammimies, K., Massinen, S., Roy, A.L., and Kere, J. (2008). The complex of TFII-I, PARP1, and SFPQ proteins regulates the DYX1C1 gene implicated in neuronal migration and dyslexia. FASEB J. *22*, 3001–3009.

Thomas-Jinu, S., and Houart, C. (2013). Dynamic expression of neurexophilin1 during zebrafish embryonic development. Gene Expr. Patterns *13*, 395–401.

Tilton, F., and Tanguay, R.L. (2008). Exposure to sodium metam during zebrafish somitogenesis results in early transcriptional indicators of the ensuing neuronal and muscular dysfunction. Toxicol. Sci. *106*, 103–112.

Toyama, R., Curtiss, P.E., Otani, H., Kimura, M., Dawid, I.B., and Taira, M. (1995). The LIM class homeobox gene lim5: implied role in CNS patterning in Xenopus and zebrafish. Dev. Biol. *170*, 583–593.

Tzenova, J., Kaplan, B.J., Petryshen, T.L., and Field, L.L. (2004). Confirmation of a dyslexia susceptibility locus on chromosome 1p34-p36 in a set of 100 Canadian families. Am. J. Med. Genet. B. Neuropsychiatr. Genet. *127B*, 117–124.

Urban, R.J., Bodenburg, Y., Kurosky, A., Wood, T.G., and Gasic, S. (2000). Polypyrimidine tract-binding protein-associated splicing factor is a negative regulator of transcriptional activity of the porcine p450scc insulin-like growth factor response element. Mol. Endocrinol. *14*, 774–782.

Urwin, H., Josephs, K.A., Rohrer, J.D., Mackenzie, I.R., Neumann, M., Authier, A., Seelaar, H., Van Swieten, J.C., Brown, J.M., Johannsen, P., et al.; FReJA Consortium (2010). FUS pathology defines the majority of tau- and TDP-43-negative frontotemporal lobar degeneration. Acta Neuropathol. *120*, 33–41.

Vance, C., Rogelj, B., Hortobágyi, T., De Vos, K.J., Nishimura, A.L., Sreedharan, J., Hu, X., Smith, B., Ruddy, D., Wright, P., et al. (2009). Mutations in FUS, an RNA processing protein, cause familial amyotrophic lateral sclerosis type 6. Science *323*, 1208–1211.

Yap, K., Lim, Z.Q., Khandelia, P., Friedman, B., and Makeyev, E.V. (2012). Coordinated regulation of neuronal mRNA steady-state levels through developmentally controlled intron retention. Genes Dev. *26*, 1209–1223.

Yu, Y., Chi, B., Xia, W., Gangopadhyay, J., Yamazaki, T., Winkelbauer-Hurt, M.E., Yin, S., Eliasse, Y., Adams, E., Shaw, C.E., and Reed, R. (2015). U1 snRNP is mislocalized in ALS patient fibroblasts bearing NLS mutations in FUS and is required for motor neuron outgrowth in zebrafish. Nucleic Acids Res. *43*, 3208–3218.

Zhang, H., and Macara, I.G. (2006). The polarity protein PAR-3 and TIAM1 cooperate in dendritic spine morphogenesis. Nat. Cell Biol. *8*, 227–237.

STAR★METHODS

KEY RESOURCES TABLE

REAGENT or RESOURCE	SOURCE	IDENTIFIER
Antibodies		
acetylated α-tubulin	Sigma, Mouse IgG2b	Cat#T6793; RRID: AB_477585
GFP	Amsbio, Rabbit IgG	Cat#TP401; RRID: AB_10013661
F59	DSHB, Mouse IgG1	Cat# f59; RRID: AB_528373
F310	DSHB, Mouse IgG1	Cat#F310; RRID: AB_531863
phosho-Histone H3	Millipore UK, Rabbit-IgG	Cat#06-570; RRID: AB_310177
ZO-1	Invitrogen, mouse IgG1	Cat#339100; RRID: AB_87181
HuC/D	Molecular Probes	Cat#A21271; RRID: AB_221448
α-bungarotoxin	Invitrogen, Alexa Fluor 488 conjugated	Cat#B13422; RRID: AB_2636927
snRNP70	AvivaSysBio	N/A
SFPQ	AbCam38148	Cat#ab38148; RRID: AB_945424
FastDigest ClaI	Thermo Scientific	Cat#FD0144
CAAX-mcherry RNA	Sean Megason (Scott Fraser) Caltech, CA 91125	N/A
TRIZOL RNA isolation reagent	Invitrogen	Cat#10296010
DNaseI	Ambion TURBO DNase Free kit	Cat#AM1907
Bacterial and Virus Strains		
Zebrafish *sfpq* complete cDNA	Source Bioscience	IMAGE: 6962536
pDONR221 entry clone	Gateway, Invitrogen	Cat#12536017
Critical Commercial Assays		
mMessage Sp6 kit	Ambion	Cat#AM1340
The Gateway cloning system	Invitrogen	Cat#18248013
Quickchange II Site-Directed Mutagenesis Kit	Agilent Technologies	Cat#200523
TOPO TA Expression Kit	Invitrogen	K4810-01
High pure RNA isolation kit	Roche	Cat#11828665001
SuperScript Double-Stranded cDNA Synthesis Kit	Invitrogen	Cat#11917020
First strand CDNA synthesis kit	Thermo Scientific (Molecular Biology)	Cat#K1612
Qiaquick PCR purification kit	QIAGEN	Cat#28104
SYBR Green I Master kit	Roche	Cat#04707516001
NimbleGen 12 × 135 k zebrafish gene-expression arrays	Roche Diagnostics Limited	Cat#05545862001
Deposited Data		
Microarray Data	ArrayExpress	ArrayExpress: E-MTAB-5559
Experimental Models: Organisms/Strains		
Zebrafish: *Tg(isl1:GFP)rw0*	Higashijima et al., 2000	ZFIN ID: ZDB-TGCONSTRCT-070117-161
Zebrafish: *Tg(1.4dlx5a-dlx6a:gfp)ot1*	Ghanem et al., 2003	ZFIN ID: ZDB-TGCONSTRCT-070117-17
Zebrafish: *Tg(TOP:gfp)w25*	Dorsky et al., 2002	ZFIN ID: ZDB-TGCONSTRCT-070117-137
Zebrafish: *Tg(Bactin:HRAS-EGFP)vu119*	Cooper et al., 2005	ZFIN ID: ZDB-TGCONSTRCT-070117-75
Zebrafish: *Tg(Xla.Tubb2b:Hsa.MAPT-GFP))zc1*	Tilton and Tanguay, 2008	ZFIN ID: ZDB-TGCONSTRCT-070117-135

(*Continued on next page*)

Continued		
REAGENT or RESOURCE	SOURCE	IDENTIFIER
Oligonucleotides		
Primers for qRT-PCR	This paper	STAR Methods; qRT-PCR
Software and Algorithms		
ArrayStar4 software	DNASTAR, Madison, USA	https://www.dnastar.com
Gene Annotation: ZFIN	release 9, April 24, 2013	https://zfin.org/
ENSEMBL	release 71	http://www.ensembl.org
Gene Ontology	May 7, 2013	http://www.geneontology.org
R	version 3.1.0	https://www.r-project.org

CONTACT FOR REAGENT AND RESOURCE SHARING

Further information and requests for resources and reagents should be directed to and will be fulfilled by the Lead Contact, Corinne Houart (corinne.houart@kcl.ac.uk).

EXPERIMENTAL MODEL AND SUBJECT DETAILS

Zebrafish Embryos Maintenance

Zebrafish (*Danio rerio*) were maintained at 28°C on a 14 hr light/10 hr dark cycle (Brand et al., 2002). Embryos collected were cultured in fish water containing 0.003% 1-phenyl-2-thiouera to prevent pigmentation and 0.01% methylene blue to prevent fungal growth.

ENU mutagenesis was performed as previously described (Solnica-Krezel et al., 1994). The *sfpq* heterozygous mutant was crossed with a set of GFP transgenic lines (*Tg(isl1:GFP)rw0* (Higashijima et al., 2000), *Tg(1.4dlx5a-dlx6a:gfp)* (Ghanem et al., 2003), *Tg(TOP:gfp)w25*(Dorsky et al., 2002), *Tg(Bactin:HRAS-EGFP)vu119* (Cooper et al., 2005), *Tg(Xla.Tubb2b:Hsa.MAPT-GFP))zc1* (Tilton and Tanguay, 2008) and *Tg(mnx1:GFP)ml2/+(AB)* (also known as Tg(*HB9: gfp*); Flanagan-Steet et al., 2005). Embryos collected from incross of heterozygous mutant carriers from these transgenic mutant lines were selected for the transgene based on GFP fluorescence.

The animal experimentations have been authorized by KCL Ethic Review Committee and under the HO license 70/7577.

Recruitment of Human Subjects

Patients were recruited from the Local ALS Clinic of Kings College London, Denmark Hill, UK and through neurogenetic clinics at Concord Hospital, Sydney, Australia, under informed written consent. Patients were diagnosed with definite or probable ALS according to El Escorial criteria, and had a family history of ALS and/or FTD.

METHOD DETAILS

Genotyping of sfpq mutant

The homozygous mutant embryos were selected from offspring of incross of heterozygous carriers based on the brain and motility phenotype at 28 hpf. Genomic DNA was separately extracted from 300 homozygous embryos and same number of siblings by standard protocol. The mutation was mapped by PCR amplification with random amplified polymorphic DNA (RAPD) and simple sequence length polymorphism (SSLP) markers on a section of zebrafish chromosome 19. Linkage analysis and subsequent candidate gene approach indicated ‘*sfpq’* as the gene mutated in the ENU mutant under study (Postlethwait et al., 1994).

Whole mount in situ hybridization and Immunohistochemistry

Whole-mount in situ hybridization was performed as described elsewhere (Thomas-Jinu and Houart, 2013). The following antisense RNA probes were used: *pax2.1* (Krauss et al., 1991), *fgf8* (Reifers et al., 1998), *foxb1.2* (Amoyel et al., 2005; Nakada et al., 2006), *EphA4*[17], *zash1a* (Allende and Weinberg, 1994), *rfng* (Cheng et al., 2004), *lhx5* (Toyama et al., 1995) and *axin2* (Carl et al., 2007). TUNEL (terminal deoxynucleotide (TdT) dUTP nick labeling) assay was performed using ApopTag peroxidase In situ Detection Kit (Chemicon Cat# S7100) to identify apoptotic cells. Digoxigenin-labeled DNA fragments in the assay were detected by antibody conjugated to alkaline phosphatase (instead of anti digoxigenin-HRP recommended by manufacturer’s protocol) and color reaction was carried out using NBT/BCIP.

Transgenic GFP embryos used for in situ hybridization with digoxigenin labeled antisense RNA probes were stained with Fast Red (Roche) prior to GFP immunostaining. Immunostaining was carried out as previously described (Shanmugalingam et al., 2000). Primary antibodies and dilutions used were as follows: acetylated α-tubulin (Sigma, Mouse IgG2b, 1:1000), GFP (Amsbio, Rabbit IgG,

1:500), F59 (mouse IgG1, 1:10), F310 (mouse IgG,1:10), phosho-Histone H3 (Millipore UK, Rabbit-IgG, 1:500), ZO-1 (Invitrogen, mouse IgG1,1:500), HuC/D (1:200; Molecular Probes), Alexa Fluor 488 conjugated α-bungarotoxin (10 μg/ml PBS, 30 min incubation, Invitrogen), snRNP70 (1:100, AvivaSysBio), SFPQ (1:300, AbCam38148). At least 20 embryos were used per experimental sample per probe and per genotype. Images were acquired with a Nikon eclipse E800 microscope for in situ hybridization and confocal laser scanning microscope (Nikon Eclipse 80i) for immunostaining and time lapse imaging. Images were adjusted for brightness and contrast using Adobe Photoshop 8.0 and cell count and videos were processed using ImageJ software. Cell counts of proliferating cells were performed by a person blind to the genotype of the embryos on Z stack image of WT and coma embryos. For statistical analysis, two-tailed Student's t test was performed using Microsoft Excel.

Live imaging

Time-lapse study was performed to investigate the mutant axonogenesis defects by live imaging a *sfpq*$^{-/-}$; *Tg(mnx1:GFP)* embryos from 20hpf until 72hpf. The primary motor axon initiates exit at 17hpf and reaches the target during the second day of embryonic development in WT. Images were taken every 10 min for up to 2h at different stages of growth on a Nikon E80 confocal microscope. The embryos were anesthetized in 0.02% tricaine, embedded in 1% low melting point agarose dissolved in E2 embryo medium in a 35 mm tissue culture plastic dish and filled with E3 medium for confocal imaging. The embryos were removed from the agarose after imaging, maintained in E2 embryo media at 28°C.

Rescue experiments and localization of GFP-tagged SFPQ proteins

The IMAGE clone (IMAGE: 6962536) of zebrafish *sfpq* complete cDNA sequence in pCMVsport6.1 (Source Bioscience) was sequence verified and stored at –20°C. The plasmid DNA was linearized with FastDigest ClaI (ThermoScientific) and used as template for in vitro synthesis of capped *sfpq* mRNA using the mMessage Sp6 kit (Ambion). The mutant rescue was performed by injecting z-*sfpq* mRNA into one-cell stage embryos derived from the *sfpq−/−;Tg(mnx1:GFP)* incross and *sfpq−/−;Tg(isl1:GFP)* incross. The extent of the rescue was determined by assessing the brain phenotype, motility and the motor neuron expression at 36hpf. A minimum of 180 pg/embryo is required for full phenotypic rescue.

The Gateway cloning system (Invitrogen, Carlsbad, CA) and Tol2 kit (Kwan et al., 2007) was used to clone the full-length z*sfpq* cDNA to generate the expression constructs UAS-zSFPQ-GFP and Hs-zSFPQ-GFP. The GFP tagged human constructs, containing full length GFP-PSF and the GFP-PSF Δ 1-702 (Dye and Patton, 2001), obtained from Prof. James Patton were subcloned into pCS2+ for in vitro transcription of wild-type *hsfpq* and *ΔNLShsfpq*. The human SFPQ L534I and N533H mutant forms were made by site directed mutagenesis according to the manufacturer's protocol (Quickchange II Site-Directed Mutagenesis Kit, Stratagene) using a Gateway pDONR221 entry clone containing WT full length coding SFPQ (Gateway, Invitrogen). These were then subcloned in pcDNA3.1 NT-GFP using the TOPO TA Expression Kit (Invitrogen K4810-01), by PCR amplification from pDonr221 with the primers sfpqORF5′: ATGTCTCGGGATCGGTTCCGG and sfpqORF3′: CTAAAATCGGGGTTTTTTGTTTGG and the Q5 High-Fidelity proof-reading DNA Polymerase (NEB M0491S).

Mutation screening of Human SFPQ

Exome sequencing was performed on 151 index cases from the UK (n = 87) and Australia (n = 64), with an average age-of-onset of 59 years and an average disease duration of 34 months. UK FALS cases were all Caucasian individuals from the South -East of London and Kent. FALS cases from Australia were predominantly of European ancestry. All samples were free from currently known ALS-associated coding mutations and the intronic C9orf72 GGGGCC repeat expansion. Samples were captured using either Nimblegen V3, Illumina TruSeq Exome Enrichment or Agilent SureSelectXT Human All Exon-V5-UTR probes and 100bp paired-end reads sequenced on an Illumina HiSeq-2000. Reads were assembled to the hg19 reference genome achieving an average of 115x coverage across all Refseq coding bases, and variants were called with 'SAMtools Mpileup'. Novel SFPQ variants which were absent from all public variant databases were confirmed by PCR from original stock DNA and directly sequenced using Big-Dye Terminator v1.1 chemistry and an ABI3130 genetic analyzer (Applied Biosystems Pty Ltd, Warrington, UK). Sequence chromatograms were analyzed for mutations using Sequencher 4.10 (Gene Codes Corporation, Ann Arbor, Michigan, USA).

Spinal motor axon mosaic experiment

Progenies from incrosses of *sfpq+/−;Tg(mnx1:mGFP)* were injected with CAAX-mcherry RNA at one cell stage (donor embryos). Mosaic embryos were produced by homotopic and homochronic transplantation into wild-type hosts. Around 20 dorso-posterior epiblast cells collected from late gastrula stage donor embryo (80%–100% epiboly; collected by capillary suction around the posterior midline) were placed at the equivalent location in the same stage wild-type host embryo. The donor and host embryo were identified as *sfpq*$^{-/-}$ or WT sibling based on the MHB and axonogenesis phenotype at 24hpf and identity confirmed by PCR at 36hpf after clonal analysis. Embryos with the transplants that led to the incorporation of the donor cells (mcherry+) in the ventral spinal cord and GFP-positive motor axons were selected for further analysis. Only 25% of the donor clones are homozygous for *sfpq.* We transplanted more than 120 embryos across different days. To assess the behavior of wild-type neurons in mutants, the same approach was taken with *Tg(mnx1:mGFP)* wild-type donors into host embryos from sfpq+/− crosses.

DNA Microarray

A total of six set of embryos (three pools of 50 homozygous *sfpq;Tg(mnx1:GFP)* and WT sibling embryos) were fast-frozen in liquid nitrogen and stored at – 80°C until RNA extraction. All homozygous *sfpq* embryos exhibits impaired motility at 22hpf and this feature was used to segregate mutant embryos from WT siblings for the study. Total RNA was isolated using high pure RNA isolation kit (Roche). Quality and purity was assessed by Nanodrop absorbance ratios and by Agilent Bioanalyzer (BRC genomics core facility, KCL) and used for microarray analysis and subsequent quantitative RT-PCR experiments.

Six RNA pools of 10 μg of total RNA each were used to make double stranded cDNAs, synthesized using the SuperScript Double-Stranded cDNA Synthesis Kit (Invitrogen). These were hybridized separately on NimbleGen 12 × 135 k zebrafish gene-expression arrays. The array contains 38,489 probe sets with up to 3 probes of 60-mer oligonucleotides per gene. The arrays represented at least 24,000 genes plus additional ESTs from multiple zebrafish tissues. The design of the array relied on gene and EST information from several sources; Ensembl 46 (August 2007, Zv7), RefSeq (September 2007), TIGR (Release 14.0), UniGene (Build 54), Vega 27, and ZGC (August 2007).

RT-qPCR

Total RNA was extracted from pools of *sfpq*$^{-/-}$*;Tg(mnx1:GFP)* and *sfpq*$^{+/-;\ +/+}$*;Tg(mnx1:GFP)* embryos at 24hpf using TRIZOL RNA isolation reagent (Invitrogen). cDNA was synthesized (First strand CDNA synthesis kit, GE Healthcare) after treating the RNA isolated with DNaseI (Ambion TURBO DNase Free kit). Products were purified using Qiaquick PCR purification kit (QIAGEN). Reactions were performed using SYBR Green I Master kit (Roche) on a Stratagene MX3005P and data analyzed using MxPro software. Three independent biological replicates of the WT sibling and mutant pools were included in the analysis and all reactions were carried out in triplicates. Cycling parameters were as follows, 95°C 10 min for 1 cycle then 95°C 30 s, 60°C 30 s, 72°C 30 s for 40 cycles then 95°C 60 s, 60°C 30 s, 95°C 30 s for all primer sets. Primer sequences used are as follows: *bcas3* (F – 5′ GCATGTGGAGATAATGCCCA 3′, R – 5′ CAGTCCTTCGCCATCAGAAT 3′); *fstlb* (F – 5′ TTCCTCAACTGCCTGAAACC 3′, R - 5′ TTCCTCAACTGCCTGAAACC 3′); *dlg1* (F – 5′ TTGGCTGTGAATGCTGTTTG 3′, R – 5′ GGCTCCCTTGTGATCTCATC 3′); *crim1* (F – 5′ CCACACTGTCCAGATGATCC 3′, R – 5′ CTCGTCCATGTACGTCTTCC 3′); *nbeaa* (F – 5′ GGTGATAACGGTGTTGTGGA 3′, R – 5′ CCCTGAACTGAGAGGAAACG 3′); *tnr* (F – 5′ GGCTCAGTCAACACAGGAAT 3′, R - 5′ TCTCTTGGCTTCCTCTCACT 3′); *smyd3* (F – 5′ AAACTGCCTGTCAAAGTTGC 3′, R – 5′ TCCGTCTTCTCCTCACTCAT 3′); *gpc3* (F – 5′ GCAAAATGGAGGAGCGTTAC 3′, R – 5′ GCATCAGAGCCCAGAATGTA 3′); *edil3* (F – 5′ CCAGCAGATAACAGCCTCAT 3′, R – 5′ TCTTTCCGTCATCGCTGTAG 3′) and *ef1a* (F – 5 TTGAGAAGAAAA TCGGTGGTGCTG 3′, R – 5′ GGAACGGTGTGATTGAGGGAAATTC 3′). The expression levels of the genes selected were normalized to an endogenous control gene *ef1a*.

Intronic whole mount in situ hybridization

Antisense probes against the sequences of intron 2 of the following twenty genes (ncam1b, igsf21a, nbeaa, gria3b, Hpca, gnao1a, cadm4, ank2b, gng7, cacng2a, oprl1, lrp1bb, bcas3, grip2b, TLN2, rbms3, fut8, Chrm3a, dbn1, ctnna2) were made from PCR amplified fragments (5′ primers containing the sp6 promotor sequence) using zebrafish genomic DNA as template. RNA probes of the genes were then made using the standard protocols. Whole mount in situ hybridization for the intronic genes were performed on 24h and 48h embryos using an *InSituPro* automated system (Intavis, Germany).

QUANTIFICATION AND STATISTICAL ANALYSIS

Gene Expression and Ontology Analysis

The gene expression level and folds changes calculated from six separate samples were normalized by quantile normalization and the gene expression values were generated by RMA (Robust Multichip Average) algorithm. The processed microarray data files received from the Nimblegen were subsequently analyzed using ArrayStar4 software (DNASTAR, Inc. Madison, USA). Technical replicates were all highly correlated ($R^2 > 0.97$ on all replicate pairs; cross R^2 test) and the replicate sets were created for *sfpq*$^{-/-}$*;Tg(mnx1:GFP)* and *sfpq*$^{+/-;\ +/+}$*;Tg(mnx1:GFP)* by mean method for subsequent gene expression analyses. The scattered plot of the gene expression levels showed a high linear correlation ($R^2 = 0.98$) between the two groups. Genes were considered differentially expressed when the level of expression change was at least two fold (upregulation or downregulation) in the homozygous *sfpq* embryos as compared to its WT siblings and the difference between the two groups was significant ($p < 0.01$ moderated t test, in which the false discovery rate (FDR) was controlled by the Benjamini Hochberg correction method).

Ontology analysis conducted for the biological interpretation of the differentially expressed genes using ZFIN gene annotation file gave only few annotations as the microarray design is based on ZV7 release. Hence more recent gene annotations were downloaded from ZFIN (release 9, April 24th 2013), and the probe sequences were aligned against the *D.rerio* genome from ENSEMBL release 71. The GO terms and hierarchy was downloaded from Gene Ontology (http://www.geneontology.org) on May 7th 2013. All genes and functional annotations were imported in ArrayStar4 for further analysis, The GO terms with $p < 0.05$ (after Benjamini Hochberg correction) and with percentage of genes per GO term above the percentage of the genes of its major/ first level GO classification is considered as enriched among the differentially expressed genes.

The number of variant transcripts per gene was obtained using the genome provided by ENSEMBL release 75 (Zfin v9, February 2014) (Allende and Weinberg, 1994). The plots and computations were done using R version 3.1.0 (http://www.R-project.org/).

Validation of microarray results

Validation of microarray results by qRT-PCR was performed on six randomly picked transcripts downregulated according to transcriptome analysis. The correlation of the gene expression data from microarray and qPCR was tested using Spearman's Rho test. Statistical comparison was performed using paired t test with Bonferroni correction for multiple comparisons.

Splice variant analysis

Analysis of number of splice variants in whole transcriptome and DES transcriptome was performed using one-sided Wilcoxon-Mann-Whitney rank sum test with continuity correction based on the number of transcripts for each gene.

Quantification of neuronal morphological parameters

Quantification of the $\alpha-$SFPQ fluorescent signal in motor axons was done in double-blind on confocal stacks, for 5 pairs of motor axons per embryo using FiJi. Measurement was done in no less than 30 (max. 46) embryos per variant injected. Quantification of ventral motor axon lengths and branching complexity were made for 5 segments per embryo (always 5 somites anterior to the cloaca) on confocal stacks, using FIJI Single Neurite Tracer in no less than 28 (max. 41) embryos per variant injected. The variants significantly different from wild-type show same difference when comparing pairwise to the other variants (pairwise ANOVA, $p < 0.0001$ and $p < 0.001$ respectively).

DATA AND SOFTWARE AVAILABILITY

The accession number for the microarray datasets reported in this paper is ArrayExpress: E-MTAB-5559.

Neuron, Volume *94*

Supplemental Information

Non-nuclear Pool of Splicing Factor

SFPQ Regulates Axonal Transcripts

Required for Normal Motor Development

Swapna Thomas-Jinu, Patricia M. Gordon, Triona Fielding, Richard Taylor, Bradley N. Smith, Victoria Snowden, Eric Blanc, Caroline Vance, Simon Topp, Chun-Hao Wong, Holger Bielen, Kelly L. Williams, Emily P. McCann, Garth A. Nicholson, Alejandro Pan-Vazquez, Archa H. Fox, Charles S. Bond, William S. Talbot, Ian P. Blair, Christopher E. Shaw, and Corinne Houart

Supplemental Table 1. SFPQ-dependent transcripts with direct homologs in mouse and human.

actn2	IGLON5	TUSC3	cacna1i	efna5b	gpc3	ncam1b	poc1b	sema5a	uvrag
arvcfb	igsf21a	vti1a	cadm1a	enox1	grid2	ncam2	ppm1e	sema5b	VAV2
astn1	iqsec3a	ank2b	cadm1b	epha6	grin3b	nck2b	prcp	SEMA6B	wwox
bcas3	kcnab1	abca2	cadm2a	EPHB1	Gulp1b	NDST2	prkca	slc25a21	xkr7
BRSK2	Lphn1	acap3a	camta1	erbb4a	HS2ST1	neto1l	prkd2	slit3	zgc:65851
cacna2d2	lrp1bb	ADAMTSL3	camta1a	erbb4b	htr2cl1	nfixb	prkg1b	slx4ip	znf385b
cacng2a	magi2	adarb1b	celf5	fam172a	inadl	nitr3c	ptbp3	smyd3	znf804b
cadm4	magi3	adck1	chrm3a	fam184a	Kazn	nitr3d	PTCHD2	snx29	
cask	nbeaa	adka	CLMP	fam189a1	kcnh3	nlgn4a	ptprea	spon1a	
cdh13	ncam1a	agbl4	cntfr	fancd2	kiaa0586	nphp4	ptprga	spon1b	
cdh4	ncanb	akt3a	cntn5	fars2	KIRREL3	ntrk3a	ptprk	stat5.1	
cpne5	necab2	AL935143.2	col23a1	FBXL17	KLHL29	nudt14	ptprua	strbp	
ctnna2	negr1	apba2b	col4a6	fgf13a	lhfpl3	odz2	ptprub	stx8	
ctnnd2a	nlgn3a	ARHGAP42	crfb4	FHOD3	loxl3b	odz3	ptprub	sulf2l	
dbn1	nrxn2a	arhgef12b	crim1	fign	lpp	oprl1	PXN	tbc1d22a	
dgkza	nrxn3b	ARID5B	CU633160	Frmd5	lrfn2	osbp2	rad51b	tbxas1	
dlg1	ntm	asic1b	cux2b	fstl1b	LRFN3	pacrg	ralgapa2	tcf12	
DLG4	olfm2a	asic1c	dab1a	fstl4	lrp8	pard3	ralgps1	thrab	
dlgap1b	opcml	asic2	dacha	fstl5	lrrn2	pard3b	rbfox1	thrb	
dpp6b	pcdh7b	asic4a	dachd	galnt1	macrod2	pcdh15a	rbms3	tmtc2	
**dync1l1**	plekha5	B3GALT1	dapk2	galnt2	magi1b	pcl	rgs6	tnr	
EPB41L1	ptprsa	b3gat1	def8	galt	map2	PDE1A	rims3	TOX2	
fut8	ptprt	bcl2,bcl2	dgat1b	GBF1	mb21d2b	piezo1	robo3	tp53i11b	
gabbr2	rab6ba	bco2b	dip2c	gdf11	megf11	pkib	ror1	tp53i11a	
gnao1a	raph1	becn1	dph1	gfra1a	mgat4c	pkig	rtn4rl1b	trpm3	
gng7	rtn4r	BX890577.1	draxin	gfra4	mid2	pknox2	rxraa	trps1	
gria3b	sfpq	C13H10orf11	ebf1b	ggt1a	mpp7	plch2a	samd10b	tspan9a	
gria4b	slc8a2b	C20orf112	edil3	gli2a	msi2a	PLXNA2	scfd2	unc5da	
grip2b	syt1a	CA10	efcab11	gli3	msi2b	plxnb1a	sdk1b	unc5db	
hpca	TLN2	CABZ01062452	efna2	glra4a	myrip	plxnb2b	sdk2b	ust	

Supplemental Table 2: Enriched GO terms using LAGO

GOID	Term	P-value	Uncorrected P-value	Number annotated	Annotated genes
GO:0007399	nervous system development	1.96E-11	1.73E-14	33	asic2, bcl2, CA10, cdh4, cpne5, dlg1, draxin, efna2, fstl4, gfra1, gli2, hpca, Lphn1, lrfn2, LRFN3, macrod2, ncan, nck2, negr1, nlgn3, nlgn4, ntm, odz2, odz3, opcml, PLXNA2, plxnb1,
GO:0048666	neuron development	7.56E-11	6.68E-14	23	bcl2, cdh4, cpne5, draxin, efna2, fstl4, gli2, lrfn2, LRFN3, nck2, negr1, nlgn3, ntm, odz2,
GO:0061564	axon development	1.45E-10	1.28E-13	17	bcl2, cdh4, draxin, efna2, fstl4, gli2, lrfn2, LRFN3, nlgn3, odz2, PLXNA2, plxnb1, rtn4r, rxra,
GO:0030182	neuron differentiation	1.45E-10	1.28E-13	25	bcl2, cdh4, cpne5, draxin, efna2, fstl4, gli2, lrfn2, LRFN3, nck2, negr1, nlgn3, nlgn4, ntm, odz2, odz3, opcml, PLXNA2, plxnb1, PTCHD2, rtn4r, rxra, sema5, slit3, ust
GO:0051960	regulation of nervous system development	2.51E-10	2.22E-13	20	asic2, bcl2, cdh4, cpne5, dlg1, draxin, fstl4, gli2, Lphn1, negr1, nlgn3, odz3, PLXNA2, plxnb1, PTCHD2, rtn4r, rxra, sema5, slit3, ust
GO:0031175	neuron projection development	2.65E-10	2.34E-13	21	bcl2, cdh4, cpne5, draxin, efna2, fstl4, gli2, lrfn2, LRFN3, nck2, negr1, nlgn3, odz2, odz3,
GO:0007409	axonogenesis	6.04E-10	5.34E-13	16	bcl2, cdh4, draxin, efna2, fstl4, gli2, lrfn2, LRFN3, nlgn3, odz2, PLXNA2, plxnb1, rtn4r,
GO:0048699	generation of neurons	9.48E-10	8.38E-13	25	bcl2, cdh4, cpne5, draxin, efna2, fstl4, gli2, lrfn2, LRFN3, nck2, negr1, nlgn3, nlgn4, ntm, odz2, odz3, opcml, PLXNA2, plxnb1, PTCHD2, rtn4r, rxra, sema5, slit3, ust
GO:0048812	neuron projection morphogenesis	3.42E-09	3.02E-12	17	bcl2, cdh4, cpne5, draxin, efna2, fstl4, gli2, lrfn2, LRFN3, nlgn3, odz2, PLXNA2, plxnb1, rtn4r, sema5, slit3, ust
GO:0022008	neurogenesis	3.91E-09	3.46E-12	25	bcl2, cdh4, cpne5, draxin, efna2, fstl4, gli2, lrfn2, LRFN3, nck2, negr1, nlgn3, nlgn4, ntm, odz2, odz3, opcml, PLXNA2, plxnb1, PTCHD2, rtn4r, rxra, sema5, slit3, ust
GO:0048468	cell development	4.19E-09	3.70E-12	28	bcl2, cacna2d2, cdh4, cpne5, draxin, efna2, fstl4, gdf1, gli2, lrfn2, LRFN3, nck2, negr1, nlgn3, ntm, odz2, odz3, opcml, PLXNA2, plxnb1, PTCHD2, rtn4r, rxra, sema5, slit3, strbp, ust,
GO:0048858	cell projection morphogenesis	5.54E-09	4.90E-12	17	bcl2, cdh4, cpne5, draxin, efna2, fstl4, gli2, lrfn2, LRFN3, nlgn3, odz2, PLXNA2, plxnb1, rtn4r,
GO:0032990	cell part morphogenesis	9.04E-09	7.99E-12	17	bcl2, cdh4, cpne5, draxin, efna2, fstl4, gli2, lrfn2, LRFN3, nlgn3, odz2, PLXNA2, plxnb1, rtn4r,
GO:0048667	cell morphogenesis involved in neuron differentiation	9.45E-09	8.35E-12	16	bcl2, cdh4, draxin, efna2, fstl4, gli2, lrfn2, LRFN3, nlgn3, odz2, PLXNA2, plxnb1, rtn4r, sema5, slit3, ust
GO:0030030	cell projection organization	1.81E-08	1.60E-11	23	bcl2, cdh4, cpne5, draxin, efna2, fstl4, gli2, lrfn2, LRFN3, nck2, negr1, nlgn3, odz2, odz3,
GO:0048589	developmental growth	1.82E-08	1.60E-11	16	bcl2, cacna2d2, cdh4, cpne5, draxin, fstl4, gli2, nlgn3, nlgn4, PLXNA2, plxnb1, rtn4r, rxra,
GO:0000904	cell morphogenesis involved in differentiation	1.83E-08	1.62E-11	17	bcl2, cdh4, draxin, efna2, fstl4, gli2, lrfn2, LRFN3, nlgn3, odz2, PLXNA2, plxnb1, rtn4r, sema5, slit3, ust, znf385
GO:0045664	regulation of neuron differentiation	2.11E-08	1.87E-11	16	bcl2, cdh4, cpne5, draxin, fstl4, gli2, negr1, nlgn3, odz3, PLXNA2, plxnb1, PTCHD2, rtn4r, sema5, slit3, ust
GO:0000902	cell morphogenesis	6.82E-08	6.03E-11	19	bcl2, cdh4, cpne5, dlg1, draxin, efna2, fstl4, gli2, lrfn2, LRFN3, nlgn3, odz2, PLXNA2, plxnb1,
GO:0048588	developmental cell growth	2.74E-07	2.42E-10	10	cdh4, cpne5, draxin, fstl4, nlgn3, PLXNA2, plxnb1, rtn4r, sema5, slit3
GO:0050767	regulation of neurogenesis	3.02E-07	2.67E-10	16	bcl2, cdh4, cpne5, draxin, fstl4, gli2, negr1, nlgn3, odz3, PLXNA2, plxnb1, PTCHD2, rtn4r,
GO:0032989	cellular component morphogenesis	3.17E-07	2.80E-10	19	bcl2, cdh4, cpne5, dlg1, draxin, efna2, fstl4, gli2, lrfn2, LRFN3, nlgn3, odz2, PLXNA2, plxnb1, rtn4r, sema5, slit3, ust, znf385
GO:0010975	regulation of neuron projection development	4.54E-07	4.01E-10	13	cdh4, cpne5, draxin, fstl4, negr1, nlgn3, odz3, PLXNA2, plxnb1, rtn4r, sema5, slit3, ust
GO:1990138	neuron projection extension	6.52E-07	5.76E-10	9	cdh4, cpne5, draxin, nlgn3, PLXNA2, plxnb1, rtn4r, sema5, slit3
GO:0060560	developmental growth involved in morphogenesis	8.38E-07	7.41E-10	10	cdh4, cpne5, draxin, fstl4, nlgn3, PLXNA2, plxnb1, rtn4r, sema5, slit3
GO:0031344	regulation of cell projection organization	1.32E-06	1.17E-09	14	cdh4, cpne5, draxin, fstl4, negr1, nlgn3, odz2, odz3, PLXNA2, plxnb1, rtn4r, sema5, slit3, ust
GO:0050770	regulation of axonogenesis	1.80E-06	1.59E-09	9	cdh4, draxin, fstl4, PLXNA2, plxnb1, rtn4r, sema5, slit3, ust
GO:0060284	regulation of cell development	2.03E-06	1.80E-09	16	bcl2, cdh4, cpne5, draxin, fstl4, gli2, negr1, nlgn3, odz3, PLXNA2, plxnb1, PTCHD2, rtn4r,
GO:0048675	axon extension	2.24E-06	1.98E-09	8	cdh4, draxin, nlgn3, PLXNA2, plxnb1, rtn4r, sema5, slit3
GO:0050793	regulation of developmental process	2.54E-06	2.25E-09	26	asic2, bcl2, cacna2d2, cdh4, cpne5, dlg1, draxin, fstl4, gli2, htr2c, Lphn1, negr1, nlgn3, odz3, olfm2, PLXNA2, plxnb1, PTCHD2, ror1, rtn4r, rxra, sema5, slit3, stat5, ust, znf385
GO:0007155	cell adhesion	3.32E-06	2.93E-09	23	bcl2, cadm2, cadm4, cdh4, cntn5, dlg1, edil3, gli2, Lphn1, LRFN3, megf11, ncan, nck2, negr1, nlgn3, nlgn4, ntm, odz2, odz3, opcml, plxnb1, ptpru, spon1
GO:0022610	biological adhesion	3.56E-06	3.15E-09	23	bcl2, cadm2, cadm4, cdh4, cntn5, dlg1, edil3, gli2, Lphn1, LRFN3, megf11, ncan, nck2, negr1, nlgn3, nlgn4, ntm, odz2, odz3, opcml, plxnb1, ptpru, spon1
GO:0010769	regulation of cell morphogenesis	7.70E-06	6.81E-09	10	cdh4, draxin, fstl4, nlgn3, PLXNA2, plxnb1, rtn4r, sema5, slit3, ust
GO:0048869	cellular developmental process	8.46E-06	7.48E-09	34	bcl2, cacna2d2, cdh4, cpne5, dlg1, draxin, efna2, fstl4, gdf1, gli2, htr2c, Kazn, lrfn2, LRFN3, nck2, negr1, nlgn3, nlgn4, ntm, odz2, odz3, olfm2, opcml, PLXNA2, plxnb1, PTCHD2, ptpru,
GO:0030154	cell differentiation	8.95E-06	7.92E-09	33	bcl2, cacna2d2, cdh4, cpne5, draxin, efna2, fstl4, gdf1, gli2, htr2c, Kazn, lrfn2, LRFN3, nck2, negr1, nlgn3, nlgn4, ntm, odz2, odz3, olfm2, opcml, PLXNA2, plxnb1, PTCHD2, ptpru, rtn4r,
GO:0022604	regulation of cell morphogenesis	1.02E-05	9.02E-09	12	cdh4, cpne5, dlg1, draxin, fstl4, nlgn3, PLXNA2, plxnb1, rtn4r, sema5, slit3, ust
GO:0048638	regulation of developmental growth	1.80E-05	1.59E-08	10	bcl2, cacna2d2, cdh4, cpne5, draxin, fstl4, PLXNA2, rtn4r, sema5, stat5
GO:0051239	regulation of multicellular organismal process	1.94E-05	1.72E-08	27	asic2, bcl2, cacna2d2, cacng2, cdh4, cpne5, dlg1, draxin, fstl4, gfra4, gli2, htr2c, Lphn1, negr1, nlgn3, odz3, PLXNA2, plxnb1, PTCHD2, ror1, rtn4r, rxra, sema5, slit3, stat5, ust, VAV2
GO:2000026	regulation of multicellular organismal development	3.87E-05	3.42E-08	21	asic2, bcl2, cdh4, cpne5, dlg1, draxin, fstl4, gli2, Lphn1, negr1, nlgn3, odz3, PLXNA2, plxnb1, PTCHD2, ror1, rtn4r, rxra, sema5, slit3, ust
GO:0040007	growth	5.25E-05	4.64E-08	16	bcl2, cacna2d2, cdh4, cpne5, draxin, fstl4, gli2, nlgn3, nlgn4, PLXNA2, plxnb1, rtn4r, rxra,
GO:0023052	signaling	0.00010118	8.95E-08	51	akt3, asic2, bcl2, cacna1i, cacna2d2, cacng2, crfb4, dlg1, draxin, efna2, fstl1, fstl4, gabbr2, GBF1, gdf1, gfra1, gfra4, gli2, glra4, gria3, Gulp1, hpca, htr2c, inadl, iqsec3, kcnh3, Lphn1, lrfn2, LRFN3, magi3, nck2, nlgn3, nlgn4, odz2, odz3, PDE1A, PLXNA2, plxnb1, PTCHD2,
GO:0099537	trans-synaptic signaling	0.00010472	9.26E-08	13	asic2, cacna2d2, dlg1, gabbr2, glra4, gria3, htr2c, Lphn1, lrfn2, LRFN3, nlgn3, nlgn4, rims3
GO:0007268	chemical synaptic transmission	0.00010472	9.26E-08	13	asic2, cacna2d2, dlg1, gabbr2, glra4, gria3, htr2c, Lphn1, lrfn2, LRFN3, nlgn3, nlgn4, rims3
GO:0099536	synaptic signaling	0.00010472	9.26E-08	13	asic2, cacna2d2, dlg1, gabbr2, glra4, gria3, htr2c, Lphn1, lrfn2, LRFN3, nlgn3, nlgn4, rims3
GO:0098916	anterograde trans-synaptic signaling	0.00010472	9.26E-08	13	asic2, cacna2d2, dlg1, gabbr2, glra4, gria3, htr2c, Lphn1, lrfn2, LRFN3, nlgn3, nlgn4, rims3
GO:0007154	cell communication	0.00010515	9.30E-08	51	akt3, asic2, bcl2, cacna1i, cacna2d2, cacng2, crfb4, dlg1, draxin, efna2, fstl1, fstl4, gabbr2, GBF1, gdf1, gfra1, gfra4, gli2, glra4, gria3, Gulp1, hpca, htr2c, inadl, iqsec3, kcnh3, Lphn1, lrfn2, LRFN3, magi3, nck2, nlgn3, nlgn4, odz2, odz3, PDE1A, PLXNA2, plxnb1, PTCHD2,
GO:0007411	axon guidance	0.00011775	1.04E-07	9	cdh4, draxin, efna2, gli2, odz2, PLXNA2, plxnb1, sema5, slit3
GO:0097485	neuron projection guidance	0.00012538	1.11E-07	9	cdh4, draxin, efna2, gli2, odz2, PLXNA2, plxnb1, sema5, slit3
GO:0045595	regulation of cell differentiation	0.00013032	1.15E-07	19	bcl2, cdh4, cpne5, draxin, fstl4, gli2, htr2c, negr1, nlgn3, odz3, olfm2, PLXNA2, plxnb1,
GO:0022603	regulation of anatomical structure morphogenesis	0.00020497	1.81E-07	15	bcl2, cdh4, cpne5, dlg1, draxin, fstl4, nlgn3, PLXNA2, plxnb1, ror1, rtn4r, rxra, sema5, slit3, ust
GO:0009653	anatomical structure morphogenesis	0.00029621	2.62E-07	24	bcl2, cdh4, cpne5, dlg1, draxin, efna2, fstl4, gli2, lrfn2, LRFN3, megf11, nlgn3, odz2, odz3, PLXNA2, plxnb1, ror1, rtn4r, rxra, sema5, slit3, ust, VAV2, znf385
GO:0007157	heterophilic cell-cell adhesion via plasma membrane cell adhesion molecules	0.00037265	3.29E-07	5	cadm2, cadm4, cdh4, Lphn1, odz2
GO:0016049	cell growth	0.00053167	4.70E-07	11	bcl2, cdh4, cpne5, draxin, fstl4, nlgn3, PLXNA2, plxnb1, rtn4r, sema5, slit3
GO:0098609	cell-cell adhesion	0.00063662	5.63E-07	15	bcl2, cadm2, cadm4, cdh4, dlg1, gli2, Lphn1, megf11, nck2, negr1, nlgn3, nlgn4, odz2, odz3,

GO:0007267	cell-cell signaling	0.00064232	5.68E-07	19	asic2, cacna2d2, dlg1, draxin, efna2, gabbr2, gli2, glra4, gria3, htr2c, Lphn1, lrfn2, LRFN3,
GO:0061387	regulation of extent of cell growth	0.00064778	5.73E-07	6	cdh4, draxin, fstl4, PLXNA2, rtn4r, sema5
GO:0007165	signal transduction	0.00073198	6.47E-07	47	akt3, asic2, bcl2, cacna1i, cacng2, crfb4, dlg1, draxin, efna2, fstl1, fstl4, gabbr2, GBF1, gdf1, gfra1, gfra4, gli2, glra4, gria3, Gulp1, hpca, htr2c, inadl, iqsec3, kcnh3, Lphn1, magi3, nck2, nlgn3, nlgn4, odz2, odz3, PDE1A, PLXNA2, plxnb1, PTCHD2, ptpru, rab6, ror1, rtn4r, rxra,
GO:0008361	regulation of cell size	0.00106436	9.41E-07	7	cdh4, draxin, fstl4, PLXNA2, rtn4r, sema5, VAV2
GO:0042391	regulation of membrane potential	0.00137687	1.22E-06	9	asic2, bcl2, cacna1i, cacng2, dlg1, kcnh3, nlgn3, nlgn4, rims3
GO:0051962	positive regulation of nervous system development	0.00141819	1.25E-06	10	asic2, bcl2, cdh4, cpne5, gli2, Lphn1, negr1, nlgn3, odz3, plxnb1
GO:0007166	cell surface receptor signaling pathway	0.00196646	1.74E-06	27	bcl2, cacng2, crfb4, draxin, efna2, fstl1, fstl4, gdf1, gfra1, gfra4, gli2, glra4, gria3, Lphn1, nck2, nlgn3, nlgn4, PLXNA2, plxnb1, PTCHD2, ptpru, ror1, rtn4r, sema5, slit3, stat5, VAV2
GO:0040011	locomotion	0.0026817	2.37E-06	18	adarb1, bcl2, cacna1i, cdh4, dach, draxin, efna2, GBF1, gli2, nck2, odz2, PLXNA2, plxnb1,
GO:0051128	regulation of cellular component organization	0.00307511	2.72E-06	22	asic2, bcl2, cdh4, cpne5, dlg1, draxin, fstl4, hpca, Lphn1, nck2, negr1, nlgn3, odz2, odz3, PLXNA2, plxnb1, rtn4r, sema5, sfpq, slit3, ust, vti1
GO:0001558	regulation of cell growth	0.00454863	4.02E-06	9	bcl2, cdh4, cpne5, draxin, fstl4, PLXNA2, rtn4r, sema5, slit3
GO:0040008	regulation of growth	0.00461023	4.08E-06	11	bcl2, cacna2d2, cdh4, cpne5, draxin, fstl4, PLXNA2, rtn4r, sema5, slit3, stat5
GO:0071840	cellular component organization or biogenesis	0.00494186	4.37E-06	44	akt3, asic2, bcl2, cacna2d2, cacng2, cadm2, cdh4, cpne5, dach, dgat1, dlg1, draxin, efna2, fign, fstl4, GBF1, gli2, Gulp1, hpca, inadl, iqsec3, Lphn1, lrfn2, LRFN3, ncan, nck2, negr1,
GO:0045666	positive regulation of neuron differentiation	0.00630851	5.58E-06	8	bcl2, cdh4, cpne5, gli2, negr1, nlgn3, odz3, plxnb1
GO:0030516	regulation of axon extension	0.0073925	6.54E-06	5	cdh4, draxin, PLXNA2, rtn4r, sema5
GO:0008037	cell recognition	0.00741104	6.55E-06	6	cadm2, cadm4, dlg1, nck2, ntm, opcml
GO:0032535	regulation of cellular component size	0.00793873	7.02E-06	9	cdh4, dlg1, draxin, fstl4, nck2, PLXNA2, rtn4r, sema5, VAV2
GO:0010771	negative regulation of cell morphogenesis	0.00869773	7.69E-06	5	draxin, fstl4, nlgn3, rtn4r, sema5
GO:0006928	movement of cell or subcellular component	0.00949934	8.40E-06	19	adarb1, bcl2, cacna1i, cdh4, dach, draxin, efna2, GBF1, gli2, nck2, odz2, PLXNA2, plxnb1, ptpru, rab6, sema5, slit3, stat5, VAV2

Supplemental Table 3: Expression of mature and intronic RNA of neuronal transcripts

	Gene name	Intronic expression	Mature mRNA expression
**ank2b**	Ankyrin 2b	commissures, radial glia, spinal motor axons	Pan-CNS
**bcas3**	Breast carcinoma amplified 3 (cytoskeletal protein)	spinal neurites and telencephalon	CNS, muscle
**cadm4**	Cadherin molecule 4	motor axons	Pan-CNS
**dlg4**	Disc large homolog 4 (=postsynaptic density 95)	commisures, forebrain & spinal neurites	Pan-CNS neurites
**fut8a**	Fucosyltransferase 8		ubiquitous
**gnao1a**	Guanine nucleotide binding protein1a	commissures, diencephalon, spinal	Pan-CNS
**grip2b**	Glutamate receptor interacting protein 2b	muscle, weak neurites in telencephalon	CNS and somitic muscle
**ncam1b**	Neural cell adhesion molecule1b	weak spinal and forebrain neurites	Pan-CNS
**oprl1**	Opiate receptor like 1	weak in all neuronal cell bodies	Pan-CNS
**tln2**	Talin 2 (cytoskeletal)	telencephalon only	Telencephalon, cerebellum
**nbeaa**	neurobeachin	Cell body in spinal cord, hindbrain, sparse in forebrain	Pan-neuronal

Supplemental Table 4: Dosage series of human SFPQ RNA injection in the zebrafish mutant

pg of *h-sfpq* injected per z-sfpq$^{-/-}$ mutant	Axonal rescue	Motility	qPCR rescue	Nuclear GFP detection
25	-	-	-	-
50	-	-	-	+
100	+	55%	+	+
150	+	100%	+	+

Suppl. Figure 1

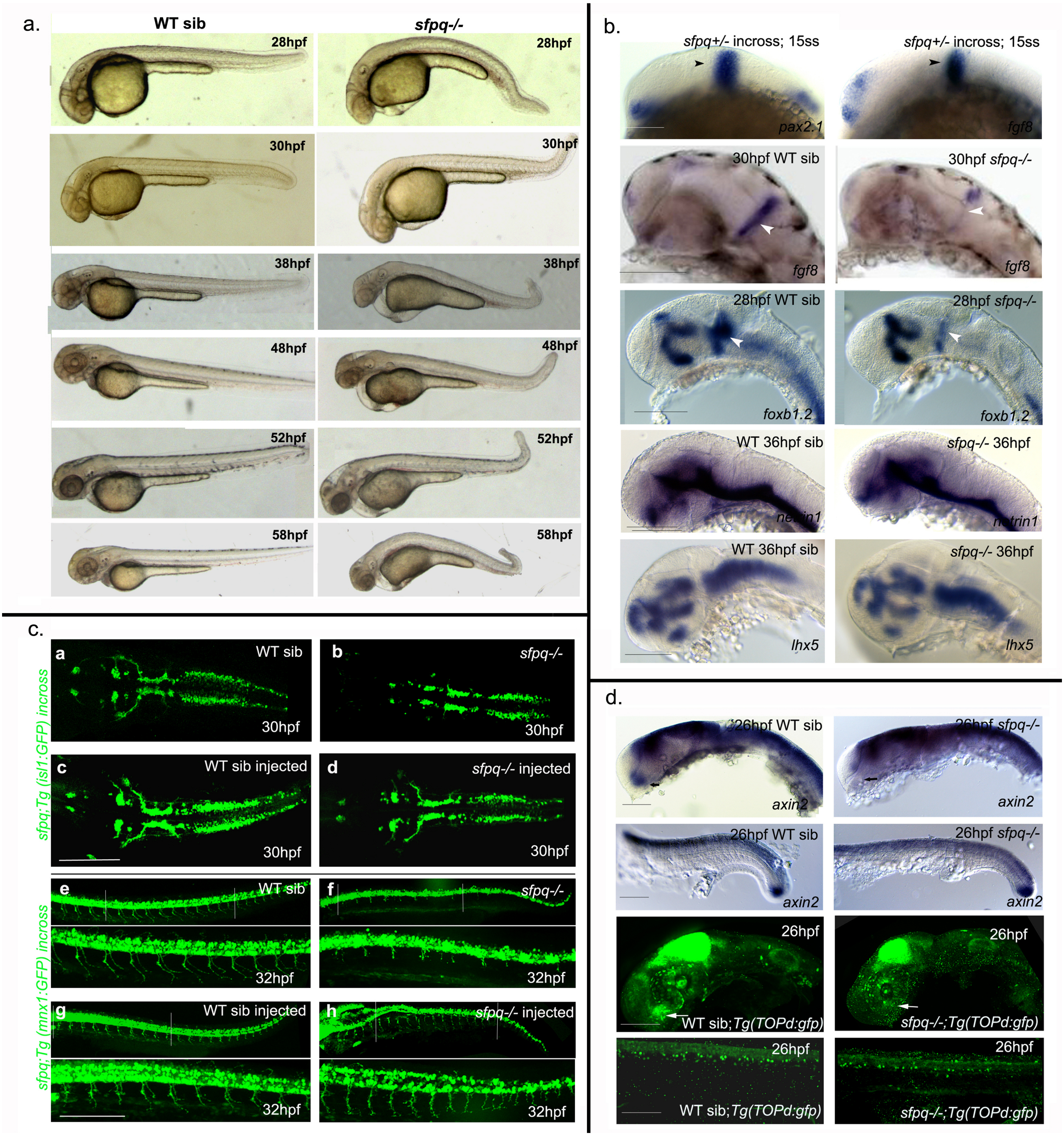

Suppl. Figure 2

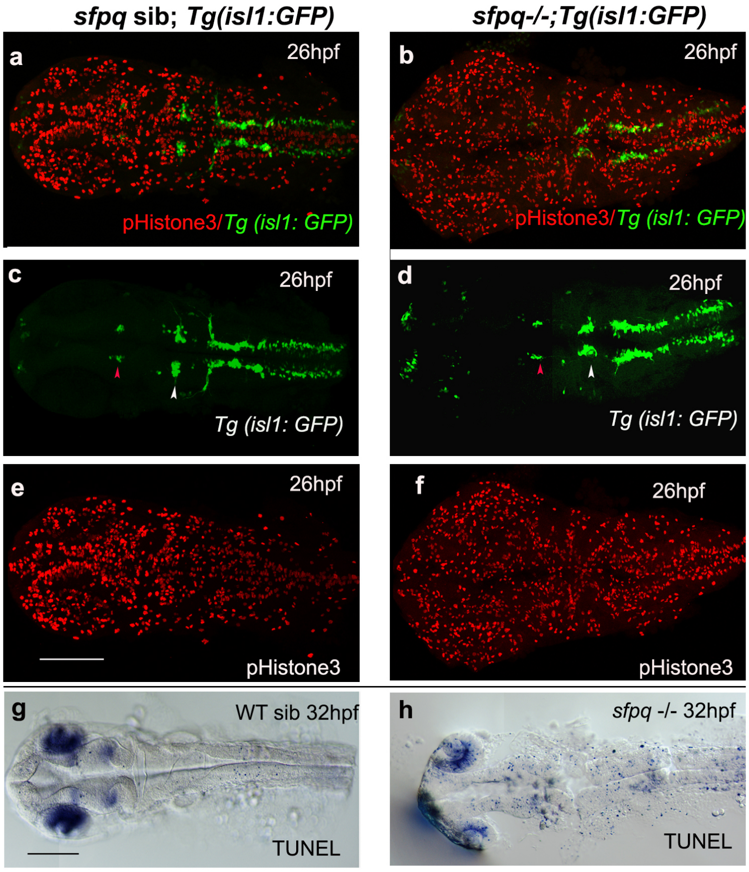

i

Count of pH3 stained cells in *sfpq;Tg(isl1:GFP)* embryos

Stage	Forebrain		Midbrain	Hindbrain			Total	
	Wtsib	*sfpq*	Wtsib	*sfpq*	Wtsib	*sfpq*	Wtsib	*sfpq*
**24hpf**	208	207	212	192	311	192	731	591
	205	117	182	104	214	133	601	354
**26hpf**	296	432	185	225	355	324	836	981
	178	338	144	185	198	340	520	863
	232	176	216	146	292	266	740	588
**48hpf**	234	429	329	321	360	254	923	1004
	207	364	262	459	263	501	732	1324
	214	304	186	466	146	427	546	1197
**56hpf**	325	198	201	169	239	239	765	606
	203	180	257	134	164	239	624	553
	193	174	269	132	192	246	654	553
p-value		**0.28**		**0.84**		**0.36**		**0.39**

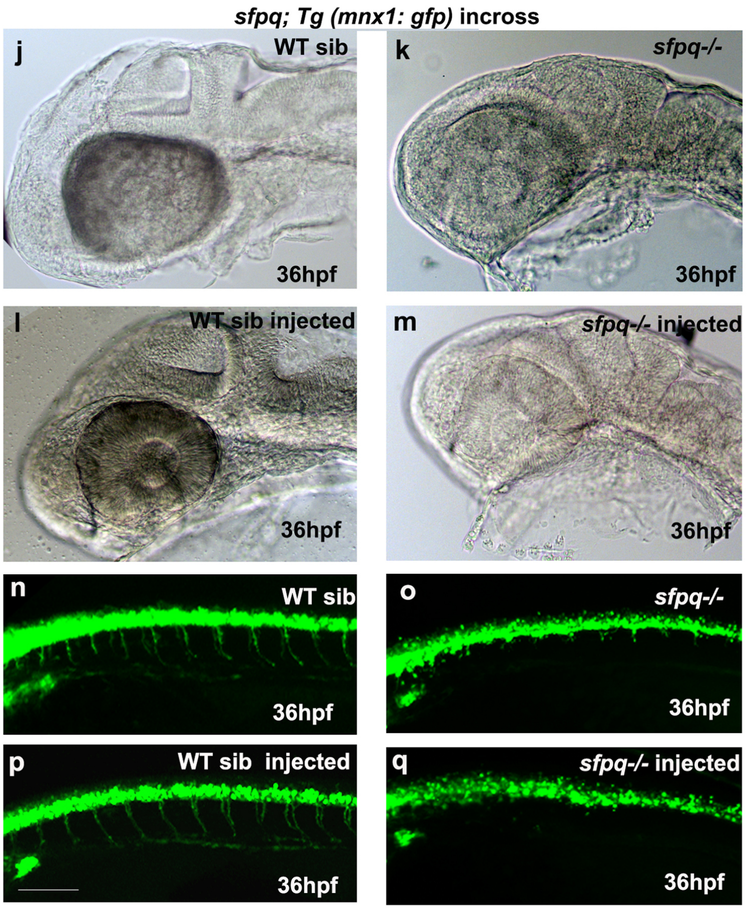

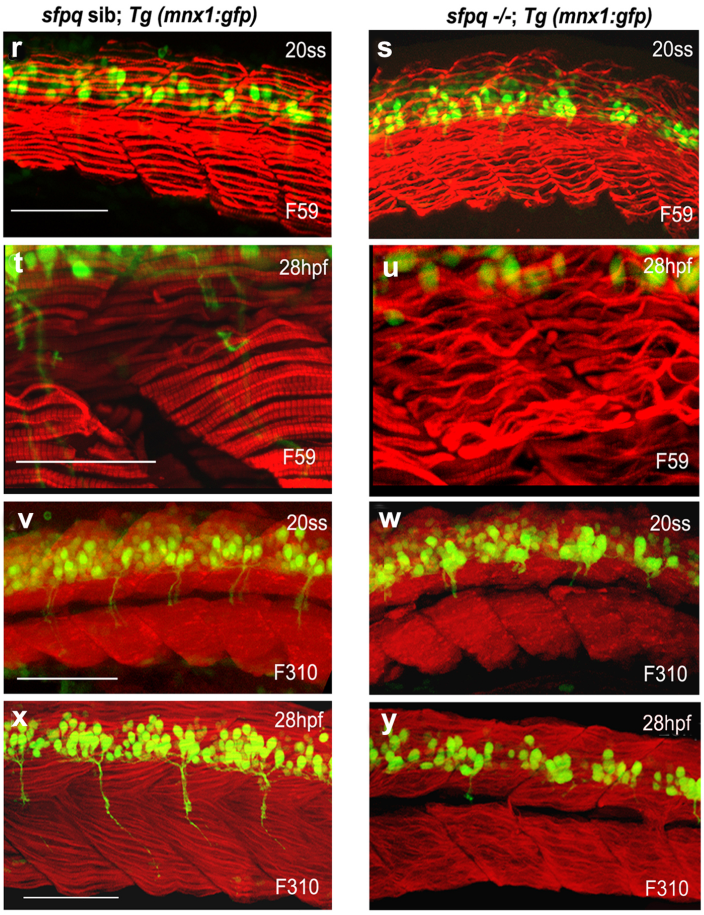

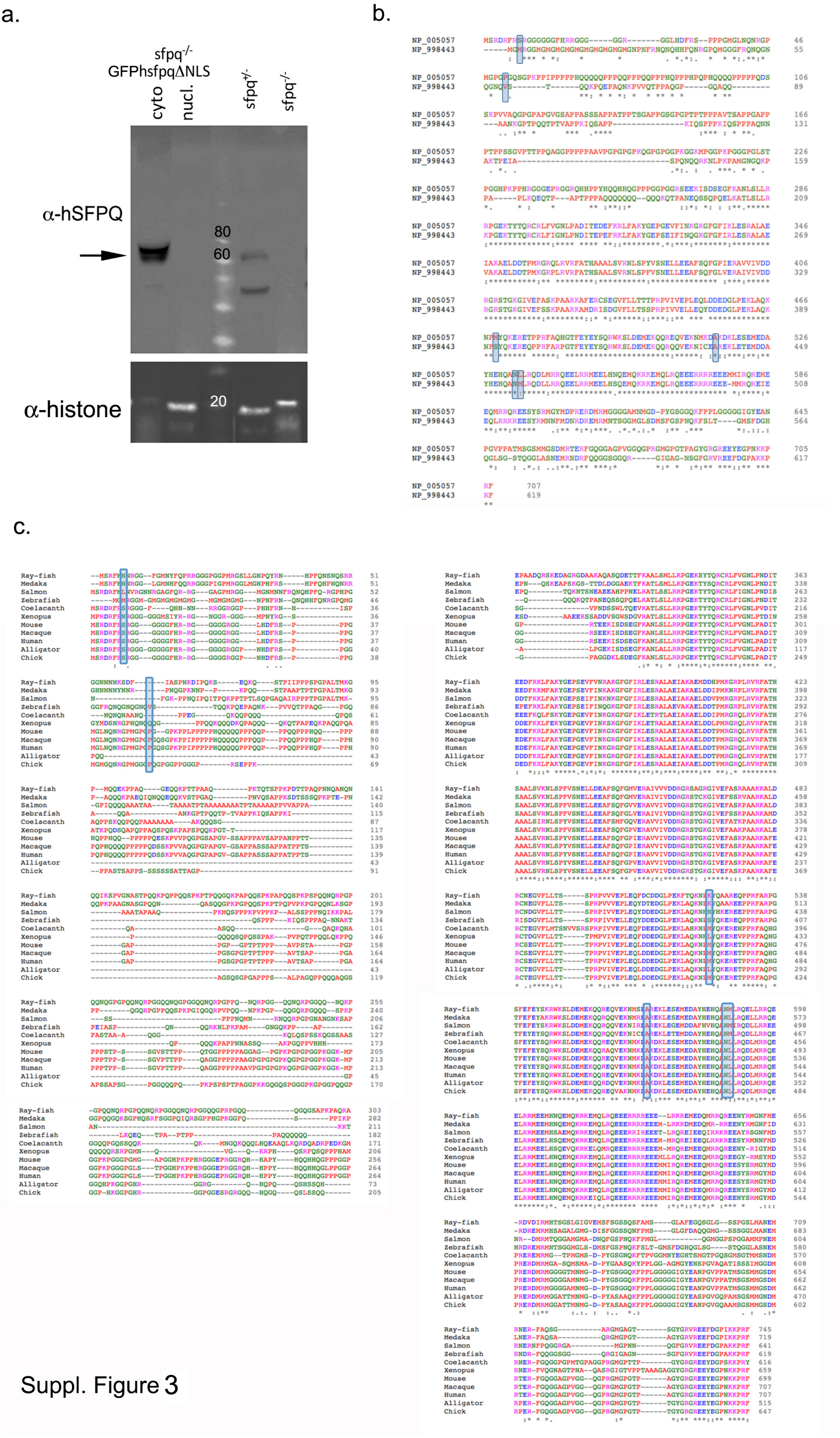

Suppl. Figure 3

Supplemental Table 1 and 2 relates to Figure 4**;** **Table 3** relates to Figure 5 and **Table 4** to Figure 6.

Supplemental Figure 1 (Related to Figure 1)

a. Temporal phenotypic progression in *sfpq* mutant
Lateral view of live WT siblings and *sfpq* mutant with anterior to the left at various developmental stages: 28hpf (n=31), 30hpf (n=47), 38hpf (n=21), 48hpf (n=19), 52hpf (n=41) and 58hpf (n=71), with 25% of the total (n) are homozygous mutants . Scale bar=100µm.
b. Normal overall brain patterning and induction of MHB in *sfpq* mutant
Lateral view of zebrafish brain (**a-l**) and spinal cord (**m,n,q,r**) with anterior to the left. Normal induction of MHB (black arrowhead) is observed in *sfpq* mutants with normal expression of *pax2.1* and *fgf8* at the MHB at 15ss (n=55; n=62). Expression of *fgf8* and *foxb1.2* in the MHB (white arrowhead) is greatly reduced in the *sfpq* mutants (n=10/40; n=5/21) by end of somitogenesis. No difference in the pattern of *netrin1* and *lhx5* expression was detected (n=9/36; n=6/25).
c. Wnt signaling in the sfpq mutant
The *axin2* and *topd gfp* expression was normal in the spinal cord and brain except for its expression (white arrow) in the hypothalamus of WT siblings that is absent in the *sfpq-/-* embryos (n=11/42; n=7/35) showing the absence of Wnt activity in the region. Scale bar=100µm.
c. Rescue of mutant phentotpye with *sfpq* RNA injection
Dorsal view (**a-d**) of zebrafish brain at 30hpf and lateral view (**e-h**) of zebrafish spinal cord at 32hpf with anterior to the left. The embryos from both the *sfpq;Tg(isl1:gfp)* incross (n=51) and the *sfpq;Tg(mnx1:gfp)* incross (n=76) injected with *sfpq* mRNA appeared almost normal compared to its control WT siblings with the rescue of axonogenesis defect and the kinky tail that is observed in the non-injected *sfpq* mutants. Scale bar=100µm.

Supplemental Figure 2 (Related to Figure 2)

Cell proliferation and apoptosis in *sfpq* mutant
Dorsal view of zebrafish brain at 26hpf (**a-f**) and 30hpf (**g-h**) with anterior to the left. **a-f.** Cell proliferation in *sfpq* mutant brain (n=11) analysed using *gfp* expression in *sfpq;Tg:isl1 gfp* as a landmark to delineate the domains: nIII neurons (red arrowhead) as posterior limit of the forebrain and the anterior limit of the midbrain; nV neurons (white arrowhead) as the posterior limit of the midbrain and the anterior limit of the hindbrain. Cell proliferation in the tail of *sfpq* mutant was found in par with that of its WT siblings (data not shown). **g,h.** Increased apoptotis is observed throughout the the *sfpq* brain (n=4) compared to siblings (n=12). **i**. Proliferation quantified using phosphor-Histone3 staining, counted in different brain regions of *sfpq* mutant and siblings. No significant difference was observed in cell count (Student's t-test, p>0.05). Increased cell proliferation observed in some (n=5 of the 11 counted) may be due to cells getting arrested at G2/M phase of cell cycle.
***sfpq* initial phenotype is independent of cell death**
j-q. p53 knockdown in *sfpq;Tg*(*mnx1: gfp*) embryos. Lateral view of zebrafish brain (**j-m**) and spinal cord (**n-q**) at 36hpf with anterior to the left. The p53 MO injected *sfpq*$^{-/-}$ embryos (**k, o.** n=3) show similar MHB phenotype and axonogenesis defect as the non-injected mutants (**m, q.** n=5), compared to injected (**l, p.** n=14) and non-injected siblings

(**j, n.** n=11). Scale bar=100µm.

Muscle formation in *sfpq* mutant

r-y. Lateral view of zebrafish muscle, anterior to the left. **r-u**. Immunostaining for slow muscle with F59 antibody shows proper somatic boundaries and differentiated slow muscle fibres and sarcomeres in the *sfpq* mutant (20ss: n=6/25; 28hpf**:** n=9/39). The wavy appearance of slow muscle fibres in the mutant at later stages compared to its WT siblings (**c-f**), likely due to inactivity. **v-y.** Immunostaining for fast muscle with F310 antibody shows the integrity of the fast muscle in *sfpq* mutant (20ss: n=5/22; 28hpf: n=6/24) as normal as in its WT siblings. Scale bar=100µm.

Supplemental Figure 3 (Related to Figure 6 and 7)

a. Western blots, incubated with either anti-histone H3 or anti-SFPQ, of total extracts from zebrafish siblings and null mutant (right lanes) and of cytoplasmic or nuclear extracts (left lanes) from null mutants injected with GFP-tagged human SFPQ carrying deletion of its C-terminal NLS. Lower band suggests second shorter isoform in zebrafish or cleaved product.

b. Comparison between human (NP_0105057) and zebrafish (NP_998443) protein sequences. Boxes highlight the amino-acids targeted in the human variants chosen for our blind tests.

c. Comparison across species from fish to mammals. Boxes highlight the amino-acids targeted in the human variants chosen for our blind tests.
